# Supplementary material for: Randomised trial of coconut oil, olive oil or butter on blood lipids and other cardiovascular risk factors in healthy men and women
Source: BMJ Open. 2018 Mar 6;8(3):e020167. doi: 10.1136/bmjopen-2017-020167 (PMC5855206; doi:10.1136/bmjopen-2017-020167)
Supplement: Supplementary file 1 [file bmjopen-2017-020167supp001.pdf]

Supplemental Table 1
Mean change in variables between baseline and follow up after dietary interventions and pairwise comparisons between fats in 91 participants
(Adjusting for age, sex and body mass index at baseline)

	Change from Baseline				Pairwise comparisons		
	Coconut oil N=28	Butter N=33	Olive Oil N=30		Coconut oil vs olive oil	Butter vs Coconut oil	Butter vs olive oil
Adjusted for age, sex and body mass index	Mean	Mean	Mean	P value Comparison Between groups	Difference (95% CI)	Difference (95% CI)	Difference (95% CI)
LDL-Cholesterol mmol/L	-0.10	0.34	-0.06	<0.001	-0.05 (-0.28,0.18)	0.45 (0.22,0.68)	0.40 (0.17,0.62)
Total cholesterol mmol/L	0.22	0.42	0.03	0.025	0.19 (-0.09,0.46)	0.19 (-0.08,0.46)	0.38 (0.11,0.64)
HDL-Cholesterol mmol/L	0.29	0.09	0.10	0.008	0.17 (0.04,0.29)	-0.19 (-0.31,-0.06)	-0.02 (-0.14,0.10)
Triglycerides mmol/L	0.08	-0.02	-0.02	0.61	0.09 (-0.13,0.31)	-0.10 (-0.32,0.12)	-0.01 (-0.22,0.20)
Cholesterol/HDL ratio	-0.26	0.10	-0.12	0.001	-0.16 (-0.35,0.03)	0.36 (0.18,0.55)	0.20 (0.02,0.39)
Non HDL-Cholesterol mmol/L	-0.07	0.34	-0.07	<0.001	-0.01 (-0.25,0.23)	0.40 (0.17,0.64)	0.39 (0.16,0.62)
Glucose mmol/L	-0.06	0.02	-0.06	0.66	-0.01 (-0.25,0.23)	0.10 (-0.14,0.34)	0.09 (-0.14,0.33)
C-Reactive Protein mg/L	-0.29	-0.03	0.20	0.14	-0.55 (-1.08,-0.02)	0.31 (-0.22,0.84)	-0.24 (-0.75,0.27)
Weight Kg	0.27	0.05	-0.05	0.40	0.31 (-0.15,0.78)	-0.22 (-0.68,0.24)	0.10 (-0.35,0.54)
Waist cm	1.23	0.25	0.66	0.56	0.23 (-1.45,1.91)	-0.86 (-2.50,0.77)	-0.63 (-2.25,0.98)
Body fat %	0.23	0.36	0.12	0.88	-0.01 (-0.64,0.63)	0.14 (-0.48,0.76)	0.13 (-0.48,0.75)
BMI kg/m2	0.09	0.22	-0.01	0.44	0.10 (-0.06,0.26)	-0.07 (-0.23,0.09)	0.04 (-0.12,0.19)
Systolic blood pressure mm Hg	0.25	-3.68	-3.85	0.30	3.94 (-1.31,9.18)	-3.23 (-8.44,1.98)	0.70 (-4.38,5.79)
Diastolic blood pressure mm Hg	-2.08	-1.28	-0.45	0.75	-0.91 (-4.08,2.25)	1.16 (-1.96,4.29)	0.25 (-2.83,3.33)

Supplemental table 2

Mean change in variables between baseline and follow up after dietary interventions in 71 participants who reported no change in physical activity during the trial

	Change from Baseline			
	Coconut oil N=22 Mean (SD)	Butter N=24 Mean (SD)	Olive Oil N=25 Mean (SD)	P value Comparison Between groups
LDL-Cholesterol mmol/L	-0.10 (0.50)	0.20 (0.53)	-0.04 (0.35)	0.01
Total cholesterol mmol/L	0.19 (0.59)	0.38 (0.63)	0.07 (0.37)	0.13
HDL-Cholesterol mmol/L	0.31 (0.29)	0.10 (0.26)	0.12 (0.16)	0.001
Triglycerides mmol/L	-0.02 (0.46)	-0.01 (0.42)	-0.04 (0.23)	0.97
Cholesterol/HDL ratio	-0.30(0.35)	0.07 (0.44)	-0.13 (0.30)	0.004
Non HDL-Cholesterol mmol/L	-0.11 (0.44)	0.28 (0.56)	-0.06 (0.36)	0.008
Glucose mmol/L	-0.12 (0.49)	-0.02 (0.52)	-0.08 (0.51)	0.80
C-Reactive Protein mg/L	-0.30 (1.18)	-0.13 (0.86)	0.04 (1.00)	0.51
Weight Kg	0.13 (0.62)	0.07 (1.06)	-0.02 (0.76)	0.83
Waist cm	1.47 (3.35)	0.67 (3.48)	0.81 (3.48)	0.70
Body fat %	0.34 (1.11)	0.23 (1.37)	0.81 (1.37)	0.71
Body Mass Index kg/m2	0.04 (0.22)	0.03 (0.37)	0.00 (0.26)	0.85
Systolic blood pressure mm Hg	-3.1 (8.9)	-5.1 (11.3)	-2.4 (7.8)	0.60
Diastolic blood pressure mm Hg	-2.4 (5.6)	-2.0 (6.6)	0.8 (8.4)	0.24
