# Supplementary material for: Randomised trial of coconut oil, olive oil or butter on blood lipids and other cardiovascular risk factors in healthy men and women
Source: BMJ Open. 2018 Mar 6;8(3):e020167. doi: 10.1136/bmjopen-2017-020167 (PMC5855206; doi:10.1136/bmjopen-2017-020167)
Supplement: Supplementary file 2 [file bmjopen-2017-020167supp002.pdf]

Appendix 1: Fatty acid composition of fats

Samples of the fats/oils used in the trial were sent for fatty acid composition to West Yorkshire Analytical Services, a UKAS accredited testing service for food composition. The results are tabulated below.

Coconut oil was 94 % saturated fatty acids, of which the main components were lauric acid C12:0 (48%) and myristic acid C14:0 (19%), palmitic acid C16:0 (9%) and caprylic acid C8:0 (9%); and 5% mono unsaturated fat, mainly oleic acid C18:1n9 (5%).

Butter was 66% saturated fatty acids, of which the main components were palmitic acid C16:0 (28%), stearic acid C18:0 (12%), myristic acid C14:0 (11%); 26% monounsaturated fat, mainly oleic acid C18:1n9 (22%); and 3% polyunsaturated fat, linoleic acid C18:2n6 (2%) and alpha-linolenic acid (1%).

Olive oil was 19% saturated fatty acids, mainly palmitic acid C16:0, 15% with stearic acid C18:0 (3%); 68% monounsaturates with the main component being oleic acid C18:1n9 (64%); and 13% polyunsaturates Linoleic acid C18:2n6 (12%).

		Coconut oil	Olive Oil	Butter
		% composition	% composition	% composition
C4:0	Butyric acid	<1	<0.1	2.5
C6:0	Caproic acid	0.7	<0.1	1.9
C8:0	Caprylic acid	8.6	<0.1	1.2
C10:0	Capric acid	6.3	<0.1	2.5
C12:0	Lauric acid	47.6	<0.1	3
C14:0	Myristic acid	18.6	<0.1	10.6
C14:1		<0.1	<0.1	0.9
C15:0		<0.1	<0.1	1.1
C16:0	Palmitic acid	8.6	14.8	28.1
C16:1	Palmitoleic acid	<0.1	1.5	1.4
C17:0		<0.1	<0.1	0.6
C17:1		<0.1	<0.1	0.4
C18.0	Stearic Acid	3.4	3	12.4
C18:1t			<0.1	3.2
C18:1n9	Oleic Acid	5.2	63.5	22.2
C181n7	cis-Vaccenic Acid	<0.1	2.8	0.4

C18:2tt		<0.1	<0.1	0.5
C18:2ct		<0.1	<0.1	0.1
C18:2tc		<0.1	<0.1	0.2
C18:2n6	Linoleic Acid	0.8	11.9	1.9
C18:3n6	Gamma Linolenic Acid	<0.1	<0.1	<0.1
C18:3n3	Alpha-Linolenic Acid	<0.1	<0.1	0.9
C20:0	Arachidic acid	<0.1	<0.1	0.2
C20:2n6	Eicosadienoic acid	<0.1	<0.1	<0.1
C18:4n3	Stearidonic acid	<0.1	0.2	0.1
C20:1	Paullinic acid	<0.1	<0.1	<0.1
C22:0	Behenic Acid	<0.1	0.2	0.1
C22:1n9	Erucic Acid	<0.1	<0.1	0.1
C22:2	Docosadienoic acid	<0.1	0.6	<0.1
C24:0	Lignoceric acid	<0.1	<0.1	<0.1
	Saturates	93.9	18.6	66.2
	Monounsaturates	5.2	68	26.1
	Polyunsaturates	0.7	13.5	3.4
	Transesters	<0.1	<0.1	4.2
