# Supplementary material for: Randomised trial of coconut oil, olive oil or butter on blood lipids and other cardiovascular risk factors in healthy men and women
Source: BMJ Open. 2018 Mar 6;8(3):e020167. doi: 10.1136/bmjopen-2017-020167 (PMC5855206; doi:10.1136/bmjopen-2017-020167)
Supplement: Author's manuscript [file bmjopen-2017-020167.draft_revisions.pdf]

BMJ Open

BMJ Open is committed to open peer review. As part of this commitment we make the peer review history of every article we publish publicly available.

When an article is published we post the peer reviewers' comments and the authors' responses online. We also post the versions of the paper that were used during peer review. These are the versions that the peer review comments apply to.

The versions of the paper that follow are the versions that were submitted during the peer review process. They are not the versions of record or the final published versions. They should not be cited or distributed as the published version of this manuscript.

BMJ Open is an open access journal and the full, final, typeset and author-corrected version of record of the manuscript is available on our site with no access controls, subscription charges or pay-per-view fees (http://bmjopen.bmj.com).

If you have any questions on BMJ Open's open peer review process please email editorial.bmjopen@bmj.com

BMJ Open

Randomized trial of coconut oil, olive oil or butter on blood lipids and other cardiovascular risk factors in healthy men and women

Journal:	*BMJ Open*
Manuscript ID	bmjopen-2017-020167
Article Type:	Research
Date Submitted by the Author:	18-Oct-2017
Complete List of Authors:	Khaw, Kay-Tee; University of Cambridge, Department of Public Health and Primary Care<br>Sharp, Stephen; University of Cambridge, MRC Epidemiology Uniot<br>Finikarides, Leila; British Broadcasting Corporation<br>Afzal, Islam; Aston University, Aston Medical Research Institute<br>Lentjes, Marleen; University of Cambridge, Department of Public Health and Primary Care<br>Luben, Robert; University of Cambridge, Department of Public Health and Primary Care<br>Forouhi, Nita; University of Cambridge, MRC Epidemiology Unit
Keywords:	blood lipids, dietary fats, randomized trial, coconut oil, olive oil

SCHOLARONE™
Manuscripts

For peer review only

18 October 2017 V2

Randomized trial of coconut oil, olive oil or butter on blood lipids and other cardiovascular risk factors in healthy men and women

Kay-Tee Khaw[1], Stephen J. Sharp[2], Leila Finikarides[3], Islam Afzal[4], Marleen Lentjes[1], Robert Luben[1], and Nita G. Forouhi[2]

Authors' Affiliations:

Kay-Tee Khaw, MBBChir, FRCP, FMedSci, Professor

Marleen Lentjes, PhD, Research Nutritionist

Robert Luben, BSc, Bioinformatics

[1]Department of Public Health and Primary Care, University of Cambridge School of Clinical Medicine, United Kingdom;

Stephen J. Sharp MA, MSc, Senior Statistician

Nita G. Forouhi, MBBS, PhD, MRCP, FFPH, Professor

[2]Medical Research Council Epidemiology Unit, University of Cambridge School of Clinical Medicine, United Kingdom;

Leila Finikarides, MSc, producer

[3]BBC Television"Trust Me I'm a Doctor", BBC Glasgow, Glasgow, United Kingdom;

Islam Afzal, PhD, Research Facilitator

[4] Aston Medical Research Institute, Aston Medical School, Aston University, Birmingham, United Kingdom

Clinical trials registration: NCT03105947 Clinical Trials.gov USNIH

Short running title: **Coconut oil, butter or olive oil and blood lipids**

Word count: text (excluding abstract, tables & references) = 5575 abstract = 586

Number of tables and figures: 3 Tables, 5 Figures, 1 supplemental table

Key words: coconut oil, butter, olive oil, dietary fat, lipids, LDL-Cholesterol, randomized trial

Correspondence to:

Kay-Tee Khaw, Professor

Clinical Gerontology Unit Box 251, University of Cambridge School of Clinical Medicine, Addenbrooke's Hospital, Cambridge, CB3 0EQ, United Kingdom

e-mail: kk101@medschl.cam.ac.uk Phone: +44 1223 336927

For peer review only - http://bmjopen.bmj.com/site/about/guidelines.xhtml

18 October 2017 V2

Abstract

Importance: High dietary saturated fat intake is associated with higher blood concentrations of LDL-cholesterol, an established risk factor for coronary heart disease. However, there is increasing interest in whether various dietary oils or fats such as extra virgin coconut oil with different fatty acid profiles may have different metabolic effects but trials have reported inconsistent results.

Objective: To compare changes in blood lipid profile, weight, fat distribution, and metabolic markers after four weeks consumption of 50g daily of one of three different dietary fats: extra virgin coconut oil, butter, or extra virgin olive oil: in healthy men and women in the general population.

Design: Randomized clinical trial conducted over June and July 2017.

Setting: General community in Cambridgeshire, United Kingdom

Participants: Volunteer adults were recruited by the British Broadcasting Corporation (BBC) through their websites. Eligibility criteria were men and women aged 50-75 years, with no known history of cancer, cardiovascular disease or diabetes, not on lipid lowering medication, no contraindications to a high fat diet and willingness to be randomized to consume one of the three dietary fats for four weeks. Of 160 individuals initially expressing an interest and assessed for eligibility, 96 were randomized to one of three interventions; 2 individuals subsequently withdrew and 94 men and women attended a baseline assessment. Their mean age was 60 years, 67% were women, and 98% were European Caucasian. Of these, 91 men and women attended a follow up assessment four weeks later.

Intervention: Participants were randomized to extra virgin coconut oil, extra virgin olive oil, or unsalted butter and asked to consume 50g daily of one of these fats for four weeks, which they could incorporate into their usual diet or consume as a supplement.

Main Outcomes and Measures: The primary outcome was change in serum Low Density Lipoprotein cholesterol (LDL-C); secondary outcomes were change in total and high density lipoprotein cholesterol (TC and HDL-C), TC/HDL-C ratio, and non-HDL-C; change in weight, body mass index (BMI), waist circumference, percent body fat, systolic and diastolic blood pressure, fasting plasma glucose and C-Reactive Protein.

Results: LDL-C concentrations were significantly increased on butter compared to coconut oil (+0.42, 95% CI 0.19,0.65 mmol/L, P<0.0001), and to olive oil (+0.38, 95% CI 0.16,0.60 mmol/L, P<0.0001), with no differences in change of LDL-C in coconut oil compared to olive oil (-0.04,

For peer review only - http://bmjopen.bmj.com/site/about/guidelines.xhtml

95% CI -0.27, 0.19 mmol/L, P=0.74). Coconut oil significantly increased HDL-C compared to butter (+0.18, 95% CI 0.06,0.30 mmol/L) or olive oil (+0.16, 95% CI 0.03,0.28 mmol/L). Butter significantly increased TC/HDL-C ratio and non-HDL-C compared to coconut oil but coconut oil did not significantly differ from olive oil for TC/HDL-C and non-HDL-C. There were no significant differences in changes in weight, BMI, central adiposity, fasting blood glucose, systolic or diastolic blood pressure amongst any of the three intervention groups.

Conclusions and Relevance: Two different dietary fats (butter and coconut oil) which are predominantly saturated fats, appear to have different effects on blood lipids compared to olive oil, a predominantly monounsaturated fat. The effects of different dietary fats on lipid profiles, metabolic markers and health outcomes may vary not just according to the general classification of their main component fatty acids as saturated or unsaturated but possibly according to different profiles in individual fatty acids, processing methods, as well as the foods in which they are consumed or dietary patterns. These findings do not alter current dietary recommendations to reduce saturated fat intake in general but highlight the need for further elucidation of the more nuanced relationships between different dietary fats and health.

Clinical trials registration: NCT03105947 Clinical Trials.gov USNIH

For peer review only

Strength and limitations of the study

Strengths

Randomized trial comparing three dietary fat interventions

Good compliance

Objective measures of outcome: blood biochemistry and anthropometry

Participants from general community in "real life" setting

Limitations

Participants were not blinded as to the intervention

Relatively short term for four weeks

Intermediate endpoints of blood lipids and anthropometry not clinical events

For peer review only

For peer review only - http://bmjopen.bmj.com/site/about/guidelines.xhtml

Introduction

This trial was conducted in the context of debate over longstanding dietary recommendations to reduce dietary fat intake for health. The Women's Health Initiative reported no differences in cardiovascular disease in women randomized to low fat and usual diets over 8 years[1] while an intervention comparing a low fat diet with a Mediterranean diet with extra virgin olive oil, or nuts (PREDIMED) reported approximately 30% lower cardiovascular events in both Mediterranean diet arms after 4.8 years[2]; meta-analyses of observational studies and trials report inconsistent findings in the relationship between dietary saturated fatty acids and cardiovascular disease[3, 4]; and the relationships of dairy fats including milk and butter with cardiovascular disease also being debated[5-7]. Part of the debate relates to the increasing evidence that different individual fatty acids, such as the odd chain or even chain saturated fatty acids, or short, medium and long chain saturated fatty acids, may have different metabolic pathways and subsequent potential health effects, as well as the understanding that diet is more complex than individual nutrients or generic biochemical nutrient groups, and that contextual factors such as foods and dietary patterns are important. The 2015-2020 US dietary guidelines[8] now focus on foods and dietary patterns and while they recommend limiting saturated and trans fats, they no longer explicitly recommend limiting total fat. In this context therefore, there is renewed interest in the health effects of different fats and oils.

Extra virgin coconut oil has recently been promoted as a healthy oil. Though high in saturated fat, the main saturated fatty acid, lauric acid (c12:0), has been suggested to have different metabolic, and hence health effects compared to other saturated fatty acids such as palmitic acid (c16:0), predominant in butter, palm oil and animal fat. In particular, it has been suggested that coconut oil does not raise total cholesterol or LDL-Cholesterol as much as butter. A recent review on coconut oil and cardiovascular risk factors in humans concluded that the evidence of an association between coconut oil consumption and blood lipids or cardiovascular risk was mostly poor quality[9]. While some small studies have been reported comparing coconut oil and butter, these have been small[10, 11], and none conducted in the UK where overall dietary patterns are very different. The 2017 American Heart Association Presidential advisory on dietary fats and cardiovascular disease highlighted the paucity of evidence over the long term health effects of saturated fats such as coconut oil and reinforced strongly recommendations to lower dietary saturated fat and replacement with

For peer review only - http://bmjopen.bmj.com/site/about/guidelines.xhtml

For peer review only

unsaturated fat to lower LDL-cholesterol and prevent cardiovascular disease[12]. In particular, they stated “because coconut oil increases LDL-Cholesterol, a cause of cardiovascular disease, and has no known offsetting favourable effects, we advise against the use of coconut oil”[12].

Though the PREDIMED study reported lower cardiovascular disease events in those randomized to extra virgin olive oil or added nuts[2], this trial reported no overall effects on LDL-cholesterol or total cholesterol for those on olive oil compared to the low fat diet [13]; results consistent with a review of intervention trials of high phenolic olive oil[14].

We therefore aimed to examine whether in free living healthy men and women in the UK, we could observe differences in blood lipids after one month’s consumption of 50g daily of one of three different fats within the context of their usual diet. Although this was a short term trial that did not address cardiovascular disease events, blood lipids are a well established risk factor for coronary heart disease and the aim was to compare directly the effects of three different fats: extra virgin coconut oil, butter (both predominantly saturated fats) with extra virgin olive oil (monounsaturated fat) on blood lipid profiles and metabolic measures, in a pragmatic trial using amounts feasible in daily diets.

This study was conducted in collaboration with the BBC who filmed the trial for a future programme of “Trust me, I am a Doctor”.

For peer review only - http://bmjopen.bmj.com/site/about/guidelines.xhtml

Methods

Study population

Participants were volunteers living in the general community predominantly in the Cambridgeshire area, recruited through BBC advertising in May and June 2017. Eligible participants were men or women aged between 50-75 years who did not have a known medical history of heart disease, stroke, cancer, or diabetes, and who were not taking medication for lowering blood lipids such as statins. They had to be willing to be randomized to consume 50 g daily of one of the designated fats for four weeks, and not have any contraindications to eating a high fat diet such as gall bladder or bowel problems. Of 160 individuals expressing an interest, 96 were eligible and randomized to the intervention, 2 withdrew prior to the start of the study, and 94 attended a baseline assessment.

Allocation to Intervention

Participants were assigned a unique study identification number (ID). These ID numbers were randomized by computer generated allocation conducted by an independent statistician separately in men and women, into one of three parallel intervention arms approximately equal in size: extra virgin coconut oil, butter, or extra virgin olive oil.

Intervention

Participants attending the baseline assessment, at the end of their appointment, received one month's supply of one of the three different dietary fats to which they had been randomly allocated: extra virgin coconut oil, or butter or extra virgin olive oil. The BBC study organizer was given an ID list with the random allocation to the fats/oils and was responsible for giving each participant their supply of fat/oils. They were asked to eat 50g of these fats daily for four weeks and given measuring cups for the 50ml fat and oils: butter was prepacked in 20g and 30g portions. They were asked to continue with their usual diet, and either incorporate the fat or oil into their daily diet to substitute for other fats or oils, or they could eat these fats as a supplement. They also had information sheets with suggestions for how the fats could be consumed including recipes. The fats selected were standard products available from supermarkets bought from suppliers; organic extra virgin coconut oil, organic unfiltered extra virgin olive oil, and organic unsalted butter. Samples of the oils/fats used in the trial were sent to a reference laboratory: the West Yorkshire Analytic Services, a UKAS accredited testing service for food composition.

For peer review only - http://bmjopen.bmj.com/site/about/guidelines.xhtml

Assessments

Participants attended two assessments at a community centre in Cambridge: one at baseline before the start of the intervention in June 2017, and one at the end of four weeks in July 2017. Prior to their initial assessment, they were asked to fill in a short questionnaire about their health and lifestyle including physical activity and diet as well as complete an online 24 hour dietary assessment questionnaire with automated nutrient intake estimation, developed in Oxford, the DietWebQ[15]. All assessments were conducted between 0800 and 1230. Participants were all fasted for a minimum of 4 hours prior to attending the assessment; the majority were fasted overnight. They had height and waist circumference measured to a standardised protocol in light clothing without shoes and blood pressure measured using an automated OMRON device after being seated resting for 5 minutes. The mean of two readings for blood pressure, height and waist were used for analysis. Weight and percent body fat were measured using a Tanita body composition monitor. All measurements were conducted by two trained observers unaware of allocation to the oils/fats. Participants gave a 20 ml blood sample which was stored in a 4°C refrigerator then sent to the laboratory by courier for same day sample processing and storage for later analysis.

After four weeks at the end of the intervention, they attended again for a follow up assessment where the same measurements of height, waist circumference, blood pressure, weight and percent body fat were conducted, and another fasting 20 ml blood sample taken. Measurements were recorded on new forms and observers and participants did not have access to the measurements taken at the baseline visit. Just prior to this visit, participants were asked to fill in again the online 24 hour DietWebQ. Participants also filled in short questionnaire about their experiences on the intervention fats. This included a question about their overall experience of consuming the assigned oil/fat in the study where they were asked on average, over the past 4 weeks whether they felt mostly the same as usual, mostly felt better than usual or mostly felt worse than usual with an open ended section for comments including side effects, and overall compliance with consuming the fats which they were asked to self-rate between 0% to 100%. They were also asked whether they changed their type, level or frequency of physical activity in the past month since being in the study and had three options, no overall change in activity, increase in activity or decrease in activity.

For peer review only - http://bmjopen.bmj.com/site/about/guidelines.xhtml

Blood samples were identified only by a study ID number and were processed using standard protocols and assayed in two batches at the end of the baseline and follow up assessments in the Core Biochemical Assay Laboratory (CBAL) Cambridge University Hospitals which has UKAS Clinical Pathology Accreditation; blood samples from individuals on different interventions were thus all assayed in the same batch. The laboratory assays were conducted in a blinded fashion without any indication of the allocated intervention. Cholesterol (TC) and triglycerides were measured using enzymatic assays,[16, 17] high-density-lipoprotein cholesterol (HDL-C) was measured using a homogenous accelerator selective detergent assay automated on the Siemens Dimension RxL analyser, and low density lipoprotein cholesterol (LDL-C) was calculated from the triglyceride, HDL and cholesterol concentrations as described in the Friedewald formula (LDL = Cholesterol - HDL - (Triglycerides/2.2)[18]. Total to HDL-C ratio was computed, and non-HDL-C was computed as TC minus HDL-c.

Plasma glucose was measured using the hexokinase-glucose-6-phosphate dehydrogenase method and high sensitivity human C-Reactive Protein was assayed using an automated colourimetric immunoassay: Siemens Dimension® CCRP *Cardio*Phase® high sensitivity CRP.

Trial outcomes

The trial was registered in April 2017 with clinical trials registration: NCT03105947**.** The primary outcome of the trial was change in low density lipoprotein cholesterol (LDL-C) from baseline to follow up. Secondary outcomes were change in each of the following variables from baseline to follow up: total cholesterol (TC), high density lipoprotein cholesterol (HDL-C), triglycerides; ratio of total cholesterol/HDL-C, non-HDL cholesterol, fasting blood glucose, C-Reactive Protein, weight, body mass index(BMI), body fat %, waist circumference, systolic blood pressure and diastolic blood pressure..

Statistical analysis

The study aimed to recruit a total of 90 participants: 30 individuals per group would provide 80% power to detect a difference in mean within-person change in LDL cholesterol (baseline to follow-up) comparing pairs of randomized groups (butter vs coconut oil and butter vs olive oil) of approximately 0.45 mmol/L, assuming a standard deviation of LDL cholesterol of 1.04 mmol/L.[19] With 2 primary pairwise comparisons, the significance level for each comparison was set to 2.5%. For total cholesterol (a secondary outcome), 30 individuals per group would provide approximately 80% power to detect a difference between each pair of groups of 0.5

For peer review only - http://bmjopen.bmj.com/site/about/guidelines.xhtml

mmol/L assuming a standard deviation of cholesterol of 1.17mmol/L[19]. Because the randomized groups were compared using analysis of covariance (i.e. adjusting for the baseline value of the outcome), for both LDL and total cholesterol, a correlation between baseline and follow up values was incorporated into the calculation, using the method described by Borm et al.[20, 21] A value of 0.79 was used for this correlation based on data from a population study[19]

This magnitude of difference was what can be estimated from metabolic ward studies in which replacement of 10% dietary calories from saturated fat is associated with 0.52 mmol/L cholesterol difference[22] though this did not specify the food sources of saturated fats, and a small intervention trial (n=28) comparing butter and coconut oil with sunflower oil[10].

Baseline characteristics were summarised separately for each randomized group. As recommended by CONSORT, no p-values were calculated for this table. The primary analysis used an Intention To Treat (ITT) population, which included all individuals in the group to which they were randomized, regardless of the extent to which they adhered to the intervention. A secondary analysis used a Per Protocol (PP) population. This was a subset of the ITT population consisting of those individuals who adhered to the intervention. Participants who reported >75% adherence when asked at the follow up visit were included in the PP population.

For each outcome, a p-value was calculated to compare the 3 randomized groups using a linear regression model, in which change from baseline was the outcome, and including a dummy variable for randomized group and the baseline value of the outcome variable as covariates, i.e. an Analysis of Covariance (ANCOVA) model. Differences between each pair of randomized groups and 95% confidence intervals (CIs) were also estimated from a similar model.

Ethics

Ethics approval was given for the study by the University of Cambridge Human Biology Research Ethics committee HBREC 2017.05.

For peer review only - http://bmjopen.bmj.com/site/about/guidelines.xhtml

Results

Figure 1 is the CONSORT diagram for the trial. The recruitment was conducted by the BBC coordinator through BBC website advertising. From 160 individuals initially expressing an interest, and after exclusion criteria, 96 individuals were randomized and invited to a baseline assessment session in June 2017. Two individuals subsequently withdrew and 94 individuals attended the baseline assessment session in June 2017. At the four week follow up assessment in July 2017, 91 individuals attended; 3 individuals did not attend follow up indicating personal circumstances.

Table 1 shows descriptive characteristics for the participants at the baseline assessment according to the allocation to dietary oils/fats. Two thirds of the participants were women and the mean age overall was 60 years.

Table 2 shows mean changes in the primary and secondary outcomes at the four week follow up within each randomized group, and comparisons between each pair of randomized groups. LDL-C concentrations were significantly increased on butter compared to coconut oil (+0.42, 95% CI 0.19,0.65 mmol/L, P<0.0001), and olive oil (+0.38L, 95% CI 0.16,0.60 mmol/L, P<0.0001), with no differences in change of LDL-C in coconut oil compared with olive oil (-0.04, 95% CI -0.27, 0.19 mmol/L, P=0.74). Coconut oil significantly increased HDL-C compared to butter (+0.18, 95% CI 0.06,0.30 mmol/L) or olive oil (+0.16, 95% CI 0.03,0.28 mmol/L).

Butter significantly increased the cholesterol/HDL-C ratio compared to coconut oil (+0.36, 95%CI 0.18,0.54) and olive oil (+0.22,95% CI 0.04,0.40) and also increased non-HDL-C compared to coconut oil (+0.39, 95% CI 0.16,0.62 mmol/L) and olive oil (+0.39(95% CI 0.16,0.62) but coconut oil did not significantly differ from olive oil for change in cholesterol/HDL-C ratio (-0.14, 95%CI -0.33,0.05) or non-HDL-C (0.00, 95% CI -0.23,0.24 mmol/L).

Coconut oil also significantly lowered C-Reactive Protein in comparison with olive oil (-0.59, 95% CI -1.14,-0.05 mg/L) but not compared to butter. There were no significant differences in changes in weight, BMI, central adiposity, fasting blood glucose, systolic or diastolic blood pressure amongst any of the three intervention groups. For weight, for example, the

For peer review only - http://bmjopen.bmj.com/site/about/guidelines.xhtml

estimated mean (95% CI) changes in weight were +0.27(95% CI -0.03,0.57)kg, 0.04 (95% CI -0.31,0.39)kg and -0.04(95% CI -0.35,0.27) kg for coconut oil, butter and olive oil respectively.

Figure 2 shows the difference in the primary outcome (LDL-C) between each pair of randomized groups in the 91 individuals who attended baseline and follow up. **Figures 3, 4, and 5** show the differences in secondary outcomes comparing butter versus coconut oil, coconut oil versus olive oil, and butter versus olive oil respectively. For comparability the differences are reported in units of baseline standard deviation (SD) for the different outcomes in Figures 3 to 5.

Self reported compliance was high: 87% of participants reported more than 75% compliance with the intervention over the 4 weeks which was similar among the groups (86% coconut oil, 88% butter and 85% olive oil). Secondary analyses on the 82 participants reporting more than 75% compliance showed similar results (not shown). Reported experience consuming the fats was similar between groups: 57%, 66%, and 60% reported feeling no different, 18%, 6% and 13% reported feeling better, and 25%, 27% and 23% reported feeling worse in the coconut oil, butter and olive oil groups respectively. Comparison of dietary intake using the 24 hour DietWebQ showed similar levels of dietary intake across intervention groups at baseline. Following the intervention, total fat intake increased in all intervention groups but estimates for absolute intakes of carbohydrate, protein and alcohol did not differ between intervention groups (Table 3). Most of the participants reported no changes in usual physical activity (79%, 73% and 89% no change; 14%, 15% and 4% increased usual physical activity and 7%, 12% and 7% decreased usual physical activity in the coconut oil, butter and olive oil groups respectively). In a post hoc exploratory analysis, exclusion of individuals who reported increasing usual physical activity had little effect on significant differences between interventions for LDL-C and HDL-C and did not alter the findings for weight change (supplementary table 4).

Supplementary appendix 1 shows the fatty acid composition of the three oils/fats used in the intervention. Coconut oil was 94 % saturated fatty acids, of which the main components were lauric acid C12:0 (48%), myristic acid C14:0 (19%), palmitic acid C16:0 (9%) and caprylic acid C8:0 (9%); and

For peer review only - http://bmjopen.bmj.com/site/about/guidelines.xhtml

18 October 2017 V2

5% mono unsaturated fat, mainly oleic acid C18:1n9 (5%). Butter was 66% saturated fatty acids, of which the main components were palmitic acid C16:0 (28%), stearic acid C18:0 (12%), myristic acid C14:0 (11%); 26% monounsaturated fat, mainly oleic acid C18:1n9 (22%); and 3% polyunsaturated fat, linoleic acid C18:2n6 (2%) and alpha-linolenic acid (1%). Olive oil was 19% saturated fatty acids, mainly palmitic acid C16:0, 15% with stearic acid C18:0 (3%); 68% monounsaturates with the main component being oleic acid C18:1n9 (64%); and 13% polyunsaturates Linoleic acid C18:2n6 (12%). These profiles are very similar to those reported from other studies[9].

For peer review only

For peer review only - http://bmjopen.bmj.com/site/about/guidelines.xhtml

Discussion

In this trial, middle aged men and women living in the general community were randomly allocated to consume 50g extra virgin coconut oil, or 50g butter, or 50g extra virgin olive oil for four weeks. We observed at the end of the trial significantly different changes in LDL-C and HDL-C concentrations between the three intervention groups; in pairwise comparisons, coconut oil did not significantly raise LDL-C concentrations compared to olive oil while butter significantly raised LDL-C concentrations compared to both coconut oil and olive oil. Coconut oil significantly raised HDL-C concentrations compared to both butter and olive oil. Butter also significantly raised cholesterol/HDL-C ratio and non-HDL-Cholesterol more than both coconut oil and olive oil but there were no differences between coconut oil and olive oil for changes in cholesterol/HDL-C and non-HDL-C cholesterol.

There were no significant differences in weight or BMI change, change in central adiposity as measured by waist circumference or percent body fat. There were also no significant differences in change in fasting glucose, or systolic and diastolic blood pressure among the three different fat interventions. In pairwise comparison, coconut oil significantly lowered C-Reactive Protein compared to olive oil but there were no significant differences between coconut oil and butter for C-Reactive Protein.

The results were somewhat surprising for a number of reasons. Coconut oil is predominantly (approximately 90%) saturated fat which is generally held to have an adverse effect on blood lipids by increasing blood LDL-C concentrations. However, the saturated fatty acid profiles of different dietary fats vary substantially; coconut oil is predominantly (around 48%) lauric acid (12:0) compared to butter (66% saturated fat) which is about 40% palmitic (16:0) and stearic (18:0) acids and leading to suggestions that coconut oil may not have the same health effects as other foods high in saturated fat[9]. Nevertheless, though reviews on coconut oil and cardiovascular disease risk factors have concluded that the evidence of an association between coconut oil consumption and blood lipids or cardiovascular risk was mostly poor quality[9], trials have generally reported that coconut oil consumption raises LDL-C in comparison to polyunsaturated oil such as safflower oil, though not as much in comparison to butter[10, 11].

For peer review only - http://bmjopen.bmj.com/site/about/guidelines.xhtml

Based on 3 randomized crossover trials of good scientific quality, one trial reported butter increased LDL-C more than coconut oil which raised LDL-C more compared to safflower oil[10]; a second that coconut oil raised LDL-C more than beef fat which raised LDL-C more than safflower oil[23], and a third reported that coconut oil raised LDL-C more than palm oil which raised LDL-C more than olive oil[24]. The current study observed that butter raised LDL-C more than coconut oil but that coconut oil did not differ from olive oil. Two studies showed higher HDL-C with coconut oil compared with other fats whether beef fat, safflower oil or olive oil[23, 24]. Thus far, the current results are consistent with previous studies indicating that butter raises LDL-C more than coconut oil, and also that coconut oil also raises HDL-C. However, the present study is an exception in not finding any increase in LDL-C compared to an unsaturated oil, in this case, olive oil.

This is the largest trial reported to date on coconut oil and lipids apart from a recent study of 200 individuals with established coronary heart disease comparing coconut oil with sunflower oil over 2 years that reported no differences in blood lipids but virtually all the participants were on statin therapy[25] which makes findings difficult to interpret.

Direct comparisons between studies are problematic because of different oils used; we used extra virgin olive oil as a comparison group rather than a polyunsaturated oil such as safflower or sunflower oil, essentially for feasibility reasons of likely participant compliance with the requirement for 50g intake daily. The PREDIMED study reported no significant difference in change in LDL–C or total cholesterol but significant lowering of the cholesterol/HDL-C ratio in the Mediterranean diet supplemented with extra virgin olive oil compared to a low fat diet[2, 13]. A recent review reported that high phenolic olive oil does not modify the lipid profile compared to its low phenolic counterpart[14] though other studies have reported that extra virgin olive oil decreases LDL-C directly measured as concentrations of apoB-100 and the total number of LDL particles as assessed by NMR spectroscopy[26, 27]. We therefore expected coconut oil would raise LDL-C compared to olive oil, but in the current study we observed no evidence of an overall average increase in LDL-C in individuals allocated either to the coconut oil or olive oil intervention.

For peer review only - http://bmjopen.bmj.com/site/about/guidelines.xhtml

Lack of compliance with consuming the dietary fat would lead to no differences between groups and hence explain the lack of differences in LDL-C between coconut oil and olive oil groups. However, in this group of volunteers, reported compliance was high and did not differ between groups; in addition, those in the coconut oil group had significantly greater increases in HDL-C compared to those allocated to olive oil or butter so lack of compliance is unlikely to be an explanation.

The predominant fatty acid in coconut oil, lauric acid (C12:0) as well as myristic acid (C14:0) are medium chain fatty acids that are rapidly absorbed, taken up by the liver and oxidized to increase energy expenditure which is a possible explanation for why coconut oil may have different effects compared to other saturated fats[28]. It is also possible that differences could be attributed to the use of extra virgin preparations of coconut oil rather than standard coconut oil; different methods of preparation such as the chilling method for virgin coconut oil compared to refined, bleached and deodorized coconut oil may influence phenolic compounds and antioxidant activity[29] thus, processing of oils changes their composition, biological properties and consequent potential metabolic effects. The variations in possible health effects resulting from variations in processing of different fats is well documented in the large literature on hydrogenation of polyunsaturated oils to make solid margarines which may increase harmful trans- fats[30]. In this context it is notable that the major trial (PREDIMED) reporting reduction in cardiovascular risk with a Mediterranean diet used extra virgin olive oil[2], while other studies which reported null findings with olive oil may not have always specified the product used[14].

There was no evidence of difference between groups in mean weight, BMI, percent body fat, or central adiposity at the end of this trial; however, these were secondary endpoints for which the trial was not specifically powered. Nevertheless the estimated 95% CI around mean weight differences at the end for the trial were not large. The participants were asked to consume 50g of fat or oils daily. They could do this in the context of their usual diet by substituting for their usual fats, or by consuming these as a supplement. In practice, most participants reported finding it difficult to substitute the different fats or oils for cooking in their usual diet and usually consumed these as a supplement. These fats if taken in addition to their usual diet would have been approximately 450 additional calories daily, which if

For peer review only - http://bmjopen.bmj.com/site/about/guidelines.xhtml

18 October 2017 V2

consistently taken four weeks might be expected to be nearly 13,000 additional calories resulting in likely weight gain of 1 to 2kg. This information was provided in the information sheet with the informed consent for participants. While it is possible that participants may have consciously changed behaviours to maintain body weight such as reducing their other dietary intake because of the additional fat or being more physically active, many participants reported that the high fat diet resulted in feeling full and eating less.

It is also possible that even though this was a randomized trial, in an unblinded study, participants may have changed behaviours differentially in the different intervention groups resulting in differences in lipids or lack of differences in weight observed rather than being attributed to the dietary fat interventions. The majority of the participants reported no change in usual physical activity though slightly more participants in the coconut oil and butter groups reported increasing usual physical activity (14% and 15% respectively) compared to 4% in the olive oil group. Nevertheless exclusion of all individuals reporting increased usual physical activity from the analyses did not change the findings. Dietary factors apart from fat most likely to influence HDL-C, total alcohol intake or change in alcohol intake, did not differ significantly between intervention groups and in fact alcohol intake decreased slightly during the trial which would not explain any increases in HDL-C observed. There is therefore no evidence to suggest that differences in lipids, or lack of differences in weight change were likely to be attributed to differential changes in behaviour

The main strengths of this study are the randomized design with high completion rate (91/94 individuals returned to follow up) and self reported dietary compliance (nearly 90% participants with over 75% adherence) over four weeks. This is also larger than most trial reported with the exception of the trial in India in individuals with heart disease most of whom were taking statins[25]. The current trial by contrast, was conducted in individuals in the general population.

This trial has limitations. It was a short term trial of four weeks intervention so we are unable to know what would have happened if the intervention had continued for a longer period. Moreover, the current findings only apply to the intermediate metabolic (lipid) risk markers and cannot be extended to findings for clinical endpoints.

For peer review only - http://bmjopen.bmj.com/site/about/guidelines.xhtml

It was designed as a pragmatic trial in free living individuals rather than a controlled metabolic ward trial such that individuals were asked only to consume the 50g of allocated fat or oil daily. While they had suggestions as to how this could be done, such as incorporating in recipes, substituting the fat for their usual dietary oil or fat, or simply consuming this as a supplement, we made no attempt to control other aspects of their usual diet in particular, total energy intake. Individuals may have changed their behaviours in different ways to accommodate this additional fat, whether by modifying other aspects of their diet for instance, increasing foods such as bread and potatoes or salads to eat with the fats, or consciously reducing other food intake or changing physical activity patterns to control energy balance. Nevertheless, this trial is more reflective of real life situations.

While self reported compliance was high, this was subjective and we did not measure the blood fatty acid profile in participants following the intervention for an objective biomarker of compliance. Nevertheless, we did observe differential changes in blood lipids during the intervention.

We did not have a non–additional fat intervention as a comparison group, nor a comparison group with polyunsaturated oils. This was for reasons of feasibility and practicality as it would have added substantially to the numbers (another 30 for an additional intervention arm) and we were also uncertain as to compliance with consumption of 50g of polyunsaturated oil daily in volunteers. We therefore used extra virgin olive oil as a comparison group as that has been generally reported in trials not to increase LDL-C. While the dose of saturated fat of 50g daily was substantial enough to raise LDL-C by levels estimated from previous metabolic ward studies, it was within a feasible daily consumption range.

The generalisability of the findings to the wider population is also unclear. The volunteers were clearly highly selected to be willing to participate in such a study, and also likely to be healthier than the general population, as for ethical reasons we excluded those with known prevalent cardiovascular disease, cancer or diabetes and also those on any lipid lowering medication or other contraindications to a high fat diet. Nevertheless, it is unlikely that the

For peer review only - http://bmjopen.bmj.com/site/about/guidelines.xhtml

effect of these dietary fats in this group of individuals recruited from the general population would be biologically different from the general population.

Implications

We focussed on LDL-Cholesterol for the primary endpoint as the causal relationship between LDL-C concentrations and coronary heart disease risk is well established, with about a 15% increase in coronary heart disease risk per 1 mmol/L increase in LDL-C concentrations, and reduction of LDL-C cholesterol lowers coronary heart disease risk[31]. Increase in LDL-C concentrations has been the main mechanism through which dietary saturated fat is believed to increase heart disease risk, though other pathways have been postulated. However, it is notable that some Mediterranean diet interventions such as the Lyon heart study (alpha linolenic acid)[32] or PREDIMED (extra virgin olive oil) [2] which have been reported to reduce cardiovascular risk in secondary and primary prevention may have effects through other pathways such as inflammation or endothelial function[33, 34]. Whatever the mechanisms, the evidence from prospective studies is consistent and strong that substitution of saturated fats by unsaturated fats is beneficial for cardiovascular risk[35].

In this trial the difference of 0.33mmol/L in LDL-C on butter compared to olive oil is consistent with previous studies[36]. We observed no differences in LDL-C on coconut oil compared to olive oil in this short term study. We also observed no differences among the various fats for a limited range of cardiovascular disease risk factors including fasting glucose, blood pressure and anthropometric measures.

The results of this study indicate that two different dietary fats (coconut oil and butter)which are predominantly saturated fats, appear to have different effects on blood lipids compared to olive oil, a predominantly monounsaturated fat. The effects of different dietary fats on lipid profiles, metabolic markers and health outcomes may vary not just according to the general classification of their main component fatty acids as saturated or unsaturated but possibly according to different profiles in individual fatty acids, processing methods, as well as the foods in which they are consumed or dietary patterns. There is increasing evidence that associations of saturated fatty acids with health outcomes may vary according to whether

For peer review only - http://bmjopen.bmj.com/site/about/guidelines.xhtml

they are odd or even chain saturated fatty acids, or their chain length[37-39]. Indeed, while overall the evidence indicates the substitution of dietary saturated fats with polyunsaturated fats is beneficial for coronary heart disease risk[40] heterogeneity in findings from observational studies and trials may reflect different dietary sources of fats[4, 41] As the summary from Joint FAO/WHO 2008 Expert Consultation on Fats and Fatty Acids in Human Nutrition comments: *"There are inherent limitations with the convention of grouping fatty acids based only on number of double bonds….major groups of fatty acids are associated with different health effects…..individual fatty acids within each broad classification may have unique biological properties or effects…. Intakes of individual fatty acids differ across world depending on predominant food sources of total fats and oils."* The associations with health endpoints may well vary depending on the food sources.

The current short-term trial on an intermediate cardiovascular disease risk factor, LDL-C, does not provide evidence to modify existing prudent recommendations to reduce saturated fat in the diet as emphasized in most consensus recommendations[8, 12] and dietary guidelines should be based on a range of criteria[42]. However, the findings highlight the need for further elucidation of the more nuanced relationships between different dietary fats and health. There is increasing evidence that to understand the relationship between diet and health, we need to go beyond simplistic associations between individual nutrients and health outcomes and examine foods and dietary patterns as a whole. In particular, present day diets with high intakes of processed foods now incorporate many fats and oils such as soya bean oil, palm oil and coconut oil which have not been previously widely used in Western societies and not well studied. The relationships between different dietary fats, particularly some of the now more commonly used fats, and health endpoints such as cardiovascular disease events need to be better established.

For peer review only - http://bmjopen.bmj.com/site/about/guidelines.xhtml

Funding statement

This work was supported by the British Broadcasting Corporation, a National Institute of Health Research Senior Investigator Award to KTK and core MRC Epidemiology support (MC UU 12015/5).

Acknowledgements

This study was conducted in collaboration with the British Broadcasting Corporation (BBC) which provided support for the recruitment of participants, running of the community assessment clinic, and biochemistry measurements for lipids. Other costs were supported by the University of Cambridge through a National Institute of Health Research Senior Investigator Award to Kay-Tee Khaw. Nita G Forouhi acknowledge core MRC Epidemiology Support (MC UU 12015/5). We thank Keith Burling and Peter Barker from the Core Biochemical Assay Laboratory, CBAL in Cambridge for the laboratory assays, Shrikant Bangdiwala, University of North Carolina for conducting the computer generated random allocation of participants to the interventions, Timothy Key and colleagues at Oxford University for the use of the DietWebQ, and Nichola Dalzell and Shabina Hayat, Department of Public Health and Primary Care, and Eirini Trichia, Richard Powell and Merial Smith,MRC Epidemiology Unit, University of Cambridge for logistical support. We thank the Cambridge Yoga Centre which hosted the assessment sessions for participants in June and July 2017. Most of all, we thank the participants from the general community who generously volunteered to take part in this trial; this study would not have been possible without their efforts and we are most grateful to them.

The BBC and the University of Cambridge collaborated in the design and conduct of the study, data collection and management of the study. The University of Cambridge investigators were solely responsible for the analysis and interpretation of the data, and preparation of the manuscript. The BBC producer coordinating the study (LF) is a co author who has reviewed and approved the manuscript but the BBC has otherwise had no editorial role in the manuscript.

For peer review only - http://bmjopen.bmj.com/site/about/guidelines.xhtml

Competing interest statement

All authors have completed the Unified Competing Interest form and declare no support from any organisation for the submitted work except as listed in the acknowledgements;; no financial relationships with any organisations that might have an interest in the submitted work in the previous three years and, no other relationships or activities that could appear to have influenced the submitted work

Conflicts of interest

None

Copyright

The Corresponding Author has the right to grant on behalf of all authors and does grant on behalf of all authors, a worldwide licence to the Publishers and its licensees in perpetuity, in all forms, formats and media (whether known now or created in the future), to i) publish, reproduce, distribute, display and store the Contribution, ii) translate the Contribution into other languages, create adaptations, reprints, include within collections and create summaries, extracts and/or, abstracts of the Contribution, iii) create any other derivative work(s) based on the Contribution, iv) to exploit all subsidiary rights in the Contribution, v) the inclusion of electronic links from the Contribution to third party material where-ever it may be located; and, vi) licence any third party to do any or all of the above

Ethics and Consent

Ethics approval was given by the University of Cambridge Human Biology Research Ethics Committee Application no. HBREC.2017.05. All participants gave signed informed consent. Clinical Trials registration April 2017 NCT03105947 USNIH Clinical Trials.gov

Contributors and transparency declaration

Kay-Tee Khaw had full access to all of the data in the study and takes responsibility for the integrity of the data and the accuracy of the data analysis. The lead author and guarantor Khaw affirms that the manuscript is an honest, accurate, and transparent account of the study being reported; that no important aspects of the study have been omitted; and that any discrepancies from the study as planned have been explained.

For peer review only - http://bmjopen.bmj.com/site/about/guidelines.xhtml

For peer review only

Study concept and design: Khaw, Forouhi, Finikarides

Acquisition of data: Khaw, Forouhi, Finikarides, Afzal, Luben, Lentjes

Analysis and interpretation of the data: Sharp, Khaw, Forouhi

Drafting of the manuscript: Khaw

Critical revision of the manuscript for important intellectual content: Forouhi, Sharp, Afzal, Finkarides, Luben, Lentjes

Obtaining funding: Khaw, Finikarides, Forouhi

Administrative, technical or material support: Khaw, Forouhi, Finikarides, Afzal, Luben, Sharp, Lentjes

Data sharing statement

Data are available. Please contact corresponding author.

Patient and public involvement

Design of the study: the BBC originally proposed the idea of a study to examine claims about the health benefits of coconut oil in response to public interest; the study would be part of their "Trust me, I'm a doctor" series

Involvement of lay people in the design of the study: This study was designed as a randomized trial in discussion with the BBC

The main outcome measures: objective measures of lipid profile, anthropometric measures, blood glucose and CRP was informed both by the medical literature and also by some popular beliefs about effects of coconut oil on these measures. We also asked about participants' subjective experience of their health on the different oils/fat though these were not specified as primary or secondary outcome measures.

Recruitment: Recruitment to the study of volunteers from the general community was conducted by the BBC through their websites.

Dissemination of results to study participants: All study participants were invited to a feedback session on 14 August when they were presented the overall results of the study by the study investigators and had the opportunity to ask questions. They were also given their individual results on the study. They will be sent a copy of the study report when this is published. Participants have been thanked in the acknowledgements.

For peer review only - http://bmjopen.bmj.com/site/about/guidelines.xhtml

For peer review only

What is known

High dietary saturated fat intake has generally been associated with higher blood concentrations of LDL-cholesterol, an established risk factor for coronary heart disease. However, there is increasing interest in whether various dietary oils or fats such as extra virgin coconut oil with different fatty acid profiles may have different metabolic effects but trials have reported inconsistent results.

What this study adds

In a randomized trial in 91 apparently healthy community dwelling men and women aged 40-75 years in Cambridgeshire, United Kingdom, comparing extra virgin coconut oil, unsalted butter, or extra virgin olive oil 50g daily for four months, change in LDL-Cholesterol concentrations did not significantly differ in those randomized to coconut oil compared to olive oil, but were significantly higher in those randomized to butter compared to coconut oil or olive oil. The mean increase in HDL-cholesterol concentrations was significantly greater in coconut oil compared to either butter or olive oil. There were no significant differences in changes in weight, central adiposity, fasting blood glucose, or systolic or diastolic blood pressure between any of the fats.

This study observed heterogeneity in the relationship between different saturated fats and blood lipids and highlights the need for more research on the role of different fatty acids, as well as health effects of foods not just individual nutrients.

For peer review only

Reference List

1. Howard BV, Van HL, Hsia J et al. Low-fat dietary pattern and risk of cardiovascular disease: the Women's Health Initiative Randomized Controlled Dietary Modification Trial. JAMA 2006;295(6):655-666.

2. Estruch R, Ros E, Salas-Salvado J et al. Primary prevention of cardiovascular disease with a Mediterranean diet. N Engl J Med 2013;368(14):1279-1290.

3. Chowdhury R, Warnakula S, Kunutsor S et al. Association of dietary, circulating, and supplement fatty acids with coronary risk: a systematic review and meta-analysis. Ann Intern Med 2014;160(6):398-406.

4. Hooper L, Martin N, Abdelhamid A, Davey SG. Reduction in saturated fat intake for cardiovascular disease. Cochrane Database Syst Rev 2015;(6):CD011737.

5. Alexander DD, Bylsma LC, Vargas AJ et al. Dairy consumption and CVD: a systematic review and meta-analysis. Br J Nutr 2016;115(4):737-750.

6. Liang J, Zhou Q, Kwame AW, Su Y, Zhang Z. Biomarkers of dairy fat intake and risk of cardiovascular disease: a systematic review and meta analysis of prospective studies. Crit Rev Food Sci Nutr 2016;0.

7. Pimpin L, Wu JH, Haskelberg H, Del GL, Mozaffarian D. Is Butter Back? A Systematic Review and Meta-Analysis of Butter Consumption and Risk of Cardiovascular Disease, Diabetes, and Total Mortality. PLoS One 2016;11(6):e0158118.

8. Department of Health and Human Services U, Department of Agriculture (US). 2015-2020 Dietary Guidelines for Americans. 8th Edition. 2015

9. Eyres L, Eyres MF, Chisholm A, Brown RC. Coconut oil consumption and cardiovascular risk factors in humans. Nutr Rev 2016;74(4):267-280.

10. Cox C, Mann J, Sutherland W, Chisholm A, Skeaff M. Effects of coconut oil, butter, and safflower oil on lipids and lipoproteins in persons with moderately elevated cholesterol levels. J Lipid Res 1995;36(8):1787-1795.

11. Cox C, Sutherland W, Mann J, de JS, Chisholm A, Skeaff M. Effects of dietary coconut oil, butter and safflower oil on plasma lipids, lipoproteins and lathosterol levels. Eur J Clin Nutr 1998;52(9):650-654.

12. Sacks FM, Lichtenstein AH, Wu JHY et al. Dietary Fats and Cardiovascular Disease: A Presidential Advisory From the American Heart Association. Circulation 2017;136(3):e1-e23.

13. Estruch R, Martinez-Gonzalez MA, Corella D et al. Effects of a Mediterranean-style diet on cardiovascular risk factors: a randomized trial. Ann Intern Med 2006;145(1):1-11.

14. Hohmann CD, Cramer H, Michalsen A et al. Effects of high phenolic olive oil on cardiovascular risk factors: A systematic review and meta-analysis. Phytomedicine 2015;22(6):631-640.

For peer review only - http://bmjopen.bmj.com/site/about/guidelines.xhtml

15. Liu B, Young H, Crowe FL et al. Development and evaluation of the Oxford WebQ, a low-cost, web-based method for assessment of previous 24 h dietary intakes in large-scale prospective studies. Public Health Nutr 2011;14(11):1998-2005.

16. HAGEN JH, HAGEN PB. An enzymic method for the estimation of glycerol in blood and its use to determine the effect of noradrenaline on the concentration of glycerol in blood. Can J Biochem Physiol 1962;40:1129-1139.

17. Rautela GS, Liedtke RJ. Automated enzymic measurement of total cholesterol in serum. Clin Chem 1978;24(1):108-114.

18. Nauck M, Warnick GR, Rifai N. Methods for measurement of LDL-cholesterol: a critical assessment of direct measurement by homogeneous assays versus calculation. Clin Chem 2002;48(2):236-254.

19. Canoy D, Wareham N, Luben R et al. Serum lipid concentration in relation to anthropometric indices of central and peripheral fat distribution in 20,021 British men and women: results from the EPIC-Norfolk population-based cohort study. Atherosclerosis 2006;189(2):420-427.

20. Borm GF, Fransen J, Lemmens WA. A simple sample size formula for analysis of covariance in randomized clinical trials. J Clin Epidemiol 2007;60(12):1234-1238.

21. Forouhi NG, Menon RK, Sharp SJ et al. Effects of vitamin D2 or D3 supplementation on glycaemic control and cardiometabolic risk among people at risk of type 2 diabetes: results of a randomized double-blind placebo-controlled trial. Diabetes Obes Metab 2016;18(4):392-400.

22. Clarke R, Frost C, Collins R, Appleby P, Peto R. Dietary lipids and blood cholesterol: quantitative meta-analysis of metabolic ward studies. BMJ 1997;314(7074):112-117.

23. Reiser R, Probstfield JL, Silvers A et al. Plasma lipid and lipoprotein response of humans to beef fat, coconut oil and safflower oil. Am J Clin Nutr 1985;42(2):190-197.

24. Voon PT, Ng TK, Lee VK, Nesaretnam K. Diets high in palmitic acid (16:0), lauric and myristic acids (12:0 + 14:0), or oleic acid (18:1) do not alter postprandial or fasting plasma homocysteine and inflammatory markers in healthy Malaysian adults. Am J Clin Nutr 2011;94(6):1451-1457.

25. Vijayakumar M, Vasudevan DM, Sundaram KR et al. A randomized study of coconut oil versus sunflower oil on cardiovascular risk factors in patients with stable coronary heart disease. Indian Heart J 2016;68(4):498-506.

26. Hernaez A, Fernandez-Castillejo S, Farras M et al. Olive oil polyphenols enhance high-density lipoprotein function in humans: a randomized controlled trial. Arterioscler Thromb Vasc Biol 2014;34(9):2115-2119.

27. Hernaez A, Remaley AT, Farras M et al. Olive Oil Polyphenols Decrease LDL Concentrations and LDL Atherogenicity in Men in a Randomized Controlled Trial. J Nutr 2015;145(8):1692-1697.

28. DeLany JP, Windhauser MM, Champagne CM, Bray GA. Differential oxidation of individual dietary fatty acids in humans. Am J Clin Nutr 2000;72(4):905-911.

29. Marina AM, Man YB, Nazimah SA, Amin I. Antioxidant capacity and phenolic acids of virgin coconut oil. Int J Food Sci Nutr 2009;60 Suppl 2:114-123.

For peer review only

For peer review only - http://bmjopen.bmj.com/site/about/guidelines.xhtml

30. Kummerow FA. The negative effects of hydrogenated trans fats and what to do about them. Atherosclerosis 2009;205(2):458-465.

31. Goodman DS, Hulley SB, Clark LT. Report of the national cholesterol education program expert panel on detection, evaluation, and treatment of high blood cholesterol in adults. Archives of Internal Medicine 1988;148(1):36-69.

32. de LM, Renaud S, Mamelle N et al. Mediterranean alpha-linolenic acid-rich diet in secondary prevention of coronary heart disease. Lancet 1994;343(8911):1454-1459.

33. Casas R, Sacanella E, Urpi-Sarda M et al. The effects of the mediterranean diet on biomarkers of vascular wall inflammation and plaque vulnerability in subjects with high risk for cardiovascular disease. A randomized trial. PLoS One 2014;9(6):e100084.

34. Casas R, Sacanella E, Urpi-Sarda M et al. Long-Term Immunomodulatory Effects of a Mediterranean Diet in Adults at High Risk of Cardiovascular Disease in the PREvencion con DIeta MEDiterranea (PREDIMED) Randomized Controlled Trial. J Nutr 2016;146(9):1684-1693.

35. Li Y, Hruby A, Bernstein AM et al. Saturated Fats Compared With Unsaturated Fats and Sources of Carbohydrates in Relation to Risk of Coronary Heart Disease: A Prospective Cohort Study. J Am Coll Cardiol 2015;66(14):1538-1548.

36. Engel S, Tholstrup T. Butter increased total and LDL cholesterol compared with olive oil but resulted in higher HDL cholesterol compared with a habitual diet. Am J Clin Nutr 2015;102(2):309-315.

37. Khaw KT, Friesen MD, Riboli E, Luben R, Wareham N. Plasma phospholipid fatty acid concentration and incident coronary heart disease in men and women: the EPIC-Norfolk prospective study. PLoS Med 2012;9(7):e1001255.

38. Praagman J, Beulens JW, Alssema M et al. The association between dietary saturated fatty acids and ischemic heart disease depends on the type and source of fatty acid in the European Prospective Investigation into Cancer and Nutrition-Netherlands cohort. Am J Clin Nutr 2016;103(2):356-365.

39. Forouhi NG, Koulman A, Sharp SJ et al. Differences in the prospective association between individual plasma phospholipid saturated fatty acids and incident type 2 diabetes: the EPIC-InterAct case-cohort study. Lancet Diabetes Endocrinol 2014;2(10):810-818.

40. Mozaffarian D, Micha R, Wallace S. Effects on coronary heart disease of increasing polyunsaturated fat in place of saturated fat: a systematic review and meta-analysis of randomized controlled trials. PLoS Med 2010;7(3):e1000252.

41. Ramsden CE, Zamora D, Leelarthaepin B et al. Use of dietary linoleic acid for secondary prevention of coronary heart disease and death: evaluation of recovered data from the Sydney Diet Heart Study and updated meta-analysis. BMJ 2013;346:e8707.

42. Smit LA, Mozaffarian D, Willett W. Review of fat and fatty acid requirements and criteria for developing dietary guidelines. Ann Nutr Metab 2009;55(1-3):44-55.

For peer review only - http://bmjopen.bmj.com/site/about/guidelines.xhtml

18 October 2017 V2

Table 1
Descriptive characteristics at baseline assessment of participants in the COB trial according to allocation (intention to treat)

	Coconut oil N=29		Butter N=33		Olive Oil N=32	
	**Mean**	**(SD)**	**Mean**	**(SD)**	**Mean**	**(SD)**
Age (years)	59.1	(6.1)	61.5	(5.8)	59.1	(6.4)
LDL-Cholesterol (mmol/l)	3.5	(0.9)	3.5	(0.9)	3.7	(1.0)
Total cholesterol (mmol/l)	5.9	(1.0)	5.9	(1.0)	6.0	(0.9)
HDL-Cholesterol (mmol/l)	2.0	(0.5)	1.9	(0.5)	1.8	(0.5)
Cholesterol/HDL ratio	3.2	(0.9)	3.2	(0.8)	3.5	(1.2)
Non HDL-Cholesterol (mmol/l)	3.9	(1.0)	4.0	(0.9)	4.2	(1.1)
Glucose (mmol/l)	5.3	(0.4)	5.4	(0.5)	5.4	(0.5)
Weight (kg)	73.9	(15.1)	70.8	(11.7)	71.1	(14.5)
Waist (cm)	85.4	(11.9)	83.7	(8.1)	86.2	(11.5)
Body fat (%)	29.7	(10.2)	29.2	(9.0)	31.5	(9.6)
Body Mass Index (kg/m2)	25.5	(4.5)	24.8	(3.5)	25.0	(4.5)
Systolic blood pressure (mmHg)	131.4	(18.8)	136.5	(18.8)	133.1	(16.5)
Diastolic blood pressure (mmHg)	79.8	(9.3)	81.0	(12.0)	78.1	(6.7)
DietWebQ intake/day						
Total energy (MJ)	9.00	(3.70)	8.23	(2.17)	9.51	(3.5)
Protein % energy	14.8	(4.4)	16.0	(3.7)	15.7	(3.0)
Carbohydrate % energy	43.6	(8.9)	41.4	(8.7)	42.7	(11.7)
Total fat% energy	37.3	(7.3)	36.7	(8.7)	36.4	(10.3)
Saturated fat% energy	14.1	(3.6)	13.3	(4.4)	13.4	(4.9)
Alcohol % energy	4.2	(5.4)	5.9	(7.5)	5.1	(6.1)
Hours of walking in past week	8.9	(9.5)	10.9	(12.3)	10.1	(8.7)
Hours of cycling in past week	1.8	(2.6)	2.0	(2.5)	2.7	(5.5)
Hours of other physical exercise in past week	3.4	(3.4)	2.3	(4.0)	1.8	(2.6)

For peer review only

For peer review only - http://bmjopen.bmj.com/site/about/guidelines.xhtml

18 October 2017 V2

Table 1 continued Descriptive characteristics at baseline assessment of participants in the COB trial according to allocation (intention to treat)

	Coconut oil N=29		Butter N=33		Olive Oil N=32	
	**Median (IQR)**		**Median (IQR)**		**Median (IQR)**	
Triglycerides (mmol/l)	0.89	(0.74,1.10)	0.92	(0.70,1.20)	0.94	(0.79,1.31)
C-Reactive Protein (mg/l)	1.04	(0.47,2.15)	1.08	(0.64,2.13)	1.13	(0.58,2.67)
	**%**	**(N)**	**%**	**(N)**	**%**	**(N)**
Sex						
Men	37.9	(11)	33.3	(11)	28.1	(9)
Women	62.1	(18)	66.7	(22)	71.9	(23)
Ethnicity						
White	96.6	(28)	97.0	(32)	93.8	(30)
Non-white	3.4	(1)	3.0	(1)	3.1	(1)
Smoking status						
Never	58.6	(17)	66.7	(22)	68.8	(22)
Former	34.5	(10)	33.3	(11)	25.0	(8)
Current	6.9	(2)	0.0	(0)	6.3	(2)
Alcohol consumption in past year						
Never or once per month	20.7	(6)	30.3	(10)	28.1	(9)
1-4 times per week	72.4	(21)	48.5	(16)	59.4	(19)
Almost every day or every day	6.9	(2)	21.2	(7)	12.5	(4)
Highest level of education						
School to age 16	13.8	(4)	12.1	(4)	15.6	(5)
School to age 18	27.6	(8)	9.1	(3)	9.4	(3)
University	58.6	(17)	78.8	(26)	75.0	(24)
Currently in paid job						
No	20.7	(6)	45.5	(15)	25.0	(8)
Yes	75.9	(22)	54.5	(18)	75.0	(24)

IQR: Interquartile range

For peer review only - http://bmjopen.bmj.com/site/about/guidelines.xhtml

18 October 2017 V2

Table 2
Mean change in variables between baseline and follow up after dietary interventions and pairwise comparisons between fats in 91 participants

	Change from Baseline				Pairwise comparisons		
	Coconut oil N=28 Mean (SD)	Butter N=33 Mean (SD)	Olive Oil N=30 Mean (SD)	P value Comparison Between groups	Coconut oil vs olive oil Difference (95% CI)	Butter vs Coconut oil Difference (95% CI)	Butter vs olive oil Difference (95% CI)
LDL-Cholesterol mmol/L	-0.09 (0.49)	0.33 (0.48)	-0.06 (0.39)	<0.001	-0.04 (-0.27, 0.19)	0.42 (0.19,0.65)	0.38 (0.16,0.60)
Total cholesterol mmol/L	0.22 (0.55)	0.42 (0.59)	0.03 (0.43)	0.022	0.19 (-0.08,0.46)	0.19(-0.08,0.45)	0.38 (0.11,0.64)
HDL-Cholesterol mmol/L	0.28 (0.29)	0.09 (0.27)	0.10 (0.15)	0.009	0.16 (0.03,0.28)	-0.18 (-0.30,-0.06)	-0.02 (-0.14,0.09)
Triglycerides mmol/L	0.07 (0.58)	0.00 (0.36)	-0.03 (0.27)	0.71	0.09 (-0.13,0.30)	-0.07 (-0.28,0.14)	0.02 (-0.19,0.23)
Cholesterol/HDL ratio	-0.26(0.36)	0.10 (0.41)	-0.13 (0.32)	<0.001	-0.14 (-0.33,0.05)	0.36 (0.18,0.54)	0.22 (0.04,0.40)
Non HDL-Cholesterol mmol/L	-0.06 (0.44)	0.33 (0.51)	-0.07 (0.42)	<0.001	0.00 (-0.23,0.24)	0.39 (0.16,0.62)	0.39 (0.16,0.62)
Glucose mmol/L	-0.05 (0.49)	0.02 (0.48)	-0.06 (0.49)	0.68	0.01 (-0.23,0.25)	0.08(-0.15,0.32)	0.09 (-0.14,0.33)
C-Reactive Protein mg/L	-0.31 (1.09)	-0.04 (0.93)	0.23 (1.40)	0.11	-0.59 (-1.14,-0.05)	0.29 (-0.24,0.82)	-0.30 (-0.82,0.22)
Weight Kg	0.27 (0.77)	0.04 (1.00)	-0.04 (0.84)	0.32	0.27 (-0.39,0.93)	0.50 (-0.14,1.14)	-0.23 (-0.86,0.40)
Waist cm	1.29 (3.31)	0.26 (3.43)	0.59 (3.25)	0.52	0.71 (-1.00,2.42)	0.95 (-0.72,2.63)	-0.24 (-1.89, 1.41)
Body fat %	0.24 (1.03)	0.34 (1.31)	0.51 (2.51)	0.82	0.09 (-0.54,0.73)	-0.10 (-0.72,0.52)	0.19 (-0.42, 0.81)
Body Mass Index kg/m2	0.09 (0.27)	0.02 (0.35)	-0.01 (0.29)	0.13	0.10 (-0.06,0.26)	0.07 (-0.09,0.22)	0.03 (-0.12, 0.18)
Systolic blood pressure mm Hg	0.2 (11.5)	-3.8 (11.1)	-3.7 (10.4)	0.26	3.9 (-1.3, 9.2)	3.7 (-1.4, 8.9)	0.2 (-4.9,5.3)
Diastolic blood pressure mm Hg	-2.0 (5.7)	-1.3 (6.2)	-0.5 (8.5)	0.81	-0.7 (-3.9, 2.4)	-1.0 (-4.1,2.1)	0.3 (-2.8,3.3)

For peer review only - http://bmjopen.bmj.com/site/about/guidelines.xhtml

For peer review only

Table 3
Baseline and follow up dietary intake by allocation to coconut oil, butter or olive oil* estimated using 24 hour DietWebQ

DietWebQ intake/day	Coconut oil	Butter	Olive oil
Baseline prior to start of intervention	N=27	n=33	n=32
Energy MJ/d	9.0 (3.7)	8.2 (2.2)	9.5 (3.5)
Total fat g/d	94 (47)	81 (26)	98 (50)
Protein g/d	74 (29)	75 (19)	87 (34)
Carbohydrate g/d	238 (95)	215 (75)	243(95)
Alcohol g/d	16(22)	17 (23)	18(22)
At four weeks of intervention	n=24	n=32	n=27
Energy MJ/d	9.6 (3.2)	8.6 (2.4)	9.6 (3.1_
Total fat g/d	127 (47)	94 (37)	138 (38)
Protein g/d	71 (25)	77 (29)	78 (31)
Carbohydrate g/d	215 (84)	214 (64)	197 (101)
Alcohol g/d	9 (15)	13(15)	8(18)
Change from baseline	n=24	n=32	n=27
Energy MJ/d	0.3 (2.9)	0.5 (2.0)	-0.4 (2.8)
Total fat g/d	29 (43)	14 (36)	28 (40)
Protein g/d	-7 (33)	3 (30)	-12 (26)
Carbohydrate g/d	-31 (74)	4 (69)	-55(81)
Alcohol g/d	-8 (22)	-5(23)	-11 (27)

*numbers do not total 94 as not all participants completed the baseline and follow up DietWebQ

For peer review only

For peer review only - http://bmjopen.bmj.com/site/about/guidelines.xhtml

18 October 2017 V2

Table 4 supplemental

Mean change in variables between baseline and follow up after dietary interventions in 71 participants who reported no change in physical activity during the trial

	Change from Baseline			
	Coconut oil N=22 Mean (SD)	Butter N=24 Mean (SD)	Olive Oil N=25 Mean (SD)	P value Comparison Between groups
LDL-Cholesterol mmol/L	-0.10 (0.50)	0.20 (0.53)	-0.04 (0.35)	0.01
Total cholesterol mmol/L	0.19 (0.59)	0.38 (0.63)	0.07 (0.37)	0.13
HDL-Cholesterol mmol/L	0.31 (0.29)	0.10 (0.26)	0.12 (0.16)	0.001
Triglycerides mmol/L	-0.02 (0.46)	-0.01 (0.42)	-0.04 (0.23)	0.97
Cholesterol/HDL ratio	-0.30(0.35)	0.07 (0.44)	-0.13 (0.30)	0.004
Non HDL-Cholesterol mmol/L	-0.11 (0.44)	0.28 (0.56)	-0.06 (0.36)	0.008
Glucose mmol/L	-0.12 (0.49)	-0.02 (0.52)	-0.08 (0.51)	0.80
C-Reactive Protein mg/L	-0.30 (1.18)	-0.13 (0.86)	0.04 (1.00)	0.51
Weight Kg	0.13 (0.62)	0.07 (1.06)	-0.02 (0.76)	0.83
Waist cm	1.47 (3.35)	0.67 (3.48)	0.81 (3.48)	0.70
Body fat %	0.34 (1.11)	0.23 (1.37)	0.81 (1.37)	0.71
Body Mass Index kg/m2	0.04 (0.22)	0.03 (0.37)	0.00 (0.26)	0.85
Systolic blood pressure mm Hg	-3.1 (8.9)	-5.1 (11.3)	-2.4 (7.8)	0.60
Diastolic blood pressure mm Hg	-2.4 (5.6)	-2.0 (6.6)	0.8 (8.4)	0.24

For peer review only - http://bmjopen.bmj.com/site/about/guidelines.xhtml

18 October 2017 V2

Figure 1

Recruitment and flow diagram for Coconut Oil, Olive Oil or Butter Trial (CONSORT)

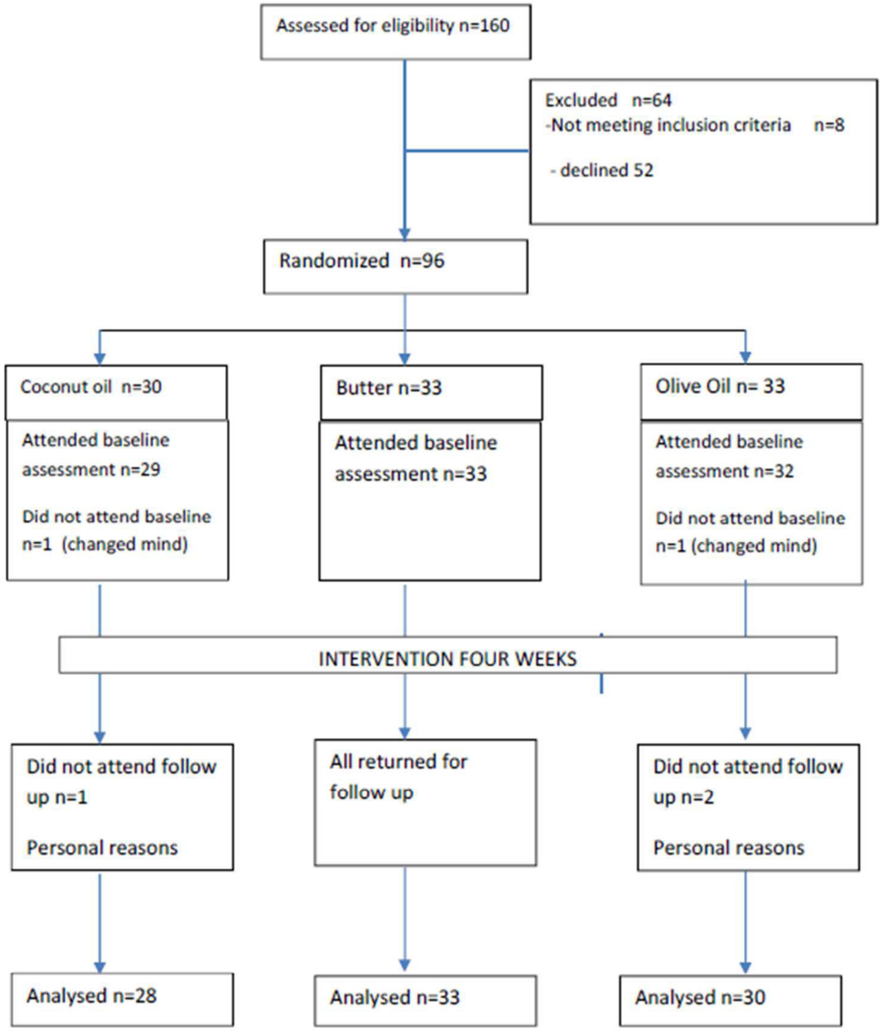

18 October 2017 V2

Figure 2

Difference (95% CI) in the primary outcome (LDL cholesterol) between each pair of randomised groups, reported in units of baseline SD. Mean (SD) change from baseline is also presented for each group in mmol/l. COB study, Intention to Treat population n=91

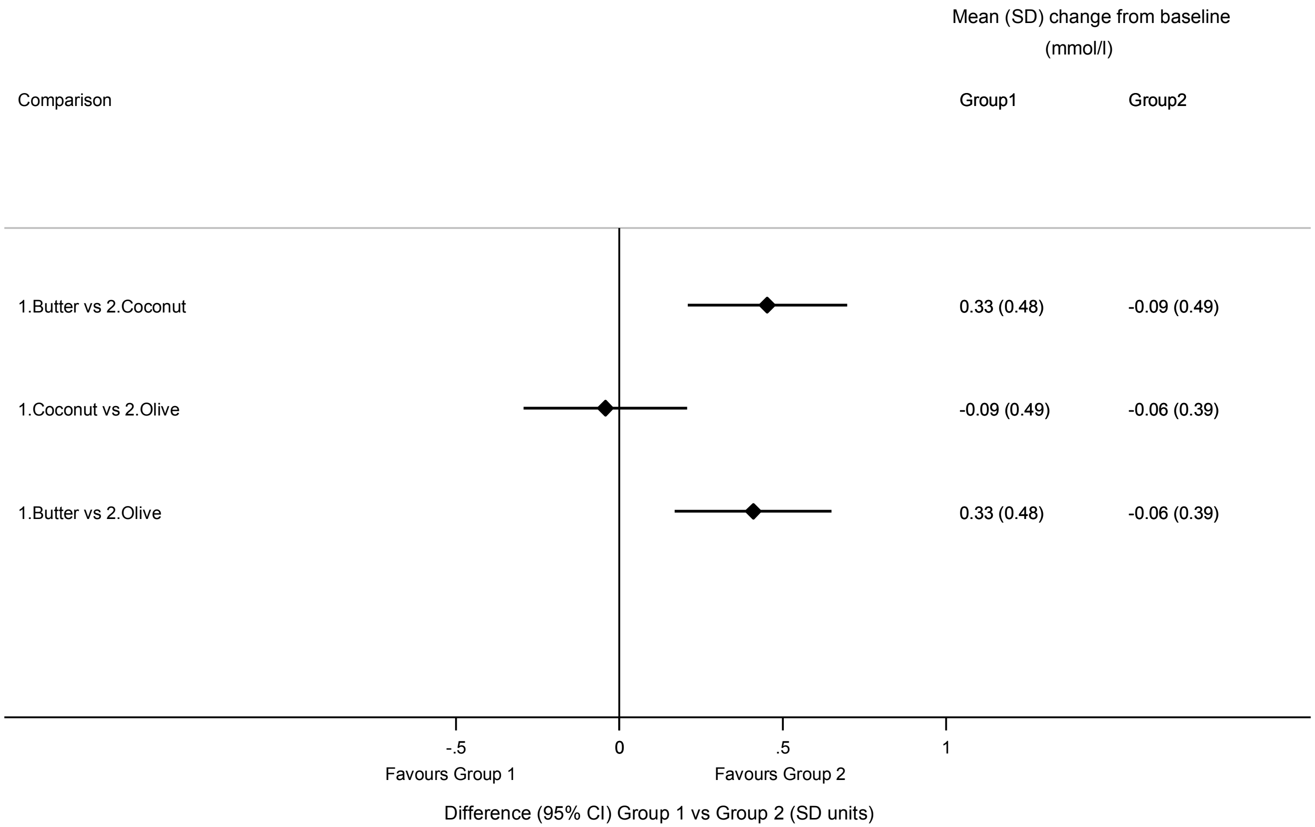

For peer review only - http://bmjopen.bmj.com/site/about/guidelines.xhtml

18 October 2017 V2

Figure 3

Difference (95% CI) in secondary outcomes comparing Butter vs Coconut Oil groups, reported in units of baseline SD. Mean (SD) change from baseline is also presented for each group in the natural units of the outcome. COB study, Intention to Treat population n=91

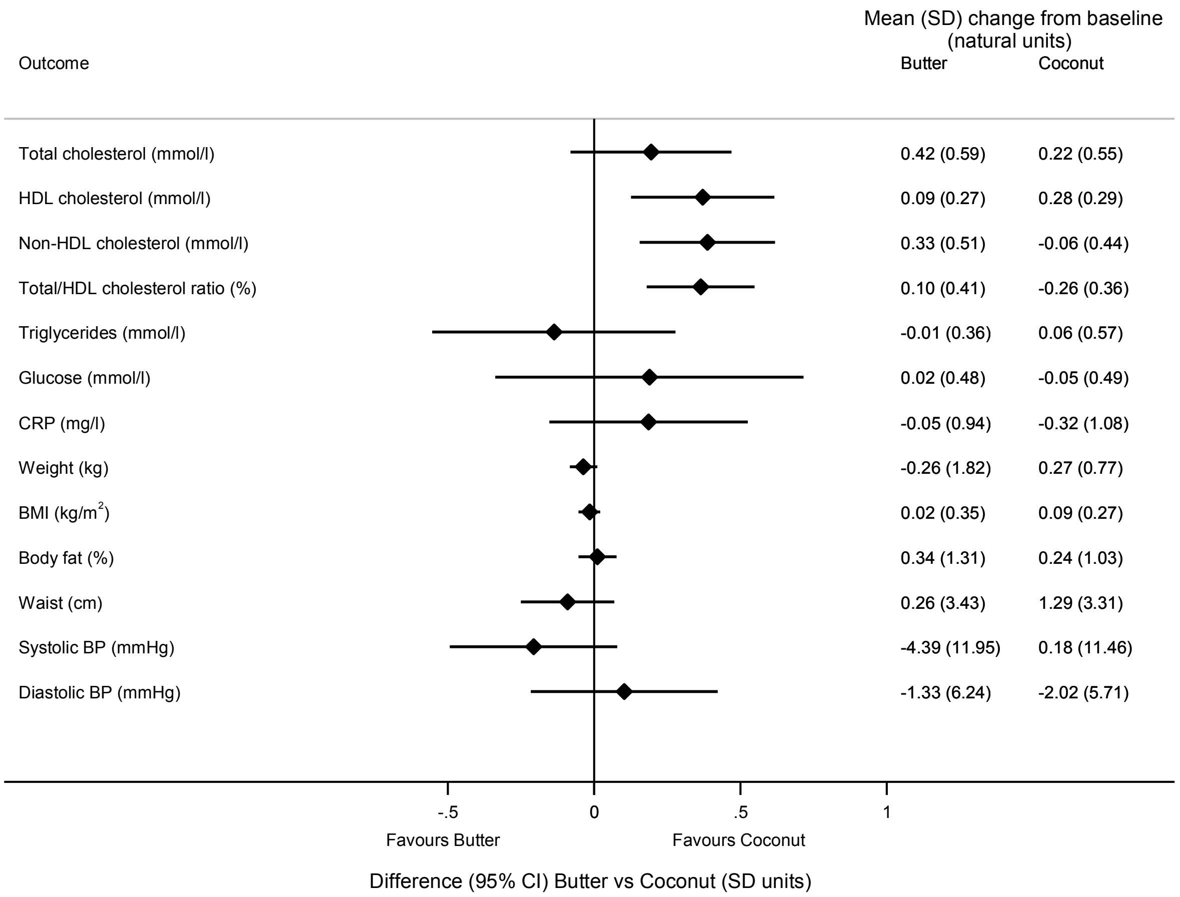

For peer review only - http://bmjopen.bmj.com/site/about/guidelines.xhtml

18 October 2017 V2

Figure 4

Difference (95% CI) in secondary outcomes comparing Coconut Oil vs Olive Oil groups, reported in units of baseline SD. Mean (SD) change from baseline is also presented for each group in the natural units of the outcome. COB study, Intention to Treat population n=91.

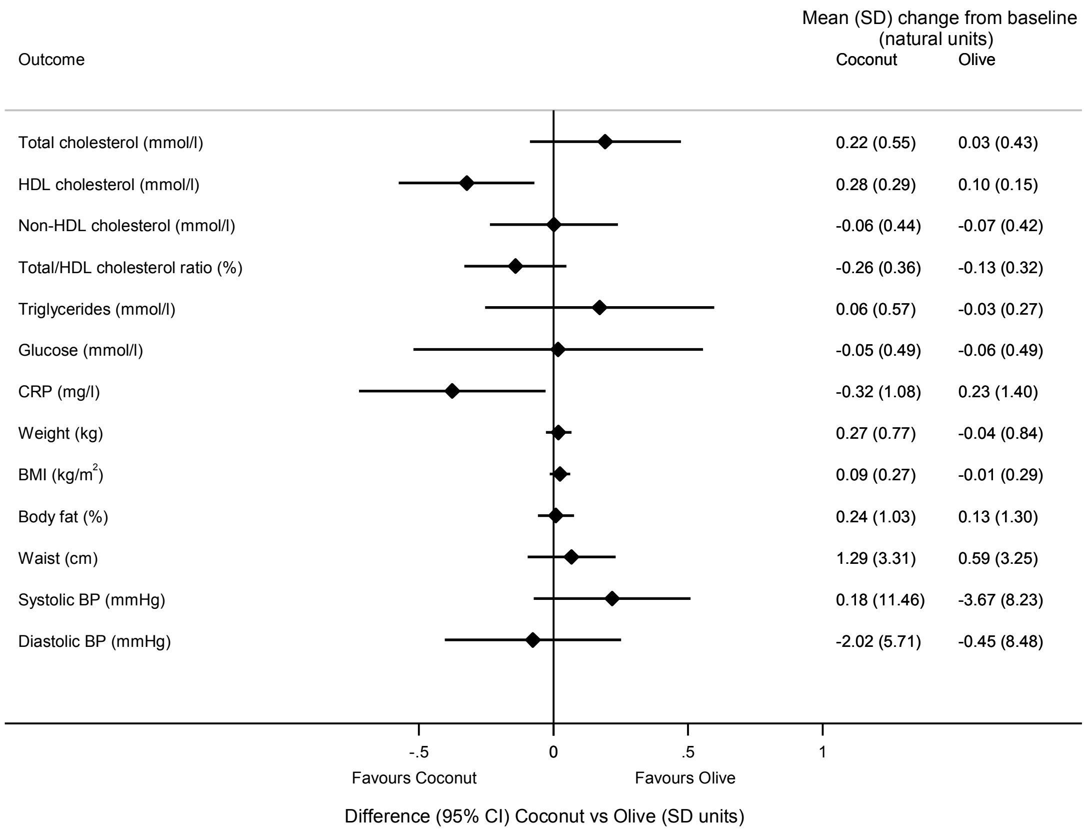

For peer review only - http://bmjopen.bmj.com/site/about/guidelines.xhtml

Figure 5

Difference (95% CI) in secondary outcomes comparing Butter vs Olive Oil groups, reported in units of baseline SD. Mean (SD) change from baseline is also presented for each group in the natural units of the outcome. COB study, Intention to Treat population n=91.

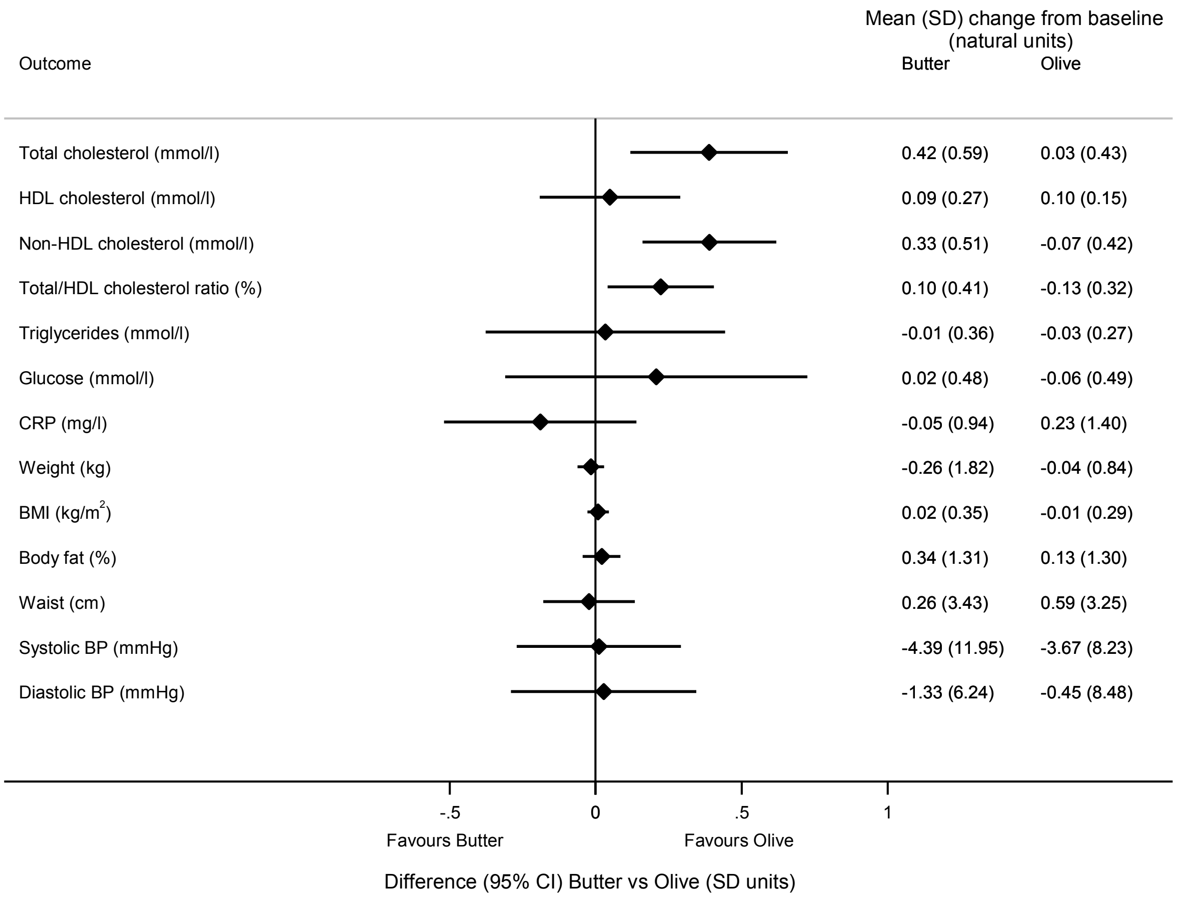

For peer review only - http://bmjopen.bmj.com/site/about/guidelines.xhtml

18 October 2017 V2

Appendix 1: Fatty acid composition of fats

Samples of the fats/oils used in the trial were sent for fatty acid composition to West Yorkshire Analytical Services, a UKAS accredited testing service for food composition. The results are tabulated below.

Coconut oil was 94 % saturated fatty acids, of which the main components were lauric acid C12:0 (48%) and myristic acid C14:0 (19%), palmitic acid C16:0 (9%) and caprylic acid C8:0 (9%); and 5% mono unsaturated fat, mainly oleic acid C18:1n9 (5%).

Butter was 66% saturated fatty acids, of which the main components were palmitic acid C16:0 (28%), stearic acid C18:0 (12%), myristic acid C14:0 (11%); 26% monounsaturated fat, mainly oleic acid C18:1n9 (22%); and 3% polyunsaturated fat, linoleic acid C18:2n6 (2%) and alpha-linolenic acid (1%).

Olive oil was 19% saturated fatty acids, mainly palmitic acid C16:0, 15% with stearic acid C18:0 (3%); 68% monounsaturates with the main component being oleic acid C18:1n9 (64%); and 13% polyunsaturates Linoleic acid C18:2n6 (12%).

		Coconut oil	Olive Oil	Butter
		% composition	% composition	% composition
C4:0	Butyric acid	<1	<0.1	2.5
C6:0	Caproic acid	0.7	<0.1	1.9
C8:0	Caprylic acid	8.6	<0.1	1.2
C10:0	Capric acid	6.3	<0.1	2.5
C12:0	Lauric acid	47.6	<0.1	3
C14:0	Myristic acid	18.6	<0.1	10.6
C14:1		<0.1	<0.1	0.9
C15:0		<0.1	<0.1	1.1
C16:0	Palmitic acid	8.6	14.8	28.1
C16:1	Palmitoleic acid	<0.1	1.5	1.4
C17:0		<0.1	<0.1	0.6
C17:1		<0.1	<0.1	0.4
C18.0	Stearic Acid	3.4	3	12.4
C18:1t			<0.1	3.2
C18:1n9	Oleic Acid	5.2	63.5	22.2
C181n7	cis-Vaccenic Acid	<0.1	2.8	0.4

For peer review only - http://bmjopen.bmj.com/site/about/guidelines.xhtml

18 October 2017 V2

C18:2tt		<0.1	<0.1	0.5
C18:2ct		<0.1	<0.1	0.1
C18:2tc		<0.1	<0.1	0.2
C18:2n6	Linoleic Acid	0.8	11.9	1.9
C18:3n6	Gamma Linolenic Acid	<0.1	<0.1	<0.1
C18:3n3	Alpha-Linolenic Acid	<0.1	<0.1	0.9
C20:0	Arachidic acid	<0.1	<0.1	0.2
C20:2n6	Eicosadienoic acid	<0.1	<0.1	<0.1
C18:4n3	Stearidonic acid	<0.1	0.2	0.1
C20:1	Paullinic acid	<0.1	<0.1	<0.1
C22:0	Behenic Acid	<0.1	0.2	0.1
C22:1n9	Erucic Acid	<0.1	<0.1	0.1
C22:2	Docosadienoic acid	<0.1	0.6	<0.1
C24:0	Lignoceric acid	<0.1	<0.1	<0.1
	Saturates	93.9	18.6	66.2
	Monounsaturates	5.2	68	26.1
	Polyunsaturates	0.7	13.5	3.4
	Transesters	<0.1	<0.1	4.2

For peer review only - http://bmjopen.bmj.com/site/about/guidelines.xhtml

CONSORT 2010 checklist of information to include when reporting a randomised trial*

Section/Topic	Item No	Checklist item	Reported on page No
**Title and abstract**			
	1a	Identification as a randomised trial in the title	1
	1b	Structured summary of trial design, methods, results, and conclusions (for specific guidance see CONSORT for abstracts)	2,3
**Introduction**			
Background and objectives	2a	Scientific background and explanation of rationale	5,6
	2b	Specific objectives or hypotheses	6
**Methods**			
Trial design	3a	Description of trial design (such as parallel, factorial) including allocation ratio	7,8
	3b	Important changes to methods after trial commencement (such as eligibility criteria), with reasons	N/A
Participants	4a	Eligibility criteria for participants	7
	4b	Settings and locations where the data were collected	7,8
Interventions	5	The interventions for each group with sufficient details to allow replication, including how and when they were actually administered	7,8
Outcomes	6a	Completely defined pre-specified primary and secondary outcome measures, including how and when they were assessed	8,9
	6b	Any changes to trial outcomes after the trial commenced, with reasons	N/A
Sample size	7a	How sample size was determined	9,10
	7b	When applicable, explanation of any interim analyses and stopping guidelines	N/A
Randomisation:			
Sequence generation	8a	Method used to generate the random allocation sequence	7
	8b	Type of randomisation; details of any restriction (such as blocking and block size)	7
Allocation concealment mechanism	9	Mechanism used to implement the random allocation sequence (such as sequentially numbered containers), describing any steps taken to conceal the sequence until interventions were assigned	7
Implementation	10	Who generated the random allocation sequence, who enrolled participants, and who assigned participants to interventions	7
Blinding	11a	If done, who was blinded after assignment to interventions (for example, participants, care providers, those	8,9

For peer review only

For peer review only - http://bmjopen.bmj.com/site/about/guidelines.xhtml

		assessing outcomes) and how	
	11b	If relevant, description of the similarity of interventions	N/A
Statistical methods	12a	Statistical methods used to compare groups for primary and secondary outcomes	9,10
	12b	Methods for additional analyses, such as subgroup analyses and adjusted analyses	9
**Results**			
Participant flow (a diagram is strongly recommended)	13a	For each group, the numbers of participants who were randomly assigned, received intended treatment, and were analysed for the primary outcome	Figure 1
	13b	For each group, losses and exclusions after randomisation, together with reasons	Figure 1
Recruitment	14a	Dates defining the periods of recruitment and follow-up	2,7,8
	14b	Why the trial ended or was stopped	8
Baseline data	15	A table showing baseline demographic and clinical characteristics for each group	Figure 1, table 1
Numbers analysed	16	For each group, number of participants (denominator) included in each analysis and whether the analysis was by original assigned groups	Table 2, Figures 2-5
Outcomes and estimation	17a	For each primary and secondary outcome, results for each group, and the estimated effect size and its precision (such as 95% confidence interval)	Table 2, figures 2-6
	17b	For binary outcomes, presentation of both absolute and relative effect sizes is recommended	N/A
Ancillary analyses	18	Results of any other analyses performed, including subgroup analyses and adjusted analyses, distinguishing pre-specified from exploratory	12
Harms	19	All important harms or unintended effects in each group (for specific guidance see CONSORT for harms)	12
**Discussion**			
Limitations	20	Trial limitations, addressing sources of potential bias, imprecision, and, if relevant, multiplicity of analyses	17,18
Generalisability	21	Generalisability (external validity, applicability) of the trial findings	18
Interpretation	22	Interpretation consistent with results, balancing benefits and harms, and considering other relevant evidence	19
**Other information**			
Registration	23	Registration number and name of trial registry	1,3
Protocol	24	Where the full trial protocol can be accessed, if available	3
Funding	25	Sources of funding and other support (such as supply of drugs), role of funders	21

*We strongly recommend reading this statement in conjunction with the CONSORT 2010 Explanation and Elaboration for important clarifications on all the items. If relevant, we also recommend reading CONSORT extensions for cluster randomised trials, non-inferiority and equivalence trials, non-pharmacological treatments, herbal interventions, and pragmatic trials. Additional extensions are forthcoming: for those and for up to date references relevant to this checklist, see www.consort-statement.org.

For peer review only - http://bmjopen.bmj.com/site/about/guidelines.xhtml

BMJ Open

Randomized trial of coconut oil, olive oil or butter on blood lipids and other cardiovascular risk factors in healthy men and women

Journal:	*BMJ Open*
Manuscript ID	bmjopen-2017-020167.R1
Article Type:	Research
Date Submitted by the Author:	15-Dec-2017
Complete List of Authors:	Khaw, Kay-Tee; University of Cambridge, Department of Public Health and Primary Care<br>Sharp, Stephen; University of Cambridge, MRC Epidemiology Uniot<br>Finikarides, Leila; British Broadcasting Corporation<br>Afzal, Islam; Aston University, Aston Medical Research Institute<br>Lentjes, Marleen; University of Cambridge, Department of Public Health and Primary Care<br>Luben, Robert; University of Cambridge, Department of Public Health and Primary Care<br>Forouhi, Nita; University of Cambridge, MRC Epidemiology Unit
**Primary Subject Heading**:	Nutrition and metabolism
Secondary Subject Heading:	Cardiovascular medicine, Epidemiology
Keywords:	blood lipids, dietary fats, randomized trial, coconut oil, olive oil

SCHOLARONE™
Manuscripts

14 December 2017 V3

Randomized trial of coconut oil, olive oil or butter on blood lipids and other cardiovascular risk factors in healthy men and women

Kay-Tee Khaw[1], Stephen J. Sharp[2], Leila Finikarides[3], Islam Afzal[4], Marleen Lentjes[1], Robert Luben[1], and Nita G. Forouhi[2]

Authors' Affiliations:

Kay-Tee Khaw, MBBChir, FRCP, FMedSci, Professor

Marleen Lentjes, PhD, Research Nutritionist

Robert Luben, BSc, Bioinformatics

[1]Department of Public Health and Primary Care, University of Cambridge School of Clinical Medicine, United Kingdom;

Stephen J. Sharp MA, MSc, Senior Statistician

Nita G. Forouhi, MBBS, PhD, MRCP, FFPH, Professor

[2]Medical Research Council Epidemiology Unit, University of Cambridge School of Clinical Medicine, United Kingdom;

Leila Finikarides, MSc, Producer,

[3]BBC Television"Trust Me I'm a Doctor", BBC Glasgow, Glasgow, United Kingdom; now Winton Centre for Evidence Communication, University of Cambridge, United Kingdom

Islam Afzal, PhD, Research Facilitator

[4] Aston Medical Research Institute, Aston Medical School, Aston University, Birmingham, United Kingdom

Clinical trials registration: NCT03105947 Clinical Trials.gov USNIH

Short running title: **Coconut oil, butter or olive oil and blood lipids**

Word count: text (excluding abstract, tables & references) = 5438 abstract = 586

Number of tables and figures: 3 Tables, 5 Figures, 1 supplemental table

Key words: coconut oil, butter, olive oil, dietary fat, lipids, LDL-Cholesterol, randomized trial

Correspondence to:

Kay-Tee Khaw, Professor

Clinical Gerontology Unit Box 251, University of Cambridge School of Clinical Medicine, Addenbrooke's Hospital, Cambridge, CB3 0EQ, United Kingdom

e-mail: kk101@medschl.cam.ac.uk Phone: +44 1223 336927

For peer review only - http://bmjopen.bmj.com/site/about/guidelines.xhtml

Abstract

Introduction: High dietary saturated fat intake is associated with higher blood concentrations of LDL-cholesterol, an established risk factor for coronary heart disease. However, there is increasing interest in whether various dietary oils or fats with different fatty acid profiles such as extra virgin coconut oil may have different metabolic effects but trials have reported inconsistent results. We aimed to compare changes in blood lipid profile, weight, fat distribution, and metabolic markers after four weeks consumption of 50g daily of one of three different dietary fats: extra virgin coconut oil, butter, or extra virgin olive oil: in healthy men and women in the general population.

Design: Randomized clinical trial conducted over June and July 2017.

Setting: General community in Cambridgeshire, United Kingdom

Participants: Volunteer adults were recruited by the British Broadcasting Corporation (BBC) through their websites. Eligibility criteria were men and women aged 50-75 years, with no known history of cancer, cardiovascular disease or diabetes, not on lipid lowering medication, no contraindications to a high fat diet and willingness to be randomized to consume one of the three dietary fats for four weeks. Of 160 individuals initially expressing an interest and assessed for eligibility, 96 were randomized to one of three interventions; 2 individuals subsequently withdrew and 94 men and women attended a baseline assessment. Their mean age was 60 years, 67% were women, and 98% were European Caucasian. Of these, 91 men and women attended a follow up assessment four weeks later.

Intervention: Participants were randomized to extra virgin coconut oil, extra virgin olive oil, or unsalted butter and asked to consume 50g daily of one of these fats for four weeks, which they could incorporate into their usual diet or consume as a supplement.

Main Outcomes and Measures: The primary outcome was change in serum Low Density Lipoprotein cholesterol(LDL-C); secondary outcomes were change in total and high density lipoprotein cholesterol(TC and HDL-C), TC/HDL-C ratio, and non-HDL-C; change in weight, body mass index(BMI), waist circumference, percent body fat, systolic and diastolic blood pressure, fasting plasma glucose and C-Reactive Protein.

Results: LDL-C concentrations were significantly increased on butter compared to coconut oil (+0.42, 95% CI 0.19,0.65 mmol/L, P<0.0001), and to olive oil (+0.38, 95% CI 0.16,0.60 mmol/L, P<0.0001), with no differences in change of LDL-C in coconut oil compared to olive oil (-0.04, 95% CI -0.27, 0.19 mmol/L, P=0.74). Coconut oil significantly increased HDL-C compared to

For peer review only

For peer review only - http://bmjopen.bmj.com/site/about/guidelines.xhtml

butter (+0.18, 95% CI 0.06,0.30 mmol/L) or olive oil (+0.16, 95% CI 0.03,0.28 mmol/L). Butter significantly increased TC/HDL-C ratio and non-HDL-C compared to coconut oil but coconut oil did not significantly differ from olive oil for TC/HDL-C and non-HDL-C. There were no significant differences in changes in weight, BMI, central adiposity, fasting blood glucose, systolic or diastolic blood pressure amongst any of the three intervention groups.

Conclusions and Relevance: Two different dietary fats (butter and coconut oil) which are predominantly saturated fats, appear to have different effects on blood lipids compared to olive oil, a predominantly monounsaturated fat with coconut oil more comparable to olive oil with respect to LDL-C. The effects of different dietary fats on lipid profiles, metabolic markers and health outcomes may vary not just according to the general classification of their main component fatty acids as saturated or unsaturated but possibly according to different profiles in individual fatty acids, processing methods, as well as the foods in which they are consumed or dietary patterns. These findings do not alter current dietary recommendations to reduce saturated fat intake in general but highlight the need for further elucidation of the more nuanced relationships between different dietary fats and health.

Clinical trials registration: NCT03105947 Clinical Trials.gov USNIH

For peer review only

For peer review only - http://bmjopen.bmj.com/site/about/guidelines.xhtml

Strength and limitations of the study

Strengths

- The randomized trial design comparing three dietary fat interventions minimised confounding and bias
- There was good compliance and participants were from the general community in a "real life" setting
- Objective measures of outcome- blood biochemistry and anthropometry – were used minimising bias

Limitations

- Participants were not blinded as to the intervention and the intervention was relatively short term over four weeks

For peer review only

For peer review only - http://bmjopen.bmj.com/site/about/guidelines.xhtml

Introduction

This trial was conducted in the context of debate over longstanding dietary recommendations to reduce dietary fat intake for health. The Women's Health Initiative reported no differences in cardiovascular disease in women randomized to low fat and usual diets over 8 years[1] while an intervention comparing a low fat diet with a Mediterranean diet with extra virgin olive oil, or nuts (PREDIMED) reported approximately 30% lower cardiovascular events in both Mediterranean diet arms after 4.8 years[2]; meta-analyses of observational studies and trials report inconsistent findings in the relationship between dietary saturated fatty acids and cardiovascular disease[3, 4]; and the relationships of dairy fats including milk and butter with cardiovascular disease also being debated[5-7]. Part of the debate relates to the increasing evidence that different individual fatty acids, such as the odd chain or even chain saturated fatty acids, or short, medium and long chain saturated fatty acids, may have different metabolic pathways and subsequent potential health effects, as well as the understanding that diet is more complex than individual nutrients or generic biochemical nutrient groups, and that contextual factors such as foods and dietary patterns are important. The 2015-2020 US dietary guidelines[8] now focus on foods and dietary patterns and while they recommend limiting saturated and trans fats, they no longer explicitly recommend limiting total fat. In this context therefore, there is renewed interest in the health effects of different fats and oils.

Extra virgin coconut oil has recently been promoted as a healthy oil. Though high in saturated fat, the main saturated fatty acid, lauric acid(c12:0), has been suggested to have different metabolic, and hence health effects compared to other saturated fatty acids such as palmitic acid(c16:0), predominant in butter, palm oil and animal fat. In particular, it has been suggested that coconut oil does not raise total cholesterol or LDL-Cholesterol as much as butter. A recent review on coconut oil and cardiovascular risk factors in humans concluded that the evidence of an association between coconut oil consumption and blood lipids or cardiovascular risk was mostly poor quality[9]. While some small studies have been reported comparing coconut oil and butter, these have been small[10, 11], and none conducted in the UK where overall dietary patterns are different from Asia, US or New Zealand where most trials have been conducted. The 2017 American Heart Association Presidential advisory on dietary fats and cardiovascular disease highlighted the paucity of evidence over the long term health effects of saturated fats such as coconut oil and reinforced strongly recommendations to

For peer review only - http://bmjopen.bmj.com/site/about/guidelines.xhtml

lower dietary saturated fat and replacement with unsaturated fat to lower LDL-cholesterol and prevent cardiovascular disease[12]. In particular, they stated “because coconut oil increases LDL-Cholesterol, a cause of cardiovascular disease, and has no known offsetting favourable effects, we advise against the use of coconut oil”[12].

Though the PREDIMED study reported lower cardiovascular disease events in those randomized to extra virgin olive oil or added nuts[2], this trial reported no overall effects on LDL-cholesterol or total cholesterol for those on olive oil compared to the low fat diet [13], results consistent with a review of intervention trials of high phenolic olive oil[14].

We therefore aimed to examine whether in free living healthy men and women in the UK, we could observe differences in blood lipids after one month’s consumption of 50g daily of one of three different fats within the context of their usual diet. Although this was a short term trial that did not address cardiovascular disease events, blood lipids are a well established risk factor for coronary heart disease and the aim was to compare directly the effects of three different fats: extra virgin coconut oil, butter (both predominantly saturated fats) with extra virgin olive oil (monounsaturated fat) on blood lipid profiles and metabolic measures, in a pragmatic trial using amounts feasible in daily diets.

For peer review only

For peer review only - http://bmjopen.bmj.com/site/about/guidelines.xhtml

Methods

Study population

Participants were volunteers living in the general community predominantly in the Cambridgeshire area, recruited through BBC advertising in May and June 2017. Eligible participants were men or women aged between 50-75 years who did not have a known medical history of heart disease, stroke, cancer, or diabetes, and who were not taking medication for lowering blood lipids such as statins. They had to be willing to be randomized to consume 50 g daily of one of the designated fats for four weeks, and not have any contraindications to eating a high fat diet such as gall bladder or bowel problems. Of 160 individuals expressing an interest, 96 were eligible and randomized to the intervention, 2 withdrew prior to the start of the study, and 94 attended a baseline assessment.

Allocation to Intervention

Participants were assigned a unique study identification number(ID). These ID numbers were randomized by computer generated allocation conducted by an independent statistician separately in men and women, into one of three parallel intervention arms approximately equal in size: extra virgin coconut oil, butter, or extra virgin olive oil.

Intervention

Participants attending the baseline assessment, at the end of their appointment, received one month's supply of one of the three different dietary fats to which they had been randomly allocated: extra virgin coconut oil, or butter or extra virgin olive oil. The BBC study organizer was given an ID list with the random allocation to the fats/oils and was responsible for giving each participant their supply of fat/oils. They were asked to eat 50g of these fats daily for four weeks and given measuring cups for the 50ml fat and oils: butter was prepacked in 20g and 30g portions. They were asked to continue with their usual diet, and either incorporate the fat or oil into their daily diet to substitute for other fats or oils, or they could eat these fats as a supplement. They also had information sheets with suggestions for how the fats could be consumed including recipes. The fats selected were standard products available from supermarkets bought from suppliers; organic extra virgin coconut oil, organic unfiltered extra virgin olive oil, and organic unsalted butter. Samples of the oils/fats used in the trial were sent to a reference laboratory: the West Yorkshire Analytic Services, a UKAS accredited testing service for food composition.

For peer review only - http://bmjopen.bmj.com/site/about/guidelines.xhtml

Assessments

Participants attended two assessments at a community centre in Cambridge: one at baseline before the start of the intervention in June 2017, and one at the end of four weeks in July 2017. Prior to their initial assessment, they were asked to fill in a short questionnaire about their health and lifestyle including physical activity and diet as well as complete an online 24 hour dietary assessment questionnaire with automated nutrient intake estimation, developed in Oxford, the DietWebQ[15]. All assessments were conducted between 0800 and 1230. Participants were all fasted for a minimum of 4 hours prior to attending the assessment; the majority were fasted overnight. They had height and waist circumference measured to a standardised protocol in light clothing without shoes and blood pressure measured using an automated OMRON device after being seated resting for 5 minutes. The mean of two readings for blood pressure, height and waist were used for analysis. Weight and percent body fat were measured using a Tanita body composition monitor. All measurements were conducted by two trained observers unaware of allocation to the oils/fats. Participants gave a 20 ml blood sample which was stored in a 4°C refrigerator then sent to the laboratory by courier for same day sample processing and storage for later analysis.

After four weeks at the end of the intervention, they attended again for a follow up assessment where the same measurements of height, waist circumference, blood pressure, weight and percent body fat were conducted, and another fasting 20 ml blood sample taken. Measurements were recorded on new forms and observers and participants did not have access to the measurements taken at the baseline visit. Just prior to this visit, participants were asked to fill in again the online 24 hour DietWebQ. Participants also filled in short questionnaire about their experiences on the intervention fats. This included a question about their overall experience of consuming the assigned oil/fat in the study where they were asked on average, over the past 4 weeks whether they felt mostly the same as usual, mostly felt better than usual or mostly felt worse than usual with an open ended section for comments including side effects, and overall compliance with consuming the fats which they were asked to self-rate between 0% to 100%. They were also asked whether they changed their type, level or frequency of physical activity in the past month since being in the study and had three options, no overall change in activity, increase in activity or decrease in activity.

For peer review only - http://bmjopen.bmj.com/site/about/guidelines.xhtml

Blood samples were identified only by a study ID number and were processed using standard protocols and assayed in two batches at the end of the baseline and follow up assessments in the Core Biochemical Assay Laboratory (CBAL) Cambridge University Hospitals which has UKAS Clinical Pathology Accreditation; blood samples from individuals on different interventions were thus all assayed in the same batch. The laboratory assays were conducted in a blinded fashion without any indication of the allocated intervention. Cholesterol(TC) and triglycerides were measured using enzymatic assays,[16, 17] high-density-lipoprotein cholesterol (HDL-C) was measured using a homogenous accelerator selective detergent assay automated on the Siemens Dimension RxL analyser, and low density lipoprotein cholesterol(LDL-C) was calculated from the triglyceride, HDL and cholesterol concentrations as described in the Friedewald formula (LDL = Cholesterol - HDL - (Triglycerides/2.2)[18]. Total to HDL-C ratio was computed, and non-HDL-C was computed as TC minus HDL-C.

Plasma glucose was measured using the hexokinase-glucose-6-phosphate dehydrogenase method and high sensitivity human C-Reactive Protein was assayed using an automated colourimetric immunoassay: Siemens Dimension® CCRP *Cardio*Phase® high sensitivity CRP.

Trial outcomes

The trial was registered in April 2017 with clinical trials registration: NCT03105947**.** The primary outcome of the trial was change in low density lipoprotein cholesterol (LDL-C) from baseline to follow up. Secondary outcomes were change in each of the following variables from baseline to follow up: total cholesterol (TC), high density lipoprotein cholesterol (HDL-C), triglycerides; ratio of total cholesterol/HDL-C, non-HDL cholesterol, fasting blood glucose, C-Reactive Protein, weight, body mass index(BMI), body fat %, waist circumference, systolic blood pressure and diastolic blood pressure..

Statistical analysis

The study aimed to recruit a total of 90 participants: 30 individuals per group would provide approximately 80% power to detect a difference in mean within-person change in LDL cholesterol (baseline to follow-up) comparing pairs of randomized groups (butter vs coconut oil and butter vs olive oil) of approximately 0.5 mmol/L, assuming a standard deviation of LDL cholesterol of 1.04 mmol/L[19] and a correlation between baseline and follow-up values of 0.79[20] incorporated using the method described by Borm et al[21]. With 2 primary pairwise comparisons, the significance level for each comparison was set to 2.5%.

For peer review only - http://bmjopen.bmj.com/site/about/guidelines.xhtml

This magnitude of difference was what can be estimated from metabolic ward studies in which replacement of 10% dietary calories from saturated fat is associated with 0.52 mmol/L cholesterol difference[22] though this did not specify the food sources of saturated fats, and a small intervention trial (n=28) comparing butter and coconut oil with sunflower oil[10].

Baseline characteristics were summarised separately for each randomized group. As recommended by CONSORT, no p-values were calculated for this table. The primary analysis used an Intention To Treat(ITT) population, which included all individuals in the group to which they were randomized, regardless of the extent to which they adhered to the intervention. A secondary analysis used a Per Protocol(PP) population. This was a subset of the ITT population consisting of those individuals who adhered to the intervention. Participants who reported >75% adherence when asked at the follow up visit were included in the PP population.

For each outcome, a p-value was calculated to compare the 3 randomized groups using a linear regression model, in which change from baseline was the outcome, and including a dummy variable for randomized group and the baseline value of the outcome variable as covariates, i.e. an Analysis of Covariance (ANCOVA) model. Differences between each pair of randomized groups and 95% confidence intervals (CIs) were also estimated from a similar model.

Patient and public involvement

The BBC originally proposed the idea of a study to examine claims about the health benefits of coconut oil in response to public interest; the study would be part of their “Trust me, I’m a doctor” series The study was designed as a randomized trial with participants from the general community in discussion with the BBC.

Ethics

Ethics approval was given for the study by the University of Cambridge Human Biology Research Ethics committee HBREC 2017.05.

For peer review only - http://bmjopen.bmj.com/site/about/guidelines.xhtml

Results

Figure 1 is the CONSORT diagram for the trial. The recruitment was conducted by the BBC coordinator through BBC website advertising. From 160 individuals initially expressing an interest, and after exclusion criteria, 96 individuals were randomized and invited to a baseline assessment session in June 2017. Two individuals subsequently withdrew and 94 individuals attended the baseline assessment session in June 2017. At the four week follow up assessment in July 2017, 91 individuals attended; 3 individuals did not attend follow up indicating personal circumstances.

Table 1 shows descriptive characteristics for the participants at the baseline assessment according to the allocation to dietary oils/fats. Two thirds of the participants were women and the mean age overall was 60 years.

Table 2 shows mean changes in the primary and secondary outcomes at the four week follow up within each randomized group, and comparisons between each pair of randomized groups. LDL-C concentrations were significantly increased on butter compared to coconut oil (+0.42, 95% CI 0.19,0.65 mmol/L, P<0.0001), and olive oil (+0.38L, 95% CI 0.16,0.60 mmol/L, P<0.0001), with no differences in change of LDL-C in coconut oil compared with olive oil (-0.04, 95% CI -0.27, 0.19 mmol/L, P=0.74). Coconut oil significantly increased HDL-C compared to butter (+0.18, 95% CI 0.06,0.30 mmol/L) or olive oil (+0.16, 95% CI 0.03,0.28 mmol/L).

Butter significantly increased the cholesterol/HDL-C ratio compared to coconut oil (+0.36, 95%CI 0.18,0.54) and olive oil (+0.22,95% CI 0.04,0.40) and also increased non-HDL-C compared to coconut oil (+0.39, 95% CI 0.16,0.62 mmol/L) and olive oil (+0.39(95% CI 0.16,0.62) but coconut oil did not significantly differ from olive oil for change in cholesterol/HDL-C ratio (-0.14, 95%CI -0.33,0.05) or non-HDL-C (0.002, 95% CI -0.23,0.24 mmol/L).

Coconut oil also significantly lowered C-Reactive Protein in comparison with olive oil (-0.58, 95% CI -1.12,-0.04 mg/L) but not compared to butter. There were no significant differences in changes in weight, BMI, central adiposity, fasting blood glucose, systolic or diastolic blood pressure amongst any of the three intervention groups. For weight, for example, the

For peer review only - http://bmjopen.bmj.com/site/about/guidelines.xhtml

estimated mean(SD) changes in weight were +0.27(0.77)kg, 0.04(1.00)kg and -0.04(0.84) kg for coconut oil, butter and olive oil respectively.

Figure 2 shows the difference in the primary outcome (LDL-C) between each pair of randomized groups in the 91 individuals who attended baseline and follow up. **Figures 3, 4, and 5** show the differences in secondary outcomes comparing butter versus coconut oil, coconut oil versus olive oil, and butter versus olive oil respectively. For comparability the differences are reported in units of baseline standard deviation (SD) for the different outcomes in Figures 3 to 5.

Self reported compliance was high: 87% of participants reported more than 75% compliance with the intervention over the 4 weeks which was similar among the groups (86% coconut oil, 88% butter and 85% olive oil). Secondary analyses on the 82 participants reporting more than 75% compliance showed similar results (not shown). Reported experience consuming the fats was similar between groups: 57%, 66%, and 60% reported feeling no different, 18%, 6% and 13% reported feeling better, and 25%, 27% and 23% reported feeling worse in the coconut oil, butter and olive oil groups respectively. Comparison of dietary intake using the 24 hour DietWebQ showed similar levels of dietary intake across intervention groups at baseline. Following the intervention, total fat intake increased in all intervention groups but estimates for absolute intakes of carbohydrate, protein and alcohol did not differ between intervention groups (Table 3). Most of the participants reported no changes in usual physical activity (79%, 73% and 89% no change; 14%, 15% and 4% increased usual physical activity and 7%, 12% and 7% decreased usual physical activity in the coconut oil, butter and olive oil groups respectively). In a post hoc exploratory analysis, exclusion of individuals who reported increasing usual physical activity had little effect on significant differences between interventions for LDL-C and HDL-C and did not alter the findings for weight change (supplementary table 4).

Supplementary appendix 1 shows the fatty acid composition of the three oils/fats used in the intervention. Coconut oil was 94 % saturated fatty acids, of which the main components were lauric acid C12:0(48%), myristic acid C14:0(19%), and palmitic acid C16:0(9%). Butter was 66% saturated fatty acids, of which the main components were palmitic acid C16:0(28%), stearic

For peer review only - http://bmjopen.bmj.com/site/about/guidelines.xhtml

For peer review only

acid C18:0(12%), and myristic acid C14:0(11%). Olive oil was 19% saturated fatty acids, mainly palmitic acid C16 (15%) with stearic acid C18:0 (3%) and 68% monounsaturates with the main component being oleic acid C18:1n9(64%). These profiles are very similar to those reported from other studies[9].

For peer review only

For peer review only - http://bmjopen.bmj.com/site/about/guidelines.xhtml

Discussion

In this trial, middle aged men and women living in the general community were randomly allocated to consume 50g extra virgin coconut oil, or 50g butter, or 50g extra virgin olive oil for four weeks. We observed at the end of the trial significantly different changes in LDL-C and HDL-C concentrations between the three intervention groups; in pairwise comparisons, coconut oil did not significantly raise LDL-C concentrations compared to olive oil while butter significantly raised LDL-C concentrations compared to both coconut oil and olive oil. Coconut oil significantly raised HDL-C concentrations compared to both butter and olive oil. Butter also significantly raised cholesterol/HDL-C ratio and non-HDL-Cholesterol more than both coconut oil and olive oil but there were no differences between coconut oil and olive oil for changes in cholesterol/HDL-C and non-HDL-C cholesterol.

There were no significant differences in weight or BMI change, change in central adiposity as measured by waist circumference or percent body fat. There were also no significant differences in change in fasting glucose, or systolic and diastolic blood pressure among the three different fat interventions. In pairwise comparison, coconut oil significantly lowered C-Reactive Protein compared to olive oil but there were no significant differences between coconut oil and butter for C-Reactive Protein.

The results were somewhat surprising for a number of reasons. Coconut oil is predominantly (approximately 90%) saturated fat which is generally held to have an adverse effect on blood lipids by increasing blood LDL-C concentrations. However, the saturated fatty acid profiles of different dietary fats vary substantially; coconut oil is predominantly (around 48%) lauric acid (12:0) compared to butter (66% saturated fat) which is about 40% palmitic (16:0) and stearic (18:0) acids, leading to suggestions that coconut oil may not have the same health effects as other foods high in saturated fat[9]. Nevertheless, though reviews on coconut oil and cardiovascular disease risk factors have concluded that the evidence of an association between coconut oil consumption and blood lipids or cardiovascular risk was mostly poor quality[9], trials have generally reported that coconut oil consumption raises LDL-C in comparison to polyunsaturated oil such as safflower oil, though not as much in comparison to butter[10, 11].

For peer review only - http://bmjopen.bmj.com/site/about/guidelines.xhtml

Based on 3 randomized crossover trials of good scientific quality, one trial reported butter increased LDL-C more than coconut oil which raised LDL-C more compared to safflower oil[10]; a second that coconut oil raised LDL-C more than beef fat which raised LDL-C more than safflower oil[23], and a third reported that coconut oil raised LDL-C more than palm oil which raised LDL-C more than olive oil[24]. The current study observed that butter raised LDL-C more than coconut oil but that coconut oil did not differ from olive oil. Two studies showed higher HDL-C with coconut oil compared with other fats whether beef fat, safflower oil or olive oil[23, 24]. Thus far, the current results are consistent with previous studies indicating that butter raises LDL-C more than coconut oil, and also that coconut oil also raises HDL-C. However, the present study is an exception in not finding any increase in LDL-C compared to an unsaturated oil, in this case, olive oil.

This is the largest trial reported to date on coconut oil and lipids apart from a recent study of 200 individuals with established coronary heart disease comparing coconut oil with sunflower oil over 2 years that reported no differences in blood lipids but virtually all the participants were on statin therapy[25] which makes findings difficult to interpret.

Direct comparisons between studies are problematic because of different oils used; we used extra virgin olive oil as a comparison group rather than a polyunsaturated oil such as safflower or sunflower oil, for feasibility reasons of likely participant compliance with the requirement for 50g intake daily. The PREDIMED study reported no significant difference in change in LDL–C or total cholesterol but significant lowering of the cholesterol/HDL-C ratio in the Mediterranean diet supplemented with extra virgin olive oil compared to a low fat diet[2, 13]. A recent review reported that high phenolic olive oil does not modify the lipid profile compared to its low phenolic counterpart[14] though other studies have reported that extra virgin olive oil decreases LDL-C directly measured as concentrations of apoB-100 and the total number of LDL particles as assessed by NMR spectroscopy[26, 27]. We therefore expected coconut oil would raise LDL-C compared to olive oil, but in the current study we observed no evidence of an overall average increase in LDL-C in individuals allocated either to the coconut oil or olive oil intervention.

For peer review only - http://bmjopen.bmj.com/site/about/guidelines.xhtml

Lack of compliance with consuming the dietary fat would lead to no differences between groups and hence explain the lack of differences in LDL-C between coconut oil and olive oil groups. However, in this group of volunteers, reported compliance was high and did not differ between groups; in addition, those in the coconut oil group had significantly greater increases in HDL-C compared to those allocated to olive oil or butter so lack of compliance is unlikely to be an explanation.

The predominant fatty acid in coconut oil, lauric acid(C12:0) as well as myristic acid(C14:0) are medium chain fatty acids that are rapidly absorbed, taken up by the liver and oxidized to increase energy expenditure which is a possible explanation for why coconut oil may have different effects compared to other saturated fats[28]. It is also possible that differences could be attributed to the use of extra virgin preparations of coconut oil rather than standard coconut oil; different methods of preparation such as the chilling method for virgin coconut oil compared to refined, bleached and deodorized coconut oil may influence phenolic compounds and antioxidant activity[29] thus, processing of oils changes their composition, biological properties and consequent potential metabolic effects. The variations in possible health effects resulting from variations in processing of different fats is well documented in the large literature on hydrogenation of polyunsaturated oils to make solid margarines which may increase harmful trans- fats[30]. In this context it is notable that the major trial (PREDIMED) reporting reduction in cardiovascular risk with a Mediterranean diet used extra virgin olive oil[2], while other studies which reported null findings with olive oil may not have always specified the product used[14].

There was no evidence of difference between groups in mean weight, BMI, percent body fat, or central adiposity at the end of this trial; however, these were secondary endpoints for which the trial was not specifically powered. Nevertheless the estimated 95% CI around mean weight differences at the end for the trial were not large. The participants were asked to consume 50g of fat or oils daily. They could do this in the context of their usual diet by substituting for their usual fats, or by consuming these as a supplement. In practice, most participants reported finding it difficult to substitute the different fats or oils for cooking in their usual diet and usually consumed these as a supplement. These fats if taken in addition to their usual diet would have been approximately 450 additional calories daily, which if

For peer review only - http://bmjopen.bmj.com/site/about/guidelines.xhtml

consistently taken four weeks might be expected to be nearly 13,000 additional calories resulting in likely weight gain of 1 to 2kg. This information was provided in the information sheet with the informed consent for participants. While it is possible that participants may have consciously changed behaviours to maintain body weight such as reducing their other dietary intake because of the additional fat or being more physically active, many participants reported that the high fat diet resulted in feeling full and eating less.

It is also possible that even though this was a randomized trial, in an unblinded study, participants may have changed behaviours differentially in the different intervention groups resulting in differences in lipids or lack of differences in weight observed rather than being attributed to the dietary fat interventions. The majority of the participants reported no change in usual physical activity though slightly more participants in the coconut oil and butter groups reported increasing usual physical activity (14% and 15% respectively) compared to 4% in the olive oil group. Nevertheless exclusion of all individuals reporting increased usual physical activity from the analyses did not change the findings. Dietary factors apart from fat most likely to influence HDL-C, total alcohol intake or change in alcohol intake, did not differ significantly between intervention groups and in fact alcohol intake decreased slightly during the trial which would not explain any increases in HDL-C observed. There is therefore no evidence to suggest that differences in lipids, or lack of differences in weight change were likely to be attributed to differential changes in behaviour.

The main strengths of this study are the randomized design with high completion rate (91/94 individuals returned to follow up) and self-reported dietary compliance (nearly 90% participants with over 75% adherence) over four weeks. This is also larger than most trials reported with the exception of the trial in India in individuals with heart disease most of whom were taking statins[25]. The current trial by contrast, was conducted in individuals in the general population.

This trial has limitations. It was a short term trial of four weeks intervention so we are unable to know what would have happened if the intervention had continued for a longer period. Moreover, the current findings only apply to the intermediate metabolic (lipid) risk markers and cannot be extended to findings for clinical endpoints.

For peer review only - http://bmjopen.bmj.com/site/about/guidelines.xhtml

It was designed as a pragmatic trial in free living individuals rather than a controlled metabolic ward trial such that individuals were asked only to consume the 50g of allocated fat or oil daily. We made no attempt to control other aspects of their usual diet in particular, total energy intake. Individuals may have changed their behaviours in different ways to accommodate this additional fat, whether by modifying other aspects of their diet for instance, increasing foods such as bread and potatoes or salads to eat with the fats, or consciously reducing other food intake or changing physical activity patterns to control energy balance. Nevertheless, this trial is more reflective of real life situations.

While self-reported compliance was high, this was subjective and we did not measure the blood fatty acid profile in participants following the intervention for an objective biomarker of compliance. Nevertheless, we did observe differential changes in blood lipids during the intervention.

We did not have a non–additional fat intervention as a comparison group, nor a comparison group with polyunsaturated oils. This was for reasons of feasibility and practicality as it would have added substantially to the numbers (another 30 for an additional intervention arm) and we were also uncertain as to compliance with consumption of 50g of polyunsaturated oil daily in volunteers. We therefore used extra virgin olive oil as a comparison group as that has been generally reported in trials not to increase LDL-C. While the dose of saturated fat of 50g daily was substantial enough to raise LDL-C by levels estimated from previous metabolic ward studies, it was within a feasible daily consumption range.

The generalisability of the findings to the wider population is also unclear. The volunteers were clearly highly selected to be willing to participate in such a study, and also likely to be healthier than the general population, as for ethical reasons we excluded those with known prevalent cardiovascular disease, cancer or diabetes and also those on any lipid lowering medication or other contraindications to a high fat diet. Nevertheless, it is unlikely that the effect of these dietary fats in this group of individuals recruited from the general population would be biologically different from the general population.

For peer review only

For peer review only - http://bmjopen.bmj.com/site/about/guidelines.xhtml

Implications

We focussed on LDL-Cholesterol for the primary endpoint as the causal relationship between LDL-C concentrations and coronary heart disease risk is well established, with about a 15% increase in coronary heart disease risk per 1 mmol/L increase in LDL-C concentrations, and reduction of LDL-C cholesterol lowers coronary heart disease risk[31]. Increase in LDL-C concentrations has been the main mechanism through which dietary saturated fat is believed to increase heart disease risk, though other pathways have been postulated. However, it is notable that some Mediterranean diet interventions such as the Lyon heart stud (alpha linolenic acid)[32] or PREDIMED (extra virgin olive oil) [2] which have been reported to reduce cardiovascular risk in secondary and primary prevention may have effects through other pathways such as inflammation or endothelial function[33, 34]. Whatever the mechanisms, the evidence from prospective studies is consistent and strong that substitution of saturated fats by unsaturated fats is beneficial for cardiovascular risk[35].

In this trial the difference of 0.33mmol/L in LDL-C on butter compared to olive oil is consistent with previous studies[36]. We observed no differences in LDL-C on coconut oil compared to olive oil in this short term study. We also observed no differences among the various fats for a limited range of cardiovascular disease risk factors including fasting glucose, blood pressure and anthropometric measures.

The results of this study indicate that two different dietary fats(coconut oil and butter)which are predominantly saturated fats, appear to have different effects on blood lipids compared to olive oil, a predominantly monounsaturated fat. The effects of different dietary fats on lipid profiles, metabolic markers and health outcomes may vary not just according to the general classification of their main component fatty acids as saturated or unsaturated but possibly according to different profiles in individual fatty acids, processing methods, as well as the foods in which they are consumed or dietary patterns. There is increasing evidence that associations of saturated fatty acids with health outcomes may vary according to whether they are odd or even chain saturated fatty acids, or their chain length[37-39]. Indeed, while overall the evidence indicates the substitution of dietary saturated fats with polyunsaturated fats is beneficial for coronary heart disease risk[40] heterogeneity in findings from observational

For peer review only - http://bmjopen.bmj.com/site/about/guidelines.xhtml

studies and trials may reflect different dietary sources of fats[4, 41] As the summary from Joint FAO/WHO 2008 Expert Consultation on Fats and Fatty Acids in Human Nutrition comments: *"There are inherent limitations with the convention of grouping fatty acids based only on number of double bonds….major groups of fatty acids are associated with different health effects…..individual fatty acids within each broad classification may have unique biological properties or effects…. Intakes of individual fatty acids differ across world depending on predominant food sources of total fats and oils."* The associations with health endpoints may well vary depending on the food sources.

In this trial, extra virgin coconut oil was similar to olive oil and did not raise LDL-C in comparison with butter. The current short-term trial on an intermediate cardiovascular disease risk factor, LDL-C, does not provide evidence to modify existing prudent recommendations to reduce saturated fat in the diet as emphasized in most consensus recommendations[8, 12] and dietary guidelines should be based on a range of criteria[42]. However, the findings highlight the need for further elucidation of the more nuanced relationships between different dietary fats and health. There is increasing evidence that to understand the relationship between diet and health, we need to go beyond simplistic associations between individual nutrients and health outcomes and examine foods and dietary patterns as a whole. In particular, present day diets with high intakes of processed foods now incorporate many fats and oils such as soya bean oil, palm oil and coconut oil which have not been previously widely used in Western societies and not well studied. The relationships between different dietary fats, particularly some of the now more commonly used fats, and health endpoints such as cardiovascular disease events need to be better established.

For peer review only - http://bmjopen.bmj.com/site/about/guidelines.xhtml

Funding statement

This work was supported by the British Broadcasting Corporation, a National Institute of Health Research Senior Investigator Award to KTK and core MRC Epidemiology support (MC UU 12015/5).

Acknowledgements

This study was conducted in collaboration with the British Broadcasting Corporation (BBC) which provided support for the recruitment of participants, running of the community assessment clinic, and biochemistry measurements for lipids. Other costs were supported by the University of Cambridge through a National Institute of Health Research Senior Investigator Award to Kay-Tee Khaw. Nita G Forouhi acknowledge core MRC Epidemiology Support (MC UU 12015/5). We thank Keith Burling and Peter Barker from the Core Biochemical Assay Laboratory, CBAL in Cambridge for the laboratory assays, Shrikant Bangdiwala, University of North Carolina for conducting the computer generated random allocation of participants to the interventions, Timothy Key and colleagues at Oxford University for the use of the DietWebQ, and Nichola Dalzell and Shabina Hayat, Department of Public Health and Primary Care, and Eirini Trichia, Richard Powell and Meriel Smith,MRC Epidemiology Unit, University of Cambridge for logistical support. We thank the Cambridge Yoga Centre which hosted the assessment sessions for participants in June and July 2017. Most of all, we thank the participants from the general community who generously volunteered to take part in this trial; this study would not have been possible without their efforts and we are most grateful to them.

The BBC and the University of Cambridge collaborated in the design and conduct of the study, data collection and management of the study. The University of Cambridge investigators were solely responsible for the analysis and interpretation of the data, and preparation of the manuscript. The BBC producer coordinating the study (LF) is a co author who has reviewed and approved the manuscript but the BBC has otherwise had no editorial role in the manuscript.

Competing interest statement

For peer review only - http://bmjopen.bmj.com/site/about/guidelines.xhtml

All authors have completed the Unified Competing Interest form and declare no support from any organisation for the submitted work except as listed in the acknowledgements;; no financial relationships with any organisations that might have an interest in the submitted work in the previous three years and, no other relationships or activities that could appear to have influenced the submitted work

Conflicts of interest

None

Copyright

The Corresponding Author has the right to grant on behalf of all authors and does grant on behalf of all authors, a worldwide licence to the Publishers and its licensees in perpetuity, in all forms, formats and media (whether known now or created in the future), to i) publish, reproduce, distribute, display and store the Contribution, ii) translate the Contribution into other languages, create adaptations, reprints, include within collections and create summaries, extracts and/or, abstracts of the Contribution, iii) create any other derivative work(s) based on the Contribution, iv) to exploit all subsidiary rights in the Contribution, v) the inclusion of electronic links from the Contribution to third party material whereever it may be located; and, vi) license any third party to do any or all of the above

Ethics and Consent

Ethics approval was given by the University of Cambridge Human Biology Research Ethics Committee Application no. HBREC.2017.05. All participants gave signed informed consent. Clinical Trials registration April 2017 NCT03105947 USNIH Clinical Trials.gov

Contributors and transparency declaration

Kay-Tee Khaw had full access to all of the data in the study and takes responsibility for the integrity of the data and the accuracy of the data analysis. The lead author and guarantor Khaw affirms that the manuscript is an honest, accurate, and transparent account of the study being reported; that no important aspects of the study have been omitted; and that any discrepancies from the study as planned have been explained.

Study concept and design: Khaw, Forouhi, Finikarides

For peer review only - http://bmjopen.bmj.com/site/about/guidelines.xhtml

Acquisition of data: Khaw, Forouhi, Finikarides, Afzal, Luben, Lentjes

Analysis and interpretation of the data: Sharp, Khaw, Forouhi

Drafting of the manuscript: Khaw

Critical revision of the manuscript for important intellectual content: Forouhi, Sharp, Afzal, Finkarides, Luben, Lentjes

Obtaining funding: Khaw, Finikarides, Forouhi

Administrative, technical or material support: Khaw, Forouhi, Finikarides, Afzal, Luben, Sharp, Lentjes

For peer review only

Data sharing statement

Data are available. Please contact corresponding author.

For peer review only - http://bmjopen.bmj.com/site/about/guidelines.xhtml

Reference List

1. Howard BV, Van HL, Hsia J et al. Low-fat dietary pattern and risk of cardiovascular disease: the Women's Health Initiative Randomized Controlled Dietary Modification Trial. JAMA 2006;295(6):655-666.
2. Estruch R, Ros E, Salas-Salvado J et al. Primary prevention of cardiovascular disease with a Mediterranean diet. N Engl J Med 2013;368(14):1279-1290.
3. Chowdhury R, Warnakula S, Kunutsor S et al. Association of dietary, circulating, and supplement fatty acids with coronary risk: a systematic review and meta-analysis. Ann Intern Med 2014;160(6):398-406.
4. Hooper L, Martin N, Abdelhamid A, Davey SG. Reduction in saturated fat intake for cardiovascular disease. Cochrane Database Syst Rev 2015;(6):CD011737.
5. Alexander DD, Bylsma LC, Vargas AJ et al. Dairy consumption and CVD: a systematic review and meta-analysis. Br J Nutr 2016;115(4):737-750.
6. Liang J, Zhou Q, Kwame AW, Su Y, Zhang Z. Biomarkers of dairy fat intake and risk of cardiovascular disease: a systematic review and meta analysis of prospective studies. Crit Rev Food Sci Nutr 2016;0.
7. Pimpin L, Wu JH, Haskelberg H, Del GL, Mozaffarian D. Is Butter Back? A Systematic Review and Meta-Analysis of Butter Consumption and Risk of Cardiovascular Disease, Diabetes, and Total Mortality. PLoS One 2016;11(6):e0158118.
8. Department of Health and Human Services U, Department of Agriculture (US). 2015-2020 Dietary Guidelines for Americans. 8th Edition. 2015
9. Eyres L, Eyres MF, Chisholm A, Brown RC. Coconut oil consumption and cardiovascular risk factors in humans. Nutr Rev 2016;74(4):267-280.
10. Cox C, Mann J, Sutherland W, Chisholm A, Skeaff M. Effects of coconut oil, butter, and safflower oil on lipids and lipoproteins in persons with moderately elevated cholesterol levels. J Lipid Res 1995;36(8):1787-1795.
11. Cox C, Sutherland W, Mann J, de JS, Chisholm A, Skeaff M. Effects of dietary coconut oil, butter and safflower oil on plasma lipids, lipoproteins and lathosterol levels. Eur J Clin Nutr 1998;52(9):650-654.
12. Sacks FM, Lichtenstein AH, Wu JHY et al. Dietary Fats and Cardiovascular Disease: A Presidential Advisory From the American Heart Association. Circulation 2017;136(3):e1-e23.
13. Estruch R, Martinez-Gonzalez MA, Corella D et al. Effects of a Mediterranean-style diet on cardiovascular risk factors: a randomized trial. Ann Intern Med 2006;145(1):1-11.
14. Hohmann CD, Cramer H, Michalsen A et al. Effects of high phenolic olive oil on cardiovascular risk factors: A systematic review and meta-analysis. Phytomedicine 2015;22(6):631-640.

For peer review only - http://bmjopen.bmj.com/site/about/guidelines.xhtml

15. Liu B, Young H, Crowe FL et al. Development and evaluation of the Oxford WebQ, a low-cost, web-based method for assessment of previous 24 h dietary intakes in large-scale prospective studies. Public Health Nutr 2011;14(11):1998-2005.

16. HAGEN JH, HAGEN PB. An enzymic method for the estimation of glycerol in blood and its use to determine the effect of noradrenaline on the concentration of glycerol in blood. Can J Biochem Physiol 1962;40:1129-1139.

17. Rautela GS, Liedtke RJ. Automated enzymic measurement of total cholesterol in serum. Clin Chem 1978;24(1):108-114.

18. Nauck M, Warnick GR, Rifai N. Methods for measurement of LDL-cholesterol: a critical assessment of direct measurement by homogeneous assays versus calculation. Clin Chem 2002;48(2):236-254.

19. Canoy D, Wareham N, Luben R et al. Serum lipid concentration in relation to anthropometric indices of central and peripheral fat distribution in 20,021 British men and women: results from the EPIC-Norfolk population-based cohort study. Atherosclerosis 2006;189(2):420-427.

20. Forouhi NG, Menon RK, Sharp SJ et al. Effects of vitamin D2 or D3 supplementation on glycaemic control and cardiometabolic risk among people at risk of type 2 diabetes: results of a randomized double-blind placebo-controlled trial. Diabetes Obes Metab 2016;18(4):392-400.

21. Borm GF, Fransen J, Lemmens WA. A simple sample size formula for analysis of covariance in randomized clinical trials. J Clin Epidemiol 2007;60(12):1234-1238.

22. Clarke R, Frost C, Collins R, Appleby P, Peto R. Dietary lipids and blood cholesterol: quantitative meta-analysis of metabolic ward studies. BMJ 1997;314(7074):112-117.

23. Reiser R, Probstfield JL, Silvers A et al. Plasma lipid and lipoprotein response of humans to beef fat, coconut oil and safflower oil. Am J Clin Nutr 1985;42(2):190-197.

24. Voon PT, Ng TK, Lee VK, Nesaretnam K. Diets high in palmitic acid (16:0), lauric and myristic acids (12:0 + 14:0), or oleic acid (18:1) do not alter postprandial or fasting plasma homocysteine and inflammatory markers in healthy Malaysian adults. Am J Clin Nutr 2011;94(6):1451-1457.

25. Vijayakumar M, Vasudevan DM, Sundaram KR et al. A randomized study of coconut oil versus sunflower oil on cardiovascular risk factors in patients with stable coronary heart disease. Indian Heart J 2016;68(4):498-506.

26. Hernaez A, Fernandez-Castillejo S, Farras M et al. Olive oil polyphenols enhance high-density lipoprotein function in humans: a randomized controlled trial. Arterioscler Thromb Vasc Biol 2014;34(9):2115-2119.

27. Hernaez A, Remaley AT, Farras M et al. Olive Oil Polyphenols Decrease LDL Concentrations and LDL Atherogenicity in Men in a Randomized Controlled Trial. J Nutr 2015;145(8):1692-1697.

28. DeLany JP, Windhauser MM, Champagne CM, Bray GA. Differential oxidation of individual dietary fatty acids in humans. Am J Clin Nutr 2000;72(4):905-911.

29. Marina AM, Man YB, Nazimah SA, Amin I. Antioxidant capacity and phenolic acids of virgin coconut oil. Int J Food Sci Nutr 2009;60 Suppl 2:114-123.

For peer review only - http://bmjopen.bmj.com/site/about/guidelines.xhtml

For peer review only

30. Kummerow FA. The negative effects of hydrogenated trans fats and what to do about them. Atherosclerosis 2009;205(2):458-465.

31. Goodman DS, Hulley SB, Clark LT. Report of the national cholesterol education program expert panel on detection, evaluation, and treatment of high blood cholesterol in adults. Archives of Internal Medicine 1988;148(1):36-69.

32. de LM, Renaud S, Mamelle N et al. Mediterranean alpha-linolenic acid-rich diet in secondary prevention of coronary heart disease. Lancet 1994;343(8911):1454-1459.

33. Casas R, Sacanella E, Urpi-Sarda M et al. The effects of the mediterranean diet on biomarkers of vascular wall inflammation and plaque vulnerability in subjects with high risk for cardiovascular disease. A randomized trial. PLoS One 2014;9(6):e100084.

34. Casas R, Sacanella E, Urpi-Sarda M et al. Long-Term Immunomodulatory Effects of a Mediterranean Diet in Adults at High Risk of Cardiovascular Disease in the PREvencion con DIeta MEDiterranea (PREDIMED) Randomized Controlled Trial. J Nutr 2016;146(9):1684-1693.

35. Li Y, Hruby A, Bernstein AM et al. Saturated Fats Compared With Unsaturated Fats and Sources of Carbohydrates in Relation to Risk of Coronary Heart Disease: A Prospective Cohort Study. J Am Coll Cardiol 2015;66(14):1538-1548.

36. Engel S, Tholstrup T. Butter increased total and LDL cholesterol compared with olive oil but resulted in higher HDL cholesterol compared with a habitual diet. Am J Clin Nutr 2015;102(2):309-315.

37. Khaw KT, Friesen MD, Riboli E, Luben R, Wareham N. Plasma phospholipid fatty acid concentration and incident coronary heart disease in men and women: the EPIC-Norfolk prospective study. PLoS Med 2012;9(7):e1001255.

38. Praagman J, Beulens JW, Alssema M et al. The association between dietary saturated fatty acids and ischemic heart disease depends on the type and source of fatty acid in the European Prospective Investigation into Cancer and Nutrition-Netherlands cohort. Am J Clin Nutr 2016;103(2):356-365.

39. Forouhi NG, Koulman A, Sharp SJ et al. Differences in the prospective association between individual plasma phospholipid saturated fatty acids and incident type 2 diabetes: the EPIC-InterAct case-cohort study. Lancet Diabetes Endocrinol 2014;2(10):810-818.

40. Mozaffarian D, Micha R, Wallace S. Effects on coronary heart disease of increasing polyunsaturated fat in place of saturated fat: a systematic review and meta-analysis of randomized controlled trials. PLoS Med 2010;7(3):e1000252.

41. Ramsden CE, Zamora D, Leelarthaepin B et al. Use of dietary linoleic acid for secondary prevention of coronary heart disease and death: evaluation of recovered data from the Sydney Diet Heart Study and updated meta-analysis. BMJ 2013;346:e8707.

42. Smit LA, Mozaffarian D, Willett W. Review of fat and fatty acid requirements and criteria for developing dietary guidelines. Ann Nutr Metab 2009;55(1-3):44-55.

For peer review only

For peer review only - http://bmjopen.bmj.com/site/about/guidelines.xhtml

Table 1

Descriptive characteristics at baseline assessment of participants in the COB trial according to allocation (intention to treat)

	Coconut oil N=29		Butter N=33		Olive Oil N=32	
	**Mean**	**(SD)**	**Mean**	**(SD)**	**Mean**	**(SD)**
Age (years)	59.1	(6.1)	61.5	(5.8)	59.1	(6.4)
LDL-Cholesterol (mmol/l)	3.5	(0.9)	3.5	(0.9)	3.7	(1.0)
Total cholesterol (mmol/l)	5.9	(1.0)	5.9	(1.0)	6.0	(0.9)
HDL-Cholesterol (mmol/l)	2.0	(0.5)	1.9	(0.5)	1.8	(0.5)
Cholesterol/HDL ratio	3.2	(0.9)	3.2	(0.8)	3.5	(1.2)
Non HDL-Cholesterol (mmol/l)	3.9	(1.0)	4.0	(0.9)	4.2	(1.1)
Glucose (mmol/l)	5.3	(0.4)	5.4	(0.5)	5.4	(0.5)
Weight (kg)	73.9	(15.1)	70.8	(11.7)	71.1	(14.5)
Waist (cm)	85.4	(11.9)	83.7	(8.1)	86.2	(11.5)
Body fat (%)	29.7	(10.2)	29.2	(9.0)	31.5	(9.6)
Body Mass Index (kg/m2)	25.5	(4.5)	24.8	(3.5)	25.0	(4.5)
Systolic blood pressure (mmHg)	131.4	(18.8)	136.5	(18.8)	133.1	(16.5)
Diastolic blood pressure (mmHg)	79.8	(9.3)	81.0	(12.0)	78.1	(6.7)
DietWebQ intake/day						
Total energy (MJ)	9.00	(3.70)	8.23	(2.17)	9.51	(3.5)
Protein % energy	14.8	(4.4)	16.0	(3.7)	15.7	(3.0)
Carbohydrate % energy	43.6	(8.9)	41.4	(8.7)	42.7	(11.7)
Total fat% energy	37.3	(7.3)	36.7	(8.7)	36.4	(10.3)
Saturated fat% energy	14.1	(3.6)	13.3	(4.4)	13.4	(4.9)
Alcohol % energy	4.2	(5.4)	5.9	(7.5)	5.1	(6.1)
Hours of walking in past week	8.9	(9.5)	10.9	(12.3)	10.1	(8.7)
Hours of cycling in past week	1.8	(2.6)	2.0	(2.5)	2.7	(5.5)
Hours of other physical exercise in past week	3.4	(3.4)	2.3	(4.0)	1.8	(2.6)

For peer review only

For peer review only - http://bmjopen.bmj.com/site/about/guidelines.xhtml

14 December 2017 V3

Table 1 continued Descriptive characteristics at baseline assessment of participants in the COB trial according to allocation (intention to treat)

	Coconut oil N=29		Butter N=33		Olive Oil N=32	
	**Median**	**(IQR)**	**Median**	**(IQR)**	**Median**	**(IQR)**
Triglycerides (mmol/l)	0.89	(0.74,1.10)	0.92	(0.70,1.20)	0.94	(0.79,1.31)
C-Reactive Protein (mg/l)	1.04	(0.47,2.15)	1.08	(0.64,2.13)	1.13	(0.58,2.67)
	**%**	**(N)**	**%**	**(N)**	**%**	**(N)**
Sex						
Men	37.9	(11)	33.3	(11)	28.1	(9)
Women	62.1	(18)	66.7	(22)	71.9	(23)
Ethnicity						
White	96.6	(28)	97.0	(32)	93.8	(30)
Non-white	3.4	(1)	3.0	(1)	3.1	(1)
Smoking status						
Never	58.6	(17)	66.7	(22)	68.8	(22)
Former	34.5	(10)	33.3	(11)	25.0	(8)
Current	6.9	(2)	0.0	(0)	6.3	(2)
Alcohol consumption in past year						
Never or once per month	20.7	(6)	30.3	(10)	28.1	(9)
1-4 times per week	72.4	(21)	48.5	(16)	59.4	(19)
Almost every day or every day	6.9	(2)	21.2	(7)	12.5	(4)
Highest level of education						
School to age 16	13.8	(4)	12.1	(4)	15.6	(5)
School to age 18	27.6	(8)	9.1	(3)	9.4	(3)
University	58.6	(17)	78.8	(26)	75.0	(24)
Currently in paid job						
No	20.7	(6)	45.5	(15)	25.0	(8)
Yes	75.9	(22)	54.5	(18)	75.0	(24)

IQR: Interquartile range

For peer review only - http://bmjopen.bmj.com/site/about/guidelines.xhtml

14 December 2017 V3

Table 2

Mean change in variables between baseline and follow up after dietary interventions and pairwise comparisons between fats in 91 participants

	Change from Baseline				Pairwise comparisons		
	Coconut oil N=28 Mean (SD)	Butter N=33 Mean (SD)	Olive Oil N=30 Mean (SD)	P value Comparison Between groups	Coconut oil vs olive oil Difference (95% CI)	Butter vs Coconut oil Difference (95% CI)	Butter vs olive oil Difference (95% CI)
LDL-Cholesterol mmol/L	-0.09 (0.49)	0.33 (0.48)	-0.06 (0.39)	<0.001	-0.04 (-0.27, 0.19)	0.42 (0.19,0.65)	0.38 (0.16,0.60)
Total cholesterol mmol/L	0.22 (0.55)	0.42 (0.59)	0.03 (0.43)	0.022	0.19 (-0.08,0.46)	0.19(-0.08,0.45)	0.38 (0.11,0.64)
HDL-Cholesterol mmol/L	0.28 (0.29)	0.09 (0.27)	0.10 (0.15)	0.009	0.16 (0.03,0.28)	-0.18 (-0.30,-0.06)	-0.02 (-0.14,0.09)
Triglycerides mmol/L	0.07 (0.58)	-0.001 (0.36)	-0.03 (0.27)	0.65	0.10 (-0.12,0.32)	-0.08 (-0.29,0.13)	0.02 (-0.19,0.23)
Cholesterol/HDL ratio	-0.26 (0.36)	0.10 (0.41)	-0.13 (0.32)	<0.001	-0.14 (-0.33,0.05)	0.36 (0.18,0.54)	0.22 (0.04,0.40)
Non HDL-Cholesterol mmol/L	-0.06 (0.44)	0.33 (0.51)	-0.07 (0.42)	0.001	0.002 (-0.23,0.24)	0.39 (0.16,0.62)	0.39 (0.16,0.62)
Glucose mmol/L	-0.05 (0.49)	0.02 (0.48)	-0.06 (0.49)	0.68	0.01 (-0.23,0.25)	0.08(-0.15,0.32)	0.09 (-0.14,0.33)
C-Reactive Protein mg/L	-0.31 (1.09)	-0.04 (0.93)	0.23 (1.40)	0.11	-0.58 (-1.12,-0.04)	0.29 (-0.24,0.82)	-0.29 (-0.80,0.23)
Weight Kg	0.27 (0.77)	0.04 (1.00)	-0.04 (0.84)	0.42	0.30 (-0.16, 0.76)	-0.22 (-0.67, 0.23)	0.08 (-0.36, 0.52)
Waist cm	1.29 (3.31)	0.26 (3.43)	0.59 (3.25)	0.52	0.71 (-1.00,2.42)	-0.95 (-2.63,0.72)	-0.24 (-1.89, 1.41)
Body fat %	0.24 (1.03)	0.34 (1.31)	0.13 (1.30)	0.82	0.09 (-0.54,0.73)	0.10 (-0.52,0.72)	0.19 (-0.42, 0.81)
Body Mass Index kg/m2	0.09 (0.27)	0.02 (0.35)	-0.01 (0.29)	0.13	0.10 (-0.06,0.26)	-0.07 (-0.22,0.09)	0.03 (-0.12, 0.18)
Systolic blood pressure mmHg	0.18 (11.46)	-3.79 (11.11)	-3.67 (8.23)	0.29	3.91 (-1.22, 9.04)	-3.22 (-8.26, 1.82)	0.69 (-4.26,5.64)
Diastolic blood pressure mmHg	-2.02 (5.71)	-1.33 (6.24)	-0.45 (8.48)	0.81	-0.73 (-3.88, 2.42)	0.99 (-2.08,4.05)	0.26 (-2.78,3.30)

For peer review only - http://bmjopen.bmj.com/site/about/guidelines.xhtml

14 December 2017 V3

Table 3

Baseline and follow up dietary intake by allocation to coconut oil, butter or olive oil* estimated using 24 hour DietWebQ

DietWebQ intake/day	Coconut oil	Butter	Olive oil
Baseline prior to start of intervention	N=27	n=33	n=32
Energy MJ/d	9.0 (3.7)	8.2 (2.2)	9.5 (3.5)
Total fat g/d	94 (47)	81 (26)	98 (50)
Protein g/d	74 (29)	75 (19)	87 (34)
Carbohydrate g/d	238 (95)	215 (75)	243(95)
Alcohol g/d	16(22)	17 (23)	18(22)
At four weeks of intervention	n=24	n=32	n=27
Energy MJ/d	9.6 (3.2)	8.6 (2.4)	9.6 (3.1_
Total fat g/d	127 (47)	94 (37)	138 (38)
Protein g/d	71 (25)	77 (29)	78 (31)
Carbohydrate g/d	215 (84)	214 (64)	197 (101)
Alcohol g/d	9 (15)	13(15)	8(18)
Change from baseline	n=24	n=32	n=27
Energy MJ/d	0.3 (2.9)	0.5 (2.0)	-0.4 (2.8)
Total fat g/d	29 (43)	14 (36)	28 (40)
Protein g/d	-7 (33)	3 (30)	-12 (26)
Carbohydrate g/d	-31 (74)	4 (69)	-55(81)
Alcohol g/d	-8 (22)	-5(23)	-11 (27)

*numbers do not total 94 as not all participants completed the baseline and follow up DietWebQ

For peer review only

For peer review only - http://bmjopen.bmj.com/site/about/guidelines.xhtml

14 December 2017 V3

Table 4 supplemental

Mean change in variables between baseline and follow up after dietary interventions in 71 participants who reported no change in physical activity during the trial

	Change from Baseline			
	Coconut oil N=22 Mean (SD)	Butter N=24 Mean (SD)	Olive Oil N=25 Mean (SD)	P value Comparison Between groups
LDL-Cholesterol mmol/L	-0.10 (0.50)	0.20 (0.53)	-0.04 (0.35)	0.01
Total cholesterol mmol/L	0.19 (0.59)	0.38 (0.63)	0.07 (0.37)	0.13
HDL-Cholesterol mmol/L	0.31 (0.29)	0.10 (0.26)	0.12 (0.16)	0.001
Triglycerides mmol/L	-0.02 (0.46)	-0.01 (0.42)	-0.04 (0.23)	0.97
Cholesterol/HDL ratio	-0.30(0.35)	0.07 (0.44)	-0.13 (0.30)	0.004
Non HDL-Cholesterol mmol/L	-0.11 (0.44)	0.28 (0.56)	-0.06 (0.36)	0.008
Glucose mmol/L	-0.12 (0.49)	-0.02 (0.52)	-0.08 (0.51)	0.80
C-Reactive Protein mg/L	-0.30 (1.18)	-0.13 (0.86)	0.04 (1.00)	0.51
Weight Kg	0.13 (0.62)	0.07 (1.06)	-0.02 (0.76)	0.83
Waist cm	1.47 (3.35)	0.67 (3.48)	0.81 (3.48)	0.70
Body fat %	0.34 (1.11)	0.23 (1.37)	0.81 (1.37)	0.71
Body Mass Index kg/m2	0.04 (0.22)	0.03 (0.37)	0.00 (0.26)	0.85
Systolic blood pressure mm Hg	-3.1 (8.9)	-5.1 (11.3)	-2.4 (7.8)	0.60
Diastolic blood pressure mm Hg	-2.4 (5.6)	-2.0 (6.6)	0.8 (8.4)	0.24

For peer review only - http://bmjopen.bmj.com/site/about/guidelines.xhtml

14 December 2017 V3

Legends for figures

Figure 1

Recruitment and Flow diagram (CONSORT) for Coconut Oil, Olive Oil or Butter Trial

Figure 2

Difference (95% CI) in the primary outcome (LDL cholesterol) between each pair of randomised groups, reported in units of baseline SD. Mean (SD) change from baseline is also presented for each group in mmol/l. COB study, Intention to Treat population n=91

Figure 3

Difference (95% CI) in secondary outcomes comparing Butter vs Coconut Oil groups, reported in units of baseline SD. Mean (SD) change from baseline is also presented for each group in the natural units of the outcome. COB study, Intention to Treat population n=91. For HDL cholesterol, sign of difference and 95% CI is the opposite of that reported in Table 2, on the assumption that higher HDL is better, so the negative estimated difference (Butter vs Coconut) reported in Table 2 is presented on the side of the graph which favours the Coconut group.

Figure 4

Difference (95% CI) in secondary outcomes comparing Coconut Oil vs Olive Oil groups, reported in units of baseline SD. Mean (SD) change from baseline is also presented for each group in the natural units of the outcome. COB study, Intention to Treat population n=91. For HDL cholesterol, sign of difference and 95% CI is the opposite of that reported in Table 2, on the assumption that higher HDL is better, so the positive estimated difference (Coconut vs Olive) reported in Table 2 is presented on the side of the graph which favours the Coconut group.

Figure 5

Difference (95% CI) in secondary outcomes comparing Butter vs Olive Oil groups, reported in units of baseline SD. Mean (SD) change from baseline is also presented for each group in the natural units of the outcome. COB study, Intention to Treat population n=91. For HDL cholesterol, sign of difference and 95% CI is the opposite of that reported in Table 2, on the assumption that higher HDL is better, so the negative estimated difference (Butter vs Olive) reported in Table 2 is presented on the side of the graph which favours the Olive group.

For peer review only

For peer review only - http://bmjopen.bmj.com/site/about/guidelines.xhtml

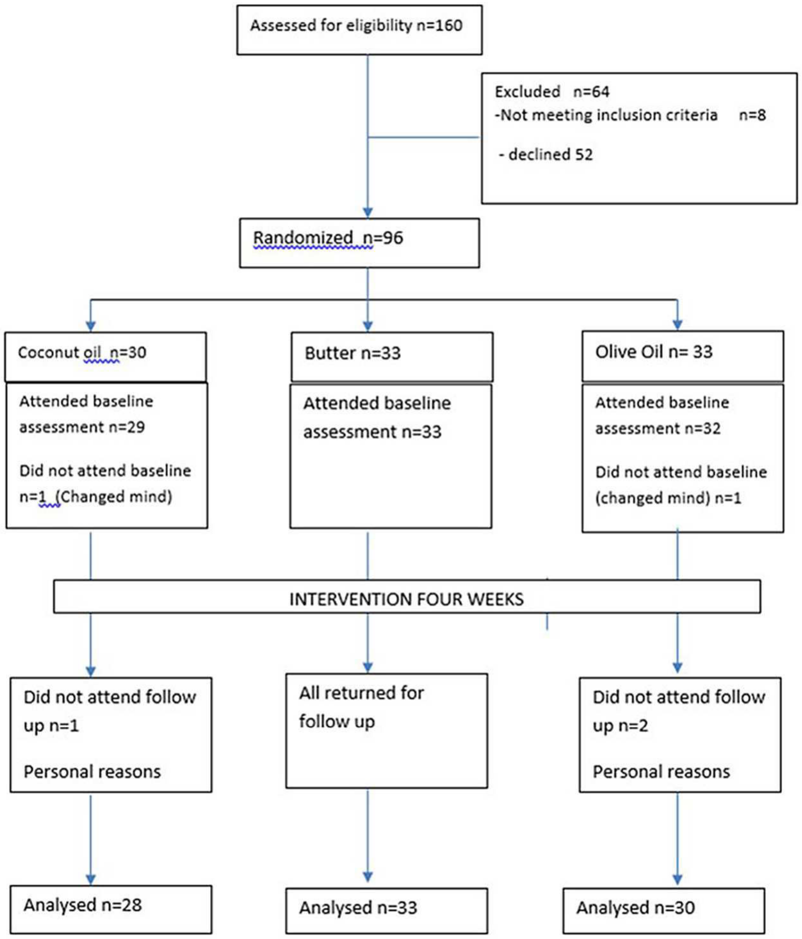

Recruitment and Flow diagram (CONSORT) for Coconut Oil, Olive Oil or Butter Trial

137x173mm (300 x 300 DPI)

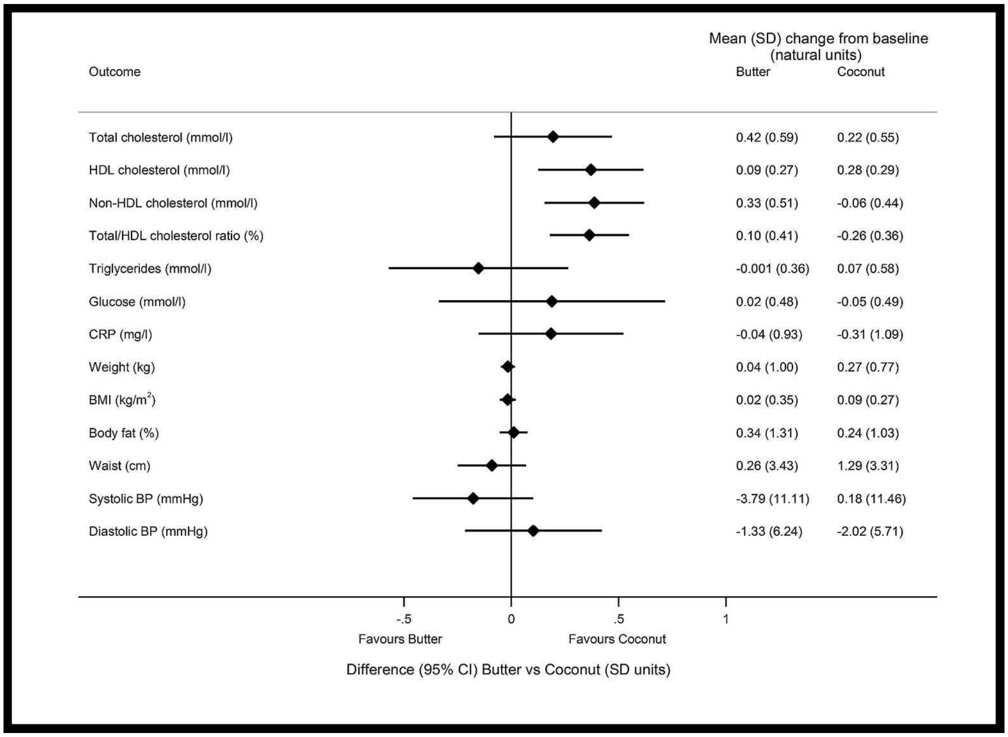

Difference (95% CI) in the primary outcome (LDL cholesterol) between each pair of randomised groups, reported in units of baseline SD. Mean (SD) change from baseline is also presented for each group in mmol/l. COB study, Intention to Treat population n=91

189x138mm (300 x 300 DPI)

w only

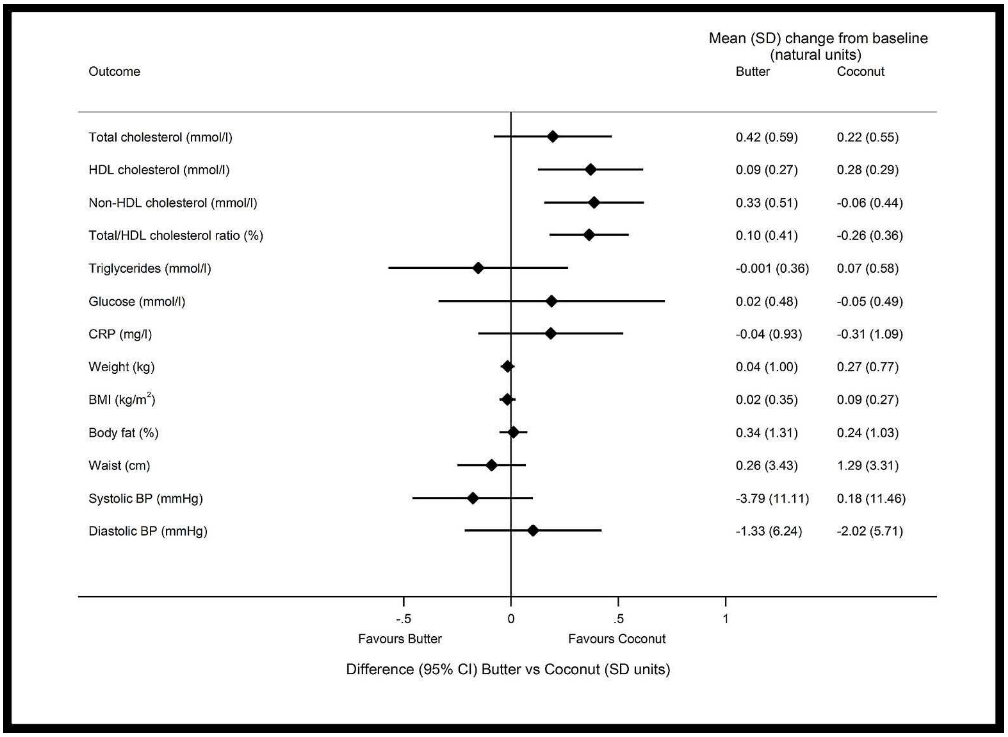

Difference (95% CI) in secondary outcomes comparing Butter vs Coconut Oil groups, reported in units of baseline SD. Mean (SD) change from baseline is also presented for each group in the natural units of the outcome. COB study, Intention to Treat population n=91. For HDL cholesterol, sign of difference and 95% CI is the opposite of that reported in Table 2, on the assumption that higher HDL is better, so the negative estimated difference (Butter vs Coconut) reported in Table 2 is presented on the side of the graph which favours the Coconut group.

189x138mm (300 x 300 DPI)

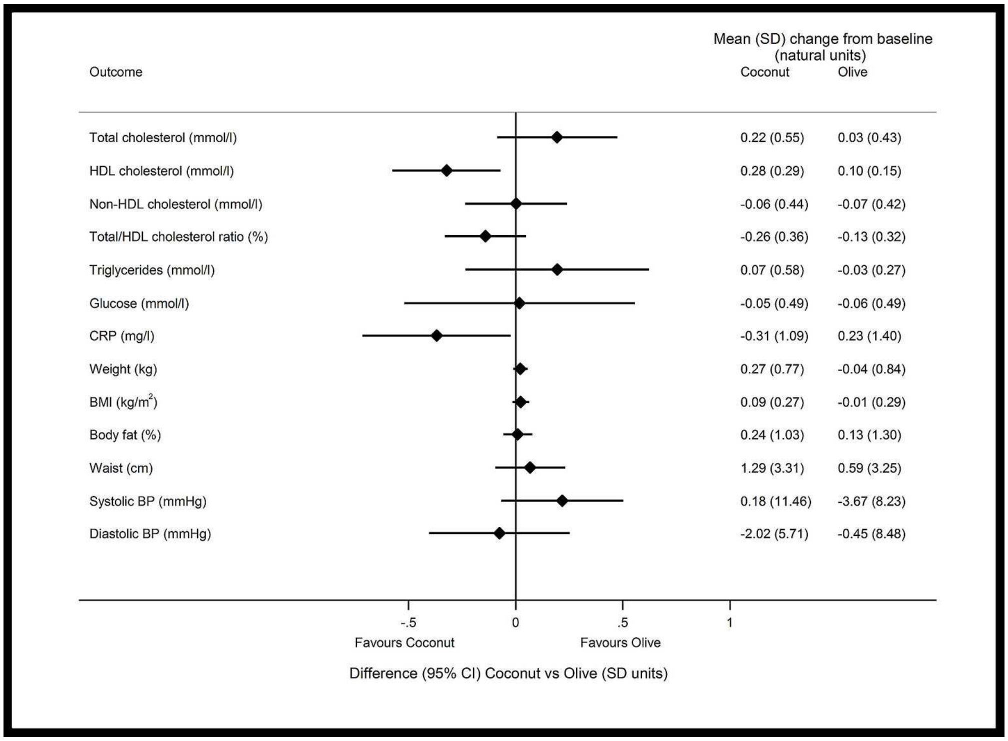

Difference (95% CI) in secondary outcomes comparing Coconut Oil vs Olive Oil groups, reported in units of baseline SD. Mean (SD) change from baseline is also presented for each group in the natural units of the outcome. COB study, Intention to Treat population n=91. For HDL cholesterol, sign of difference and 95% CI is the opposite of that reported in Table 2, on the assumption that higher HDL is better, so the positive estimated difference (Coconut vs Olive) reported in Table 2 is presented on the side of the graph which favours the Coconut group.

189x139mm (300 x 300 DPI)

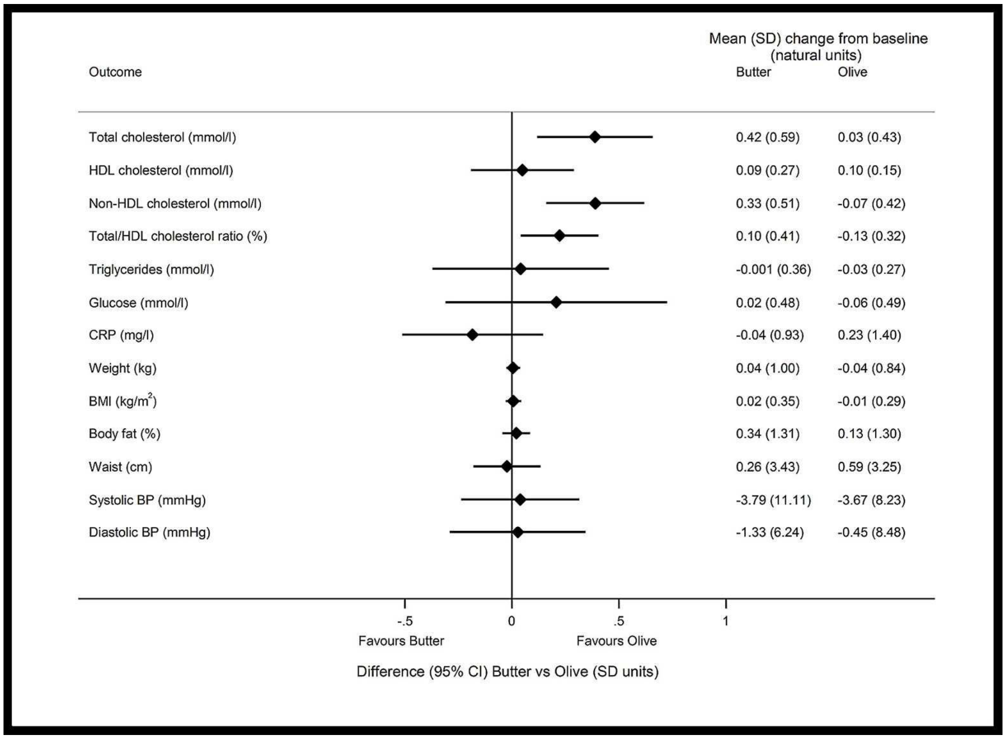

Difference (95% CI) in secondary outcomes comparing Butter vs Olive Oil groups, reported in units of baseline SD. Mean (SD) change from baseline is also presented for each group in the natural units of the outcome. COB study, Intention to Treat population n=91. For HDL cholesterol, sign of difference and 95% CI is the opposite of that reported in Table 2, on the assumption that higher HDL is better, so the negative estimated difference (Butter vs Olive) reported in Table 2 is presented on the side of the graph which favours the Olive group.

150x109mm (300 x 300 DPI)

Appendix 1: Fatty acid composition of fats

Samples of the fats/oils used in the trial were sent for fatty acid composition to West Yorkshire Analytical Services, a UKAS accredited testing service for food composition. The results are tabulated below.

Coconut oil was 94 % saturated fatty acids, of which the main components were lauric acid C12:0 (48%) and myristic acid C14:0 (19%), palmitic acid C16:0 (9%) and caprylic acid C8:0 (9%); and 5% mono unsaturated fat, mainly oleic acid C18:1n9 (5%).

Butter was 66% saturated fatty acids, of which the main components were palmitic acid C16:0 (28%), stearic acid C18:0 (12%), myristic acid C14:0 (11%); 26% monounsaturated fat, mainly oleic acid C18:1n9 (22%); and 3% polyunsaturated fat, linoleic acid C18:2n6 (2%) and alpha-linolenic acid (1%).

Olive oil was 19% saturated fatty acids, mainly palmitic acid C16:0, 15% with stearic acid C18:0 (3%); 68% monounsaturates with the main component being oleic acid C18:1n9 (64%); and 13% polyunsaturates Linoleic acid C18:2n6 (12%).

		Coconut oil	Olive Oil	Butter
		% composition	% composition	% composition
C4:0	Butyric acid	<1	<0.1	2.5
C6:0	Caproic acid	0.7	<0.1	1.9
C8:0	Caprylic acid	8.6	<0.1	1.2
C10:0	Capric acid	6.3	<0.1	2.5
C12:0	Lauric acid	47.6	<0.1	3
C14:0	Myristic acid	18.6	<0.1	10.6
C14:1		<0.1	<0.1	0.9
C15:0		<0.1	<0.1	1.1
C16:0	Palmitic acid	8.6	14.8	28.1
C16:1	Palmitoleic acid	<0.1	1.5	1.4
C17:0		<0.1	<0.1	0.6
C17:1		<0.1	<0.1	0.4
C18.0	Stearic Acid	3.4	3	12.4
C18:1t			<0.1	3.2
C18:1n9	Oleic Acid	5.2	63.5	22.2
C181n7	cis-Vaccenic Acid	<0.1	2.8	0.4

For peer review only - http://bmjopen.bmj.com/site/about/guidelines.xhtml

C18:2tt		<0.1	<0.1	0.5
C18:2ct		<0.1	<0.1	0.1
C18:2tc		<0.1	<0.1	0.2
C18:2n6	Linoleic Acid	0.8	11.9	1.9
C18:3n6	Gamma Linolenic Acid	<0.1	<0.1	<0.1
C18:3n3	Alpha-Linolenic Acid	<0.1	<0.1	0.9
C20:0	Arachidic acid	<0.1	<0.1	0.2
C20:2n6	Eicosadienoic acid	<0.1	<0.1	<0.1
C18:4n3	Stearidonic acid	<0.1	0.2	0.1
C20:1	Paullinic acid	<0.1	<0.1	<0.1
C22:0	Behenic Acid	<0.1	0.2	0.1
C22:1n9	Erucic Acid	<0.1	<0.1	0.1
C22:2	Docosadienoic acid	<0.1	0.6	<0.1
C24:0	Lignoceric acid	<0.1	<0.1	<0.1
	Saturates	93.9	18.6	66.2
	Monounsaturates	5.2	68	26.1
	Polyunsaturates	0.7	13.5	3.4
	Transesters	<0.1	<0.1	4.2

For peer review only

For peer review only - http://bmjopen.bmj.com/site/about/guidelines.xhtml

CONSORT 2010 checklist of information to include when reporting a randomised trial*

Section/Topic	Item No	Checklist item	Reported on page No
**Title and abstract**			
	1a	Identification as a randomised trial in the title	1
	1b	Structured summary of trial design, methods, results, and conclusions (for specific guidance see CONSORT for abstracts)	2,3
**Introduction**			
Background and objectives	2a	Scientific background and explanation of rationale	5,6
	2b	Specific objectives or hypotheses	6
**Methods**			
Trial design	3a	Description of trial design (such as parallel, factorial) including allocation ratio	7,8
	3b	Important changes to methods after trial commencement (such as eligibility criteria), with reasons	N/A
Participants	4a	Eligibility criteria for participants	7
	4b	Settings and locations where the data were collected	7,8
Interventions	5	The interventions for each group with sufficient details to allow replication, including how and when they were actually administered	7,8
Outcomes	6a	Completely defined pre-specified primary and secondary outcome measures, including how and when they were assessed	8,9
	6b	Any changes to trial outcomes after the trial commenced, with reasons	N/A
Sample size	7a	How sample size was determined	9,10
	7b	When applicable, explanation of any interim analyses and stopping guidelines	N/A
Randomisation:			
Sequence generation	8a	Method used to generate the random allocation sequence	7
	8b	Type of randomisation; details of any restriction (such as blocking and block size)	7
Allocation concealment mechanism	9	Mechanism used to implement the random allocation sequence (such as sequentially numbered containers), describing any steps taken to conceal the sequence until interventions were assigned	7
Implementation	10	Who generated the random allocation sequence, who enrolled participants, and who assigned participants to interventions	7
Blinding	11a	If done, who was blinded after assignment to interventions (for example, participants, care providers, those	8,9

For peer review only - http://bmjopen.bmj.com/site/about/guidelines.xhtml

		assessing outcomes) and how	
	11b	If relevant, description of the similarity of interventions	N/A
Statistical methods	12a	Statistical methods used to compare groups for primary and secondary outcomes	9,10
	12b	Methods for additional analyses, such as subgroup analyses and adjusted analyses	9
**Results**			
Participant flow (a diagram is strongly recommended)	13a	For each group, the numbers of participants who were randomly assigned, received intended treatment, and were analysed for the primary outcome	Figure 1
	13b	For each group, losses and exclusions after randomisation, together with reasons	Figure 1
Recruitment	14a	Dates defining the periods of recruitment and follow-up	2,7,8
	14b	Why the trial ended or was stopped	8
Baseline data	15	A table showing baseline demographic and clinical characteristics for each group	Figure 1, table 1
Numbers analysed	16	For each group, number of participants (denominator) included in each analysis and whether the analysis was by original assigned groups	Table 2, Figures 2-5
Outcomes and estimation	17a	For each primary and secondary outcome, results for each group, and the estimated effect size and its precision (such as 95% confidence interval)	Table 2, figures 2-6
	17b	For binary outcomes, presentation of both absolute and relative effect sizes is recommended	N/A
Ancillary analyses	18	Results of any other analyses performed, including subgroup analyses and adjusted analyses, distinguishing pre-specified from exploratory	12
Harms	19	All important harms or unintended effects in each group (for specific guidance see CONSORT for harms)	12
**Discussion**			
Limitations	20	Trial limitations, addressing sources of potential bias, imprecision, and, if relevant, multiplicity of analyses	17,18
Generalisability	21	Generalisability (external validity, applicability) of the trial findings	18
Interpretation	22	Interpretation consistent with results, balancing benefits and harms, and considering other relevant evidence	19
**Other information**			
Registration	23	Registration number and name of trial registry	1,3
Protocol	24	Where the full trial protocol can be accessed, if available	3
Funding	25	Sources of funding and other support (such as supply of drugs), role of funders	21

*We strongly recommend reading this statement in conjunction with the CONSORT 2010 Explanation and Elaboration for important clarifications on all the items. If relevant, we also recommend reading CONSORT extensions for cluster randomised trials, non-inferiority and equivalence trials, non-pharmacological treatments, herbal interventions, and pragmatic trials. Additional extensions are forthcoming: for those and for up to date references relevant to this checklist, see www.consort-statement.org.

For peer review only - http://bmjopen.bmj.com/site/about/guidelines.xhtml

BMJ Open

Randomized trial of coconut oil, olive oil or butter on blood lipids and other cardiovascular risk factors in healthy men and women

Journal:	*BMJ Open*
Manuscript ID	bmjopen-2017-020167.R2
Article Type:	Research
Date Submitted by the Author:	08-Jan-2018
Complete List of Authors:	Khaw, Kay-Tee; University of Cambridge, Department of Public Health and Primary Care<br>Sharp, Stephen; University of Cambridge, MRC Epidemiology Uniot<br>Finikarides, Leila; British Broadcasting Corporation<br>Afzal, Islam; Aston University, Aston Medical Research Institute<br>Lentjes, Marleen; University of Cambridge, Department of Public Health and Primary Care<br>Luben, Robert; University of Cambridge, Department of Public Health and Primary Care<br>Forouhi, Nita; University of Cambridge, MRC Epidemiology Unit
**Primary Subject Heading**:	Nutrition and metabolism
Secondary Subject Heading:	Cardiovascular medicine, Epidemiology
Keywords:	blood lipids, dietary fats, randomized trial, coconut oil, olive oil

SCHOLARONE™ Manuscripts

5 January 2017 V4

Randomized trial of coconut oil, olive oil or butter on blood lipids and other cardiovascular risk factors in healthy men and women

Kay-Tee Khaw[1], Stephen J. Sharp[2], Leila Finikarides[3], Islam Afzal[4], Marleen Lentjes[1], Robert Luben[1], and Nita G. Forouhi[2]

Authors' Affiliations:

Kay-Tee Khaw, MBBChir, FRCP, FMedSci, Professor

Marleen Lentjes, PhD, Research Nutritionist

Robert Luben, BSc, Bioinformatics

[1]Department of Public Health and Primary Care, University of Cambridge School of Clinical Medicine, United Kingdom;

Stephen J. Sharp MA, MSc, Senior Statistician

Nita G. Forouhi, MBBS, PhD, MRCP, FFPH, Professor

[2]Medical Research Council Epidemiology Unit, University of Cambridge School of Clinical Medicine, United Kingdom;

Leila Finikarides, MSc, Producer,

[3]BBC Television"Trust Me I'm a Doctor", BBC Glasgow, Glasgow, United Kingdom; now Winton Centre for Evidence Communication, University of Cambridge, United Kingdom

Islam Afzal, PhD, Research Facilitator

[4] Aston Medical Research Institute, Aston Medical School, Aston University, Birmingham, United Kingdom

Clinical trials registration: NCT03105947 Clinical Trials.gov USNIH

Short running title: **Coconut oil, butter or olive oil and blood lipids**

Word count: text (excluding abstract, tables & references) = 5438 abstract = 586

Number of tables and figures: 3 Tables, 5 Figures, 1 supplemental table

Key words: coconut oil, butter, olive oil, dietary fat, lipids, LDL-Cholesterol, randomized trial

Correspondence to:

Kay-Tee Khaw, Professor

Clinical Gerontology Unit Box 251, University of Cambridge School of Clinical Medicine, Addenbrooke's Hospital, Cambridge, CB3 0EQ, United Kingdom

e-mail: kk101@medschl.cam.ac.uk Phone: +44 1223 336927

For peer review only - http://bmjopen.bmj.com/site/about/guidelines.xhtml

Abstract

Introduction: High dietary saturated fat intake is associated with higher blood concentrations of LDL-cholesterol, an established risk factor for coronary heart disease. However, there is increasing interest in whether various dietary oils or fats with different fatty acid profiles such as extra virgin coconut oil may have different metabolic effects but trials have reported inconsistent results. We aimed to compare changes in blood lipid profile, weight, fat distribution, and metabolic markers after four weeks consumption of 50g daily of one of three different dietary fats: extra virgin coconut oil, butter, or extra virgin olive oil: in healthy men and women in the general population.

Design: Randomized clinical trial conducted over June and July 2017.

Setting: General community in Cambridgeshire, United Kingdom

Participants: Volunteer adults were recruited by the British Broadcasting Corporation (BBC) through their websites. Eligibility criteria were men and women aged 50-75 years, with no known history of cancer, cardiovascular disease or diabetes, not on lipid lowering medication, no contraindications to a high fat diet and willingness to be randomized to consume one of the three dietary fats for four weeks. Of 160 individuals initially expressing an interest and assessed for eligibility, 96 were randomized to one of three interventions; 2 individuals subsequently withdrew and 94 men and women attended a baseline assessment. Their mean age was 60 years, 67% were women, and 98% were European Caucasian. Of these, 91 men and women attended a follow up assessment four weeks later.

Intervention: Participants were randomized to extra virgin coconut oil, extra virgin olive oil, or unsalted butter and asked to consume 50g daily of one of these fats for four weeks, which they could incorporate into their usual diet or consume as a supplement.

Main Outcomes and Measures: The primary outcome was change in serum Low Density Lipoprotein cholesterol(LDL-C); secondary outcomes were change in total and high density lipoprotein cholesterol(TC and HDL-C), TC/HDL-C ratio, and non-HDL-C; change in weight, body mass index(BMI), waist circumference, percent body fat, systolic and diastolic blood pressure, fasting plasma glucose and C-Reactive Protein.

Results: LDL-C concentrations were significantly increased on butter compared to coconut oil (+0.42, 95% CI 0.19,0.65 mmol/L, P<0.0001), and to olive oil (+0.38, 95% CI 0.16,0.60 mmol/L, P<0.0001), with no differences in change of LDL-C in coconut oil compared to olive oil (-0.04, 95% CI -0.27, 0.19 mmol/L, P=0.74). Coconut oil significantly increased HDL-C compared to

For peer review only - http://bmjopen.bmj.com/site/about/guidelines.xhtml

butter (+0.18, 95% CI 0.06,0.30 mmol/L) or olive oil (+0.16, 95% CI 0.03,0.28 mmol/L). Butter significantly increased TC/HDL-C ratio and non-HDL-C compared to coconut oil but coconut oil did not significantly differ from olive oil for TC/HDL-C and non-HDL-C. There were no significant differences in changes in weight, BMI, central adiposity, fasting blood glucose, systolic or diastolic blood pressure amongst any of the three intervention groups.

Conclusions and Relevance: Two different dietary fats (butter and coconut oil) which are predominantly saturated fats, appear to have different effects on blood lipids compared to olive oil, a predominantly monounsaturated fat with coconut oil more comparable to olive oil with respect to LDL-C. The effects of different dietary fats on lipid profiles, metabolic markers and health outcomes may vary not just according to the general classification of their main component fatty acids as saturated or unsaturated but possibly according to different profiles in individual fatty acids, processing methods, as well as the foods in which they are consumed or dietary patterns. These findings do not alter current dietary recommendations to reduce saturated fat intake in general but highlight the need for further elucidation of the more nuanced relationships between different dietary fats and health.

Clinical trials registration: NCT03105947 Clinical Trials.gov USNIH

For peer review only

Strength and limitations of the study

Strengths

- The randomized trial design comparing three dietary fat interventions minimised confounding and bias
- There was good compliance and participants were from the general community in a "real life" setting
- Objective measures of outcome- blood biochemistry and anthropometry – were used minimising bias

Limitations

- Participants were not blinded as to the intervention and the intervention was relatively short term over four weeks

For peer review only

For peer review only - http://bmjopen.bmj.com/site/about/guidelines.xhtml

Introduction

This trial was conducted in the context of debate over longstanding dietary recommendations to reduce dietary fat intake for health. The Women's Health Initiative reported no differences in cardiovascular disease in women randomized to low fat and usual diets over 8 years[1] while an intervention comparing a low fat diet with a Mediterranean diet with extra virgin olive oil, or nuts (PREDIMED) reported approximately 30% lower cardiovascular events in both Mediterranean diet arms after 4.8 years[2]; meta-analyses of observational studies and trials report inconsistent findings in the relationship between dietary saturated fatty acids and cardiovascular disease[3, 4]; and the relationships of dairy fats including milk and butter with cardiovascular disease also being debated[5-7]. Part of the debate relates to the increasing evidence that different individual fatty acids, such as the odd chain or even chain saturated fatty acids, or short, medium and long chain saturated fatty acids, may have different metabolic pathways and subsequent potential health effects, as well as the understanding that diet is more complex than individual nutrients or generic biochemical nutrient groups, and that contextual factors such as foods and dietary patterns are important. The 2015-2020 US dietary guidelines[8] now focus on foods and dietary patterns and while they recommend limiting saturated and trans fats, they no longer explicitly recommend limiting total fat. In this context therefore, there is renewed interest in the health effects of different fats and oils.

Extra virgin coconut oil has recently been promoted as a healthy oil. Though high in saturated fat, the main saturated fatty acid, lauric acid(c12:0), has been suggested to have different metabolic, and hence health effects compared to other saturated fatty acids such as palmitic acid(c16:0), predominant in butter, palm oil and animal fat. In particular, it has been suggested that coconut oil does not raise total cholesterol or LDL-Cholesterol as much as butter. A recent review on coconut oil and cardiovascular risk factors in humans concluded that the evidence of an association between coconut oil consumption and blood lipids or cardiovascular risk was mostly poor quality[9]. While some small studies have been reported comparing coconut oil and butter, these have been small[10, 11], and none conducted in the UK where overall dietary patterns are different from Asia, US or New Zealand where most trials have been conducted. The 2017 American Heart Association Presidential advisory on dietary fats and cardiovascular disease highlighted the paucity of evidence over the long term health effects of saturated fats such as coconut oil and reinforced strongly recommendations to

For peer review only - http://bmjopen.bmj.com/site/about/guidelines.xhtml

For peer review only

lower dietary saturated fat and replacement with unsaturated fat to lower LDL-cholesterol and prevent cardiovascular disease[12]. In particular, they stated “because coconut oil increases LDL-Cholesterol, a cause of cardiovascular disease, and has no known offsetting favourable effects, we advise against the use of coconut oil”[12].

Though the PREDIMED study reported lower cardiovascular disease events in those randomized to extra virgin olive oil or added nuts[2], this trial reported no overall effects on LDL-cholesterol or total cholesterol for those on olive oil compared to the low fat diet [13], results consistent with a review of intervention trials of high phenolic olive oil[14].

We therefore aimed to examine whether in free living healthy men and women in the UK, we could observe differences in blood lipids after one month’s consumption of 50g daily of one of three different fats within the context of their usual diet. Although this was a short term trial that did not address cardiovascular disease events, blood lipids are a well established risk factor for coronary heart disease and the aim was to compare directly the effects of three different fats: extra virgin coconut oil, butter (both predominantly saturated fats) with extra virgin olive oil (monounsaturated fat) on blood lipid profiles and metabolic measures, in a pragmatic trial using amounts feasible in daily diets.

For peer review only - http://bmjopen.bmj.com/site/about/guidelines.xhtml

Methods

Study population

Participants were volunteers living in the general community predominantly in the Cambridgeshire area, recruited through BBC advertising in May and June 2017. Eligible participants were men or women aged between 50-75 years who did not have a known medical history of heart disease, stroke, cancer, or diabetes, and who were not taking medication for lowering blood lipids such as statins. They had to be willing to be randomized to consume 50 g daily of one of the designated fats for four weeks, and not have any contraindications to eating a high fat diet such as gall bladder or bowel problems. Of 160 individuals expressing an interest, 96 were eligible and randomized to the intervention, 2 withdrew prior to the start of the study, and 94 attended a baseline assessment.

Allocation to Intervention

Participants were assigned a unique study identification number(ID). These ID numbers were randomized by computer generated allocation conducted by an independent statistician separately in men and women, into one of three parallel intervention arms approximately equal in size: extra virgin coconut oil, butter, or extra virgin olive oil.

Intervention

Participants attending the baseline assessment, at the end of their appointment, received one month's supply of one of the three different dietary fats to which they had been randomly allocated: extra virgin coconut oil, or butter or extra virgin olive oil. The BBC study organizer was given an ID list with the random allocation to the fats/oils and was responsible for giving each participant their supply of fat/oils. They were asked to eat 50g of these fats daily for four weeks and given measuring cups for the 50ml fat and oils: butter was prepacked in 20g and 30g portions. They were asked to continue with their usual diet, and either incorporate the fat or oil into their daily diet to substitute for other fats or oils, or they could eat these fats as a supplement. They also had information sheets with suggestions for how the fats could be consumed including recipes. The fats selected were standard products available from supermarkets bought from suppliers; organic extra virgin coconut oil, organic unfiltered extra virgin olive oil, and organic unsalted butter. Samples of the oils/fats used in the trial were sent to a reference laboratory: the West Yorkshire Analytic Services, a UKAS accredited testing service for food composition.

For peer review only - http://bmjopen.bmj.com/site/about/guidelines.xhtml

Assessments

Participants attended two assessments at a community centre in Cambridge: one at baseline before the start of the intervention in June 2017, and one at the end of four weeks in July 2017. Prior to their initial assessment, they were asked to fill in a short questionnaire about their health and lifestyle including physical activity and diet as well as complete an online 24 hour dietary assessment questionnaire with automated nutrient intake estimation, developed in Oxford, the DietWebQ[15]. All assessments were conducted between 0800 and 1230. Participants were all fasted for a minimum of 4 hours prior to attending the assessment; the majority were fasted overnight. They had height and waist circumference measured to a standardised protocol in light clothing without shoes and blood pressure measured using an automated OMRON device after being seated resting for 5 minutes. The mean of two readings for blood pressure, height and waist were used for analysis. Weight and percent body fat were measured using a Tanita body composition monitor. All measurements were conducted by two trained observers unaware of allocation to the oils/fats. Participants gave a 20 ml blood sample which was stored in a 4°C refrigerator then sent to the laboratory by courier for same day sample processing and storage for later analysis.

After four weeks at the end of the intervention, they attended again for a follow up assessment where the same measurements of height, waist circumference, blood pressure, weight and percent body fat were conducted, and another fasting 20 ml blood sample taken. Measurements were recorded on new forms and observers and participants did not have access to the measurements taken at the baseline visit. Just prior to this visit, participants were asked to fill in again the online 24 hour DietWebQ. Participants also filled in short questionnaire about their experiences on the intervention fats. This included a question about their overall experience of consuming the assigned oil/fat in the study where they were asked on average, over the past 4 weeks whether they felt mostly the same as usual, mostly felt better than usual or mostly felt worse than usual with an open ended section for comments including side effects, and overall compliance with consuming the fats which they were asked to self-rate between 0% to 100%. They were also asked whether they changed their type, level or frequency of physical activity in the past month since being in the study and had three options, no overall change in activity, increase in activity or decrease in activity.

For peer review only - http://bmjopen.bmj.com/site/about/guidelines.xhtml

Blood samples were identified only by a study ID number and were processed using standard protocols and assayed in two batches at the end of the baseline and follow up assessments in the Core Biochemical Assay Laboratory (CBAL) Cambridge University Hospitals which has UKAS Clinical Pathology Accreditation; blood samples from individuals on different interventions were thus all assayed in the same batch. The laboratory assays were conducted in a blinded fashion without any indication of the allocated intervention. Cholesterol(TC) and triglycerides were measured using enzymatic assays,[16, 17] high-density-lipoprotein cholesterol (HDL-C) was measured using a homogenous accelerator selective detergent assay automated on the Siemens Dimension RxL analyser, and low density lipoprotein cholesterol(LDL-C) was calculated from the triglyceride, HDL and cholesterol concentrations as described in the Friedewald formula (LDL = Cholesterol - HDL - (Triglycerides/2.2)[18]. Total to HDL-C ratio was computed, and non-HDL-C was computed as TC minus HDL-C.

Plasma glucose was measured using the hexokinase-glucose-6-phosphate dehydrogenase method and high sensitivity human C-Reactive Protein was assayed using an automated colourimetric immunoassay: Siemens Dimension® CCRP *Cardio*Phase® high sensitivity CRP.

Trial outcomes

The trial was registered in April 2017 with clinical trials registration: NCT03105947**.** The primary outcome of the trial was change in low density lipoprotein cholesterol (LDL-C) from baseline to follow up. Secondary outcomes were change in each of the following variables from baseline to follow up: total cholesterol (TC), high density lipoprotein cholesterol (HDL-C), triglycerides; ratio of total cholesterol/HDL-C, non-HDL cholesterol, fasting blood glucose, C-Reactive Protein, weight, body mass index(BMI), body fat %, waist circumference, systolic blood pressure and diastolic blood pressure..

Statistical analysis

The study aimed to recruit a total of 90 participants: 30 individuals per group would provide approximately 80% power to detect a difference in mean within-person change in LDL cholesterol (baseline to follow-up) comparing pairs of randomized groups (butter vs coconut oil and butter vs olive oil) of approximately 0.5 mmol/L, assuming a standard deviation of LDL cholesterol of 1.04 mmol/L[19] and a correlation between baseline and follow-up values of 0.79[20] incorporated using the method described by Borm et al[21]. With 2 primary pairwise comparisons, the significance level for each comparison was set to 2.5%.

For peer review only - http://bmjopen.bmj.com/site/about/guidelines.xhtml

This magnitude of difference was what can be estimated from metabolic ward studies in which replacement of 10% dietary calories from saturated fat is associated with 0.52 mmol/L cholesterol difference[22] though this did not specify the food sources of saturated fats, and a small intervention trial (n=28) comparing butter and coconut oil with sunflower oil[10].

Baseline characteristics were summarised separately for each randomized group. As recommended by CONSORT, no p-values were calculated for this table. The primary analysis used an Intention To Treat(ITT) population, which included all individuals in the group to which they were randomized, regardless of the extent to which they adhered to the intervention. A secondary analysis used a Per Protocol(PP) population. This was a subset of the ITT population consisting of those individuals who adhered to the intervention. Participants who reported >75% adherence when asked at the follow up visit were included in the PP population.

For each outcome, a p-value was calculated to compare the 3 randomized groups using a linear regression model, in which change from baseline was the outcome, and including a dummy variable for randomized group and the baseline value of the outcome variable as covariates, i.e. an Analysis of Covariance (ANCOVA) model. Differences between each pair of randomized groups and 95% confidence intervals (CIs) were also estimated from a similar model.

Patient and public involvement

The BBC originally proposed the idea of a study to examine claims about the health benefits of coconut oil in response to public interest; the study would be part of their "Trust me, I'm a doctor" series The study was designed as a randomized trial with participants from the general community in discussion with the BBC.

Ethics

Ethics approval was given for the study by the University of Cambridge Human Biology Research Ethics committee HBREC 2017.05.

For peer review only - http://bmjopen.bmj.com/site/about/guidelines.xhtml

Results

Figure 1 is the CONSORT diagram for the trial. The recruitment was conducted by the BBC coordinator through BBC website advertising. From 160 individuals initially expressing an interest, and after exclusion criteria, 96 individuals were randomized and invited to a baseline assessment session in June 2017. Two individuals subsequently withdrew and 94 individuals attended the baseline assessment session in June 2017. At the four week follow up assessment in July 2017, 91 individuals attended; 3 individuals did not attend follow up indicating personal circumstances.

Table 1 shows descriptive characteristics for the participants at the baseline assessment according to the allocation to dietary oils/fats. Two thirds of the participants were women and the mean age overall was 60 years.

Table 2 shows mean changes in the primary and secondary outcomes at the four week follow up within each randomized group, and comparisons between each pair of randomized groups. LDL-C concentrations were significantly increased on butter compared to coconut oil (+0.42, 95% CI 0.19,0.65 mmol/L, P<0.0001), and olive oil (+0.38L, 95% CI 0.16,0.60 mmol/L, P<0.0001), with no differences in change of LDL-C in coconut oil compared with olive oil (-0.04, 95% CI -0.27, 0.19 mmol/L, P=0.74). Coconut oil significantly increased HDL-C compared to butter (+0.18, 95% CI 0.06,0.30 mmol/L) or olive oil (+0.16, 95% CI 0.03,0.28 mmol/L).

Butter significantly increased the cholesterol/HDL-C ratio compared to coconut oil (+0.36, 95%CI 0.18,0.54) and olive oil (+0.22,95% CI 0.04,0.40) and also increased non-HDL-C compared to coconut oil (+0.39, 95% CI 0.16,0.62 mmol/L) and olive oil (+0.39(95% CI 0.16,0.62) but coconut oil did not significantly differ from olive oil for change in cholesterol/HDL-C ratio (-0.14, 95%CI -0.33,0.05) or non-HDL-C (0.002, 95% CI -0.23,0.24 mmol/L).

Coconut oil also significantly lowered C-Reactive Protein in comparison with olive oil (-0.58, 95% CI -1.12,-0.04 mg/L) but not compared to butter. There were no significant differences in changes in weight, BMI, central adiposity, fasting blood glucose, systolic or diastolic blood pressure amongst any of the three intervention groups. For weight, for example, the estimated mean(SD) changes in weight were +0.27(0.77)kg, 0.04(1.00)kg and -0.04(0.84) kg

For peer review only

For peer review only - http://bmjopen.bmj.com/site/about/guidelines.xhtml

for coconut oil, butter and olive oil respectively. Adjusting for age, sex and body mass index did not materially alter the results (supplemental table 1).

Figure 2 shows the difference in the primary outcome (LDL-C) between each pair of randomized groups in the 91 individuals who attended baseline and follow up. **Figures 3, 4, and 5** show the differences in secondary outcomes comparing butter versus coconut oil, coconut oil versus olive oil, and butter versus olive oil respectively. For comparability the differences are reported in units of baseline standard deviation (SD) for the different outcomes in Figures 3 to 5.

Self reported compliance was high: 87% of participants reported more than 75% compliance with the intervention over the 4 weeks which was similar among the groups (86% coconut oil, 88% butter and 85% olive oil). Secondary analyses on the 82 participants reporting more than 75% compliance showed similar results (not shown). Reported experience consuming the fats was similar between groups: 57%, 66%, and 60% reported feeling no different, 18%, 6% and 13% reported feeling better, and 25%, 27% and 23% reported feeling worse in the coconut oil, butter and olive oil groups respectively. Comparison of dietary intake using the 24 hour DietWebQ showed similar levels of dietary intake across intervention groups at baseline. Following the intervention, total fat intake increased in all intervention groups but estimates for absolute intakes of carbohydrate, protein and alcohol did not differ between intervention groups (Table 3). Most of the participants reported no changes in usual physical activity (79%, 73% and 89% no change; 14%, 15% and 4% increased usual physical activity and 7%, 12% and 7% decreased usual physical activity in the coconut oil, butter and olive oil groups respectively). In a post hoc exploratory analysis, exclusion of individuals who reported increasing usual physical activity had little effect on significant differences between interventions for LDL-C and HDL-C and did not alter the findings for weight change (supplemental table 2).

Supplemental appendix 1 shows the fatty acid composition of the three oils/fats used in the intervention. Coconut oil was 94 % saturated fatty acids, of which the main components were lauric acid C12:0(48%), myristic acid C14:0(19%), and palmitic acid C16:0(9%). Butter was 66% saturated fatty acids, of which the main components were palmitic acid C16:0(28%), stearic

For peer review only - http://bmjopen.bmj.com/site/about/guidelines.xhtml

acid C18:0(12%), and myristic acid C14:0(11%). Olive oil was 19% saturated fatty acids, mainly palmitic acid C16 (15%) with stearic acid C18:0 (3%) and 68% monounsaturates with the main component being oleic acid C18:1n9(64%). These profiles are very similar to those reported from other studies[9].

For peer review only

For peer review only - http://bmjopen.bmj.com/site/about/guidelines.xhtml

Discussion

In this trial, middle aged men and women living in the general community were randomly allocated to consume 50g extra virgin coconut oil, or 50g butter, or 50g extra virgin olive oil for four weeks. We observed at the end of the trial significantly different changes in LDL-C and HDL-C concentrations between the three intervention groups; in pairwise comparisons, coconut oil did not significantly raise LDL-C concentrations compared to olive oil while butter significantly raised LDL-C concentrations compared to both coconut oil and olive oil. Coconut oil significantly raised HDL-C concentrations compared to both butter and olive oil. Butter also significantly raised cholesterol/HDL-C ratio and non-HDL-Cholesterol more than both coconut oil and olive oil but there were no differences between coconut oil and olive oil for changes in cholesterol/HDL-C and non-HDL-C cholesterol.

There were no significant differences in weight or BMI change, change in central adiposity as measured by waist circumference or percent body fat. There were also no significant differences in change in fasting glucose, or systolic and diastolic blood pressure among the three different fat interventions. In pairwise comparison, coconut oil significantly lowered C-Reactive Protein compared to olive oil but there were no significant differences between coconut oil and butter for C-Reactive Protein.

The results were somewhat surprising for a number of reasons. Coconut oil is predominantly (approximately 90%) saturated fat which is generally held to have an adverse effect on blood lipids by increasing blood LDL-C concentrations. However, the saturated fatty acid profiles of different dietary fats vary substantially; coconut oil is predominantly (around 48%) lauric acid (12:0) compared to butter (66% saturated fat) which is about 40% palmitic (16:0) and stearic (18:0) acids, leading to suggestions that coconut oil may not have the same health effects as other foods high in saturated fat[9]. Nevertheless, though reviews on coconut oil and cardiovascular disease risk factors have concluded that the evidence of an association between coconut oil consumption and blood lipids or cardiovascular risk was mostly poor quality[9], trials have generally reported that coconut oil consumption raises LDL-C in comparison to polyunsaturated oil such as safflower oil, though not as much in comparison to butter[10, 11].

For peer review only - http://bmjopen.bmj.com/site/about/guidelines.xhtml

Based on 3 randomized crossover trials of good scientific quality, one trial reported butter increased LDL-C more than coconut oil which raised LDL-C more compared to safflower oil[10]; a second that coconut oil raised LDL-C more than beef fat which raised LDL-C more than safflower oil[23], and a third reported that coconut oil raised LDL-C more than palm oil which raised LDL-C more than olive oil[24]. The current study observed that butter raised LDL-C more than coconut oil but that coconut oil did not differ from olive oil. Two studies showed higher HDL-C with coconut oil compared with other fats whether beef fat, safflower oil or olive oil[23, 24]. Thus far, the current results are consistent with previous studies indicating that butter raises LDL-C more than coconut oil, and also that coconut oil also raises HDL-C. However, the present study is an exception in not finding any increase in LDL-C compared to an unsaturated oil, in this case, olive oil. In this trial the difference of 0.33mmol/L in LDL-C on butter compared to olive oil is consistent with previous studies[25].

This is the largest trial reported to date on coconut oil and lipids apart from a recent study of 200 individuals with established coronary heart disease comparing coconut oil with sunflower oil over 2 years that reported no differences in blood lipids but virtually all the participants were on statin therapy[26] which makes findings difficult to interpret.

Direct comparisons between studies are problematic because of different oils used; we used extra virgin olive oil as a comparison group rather than a polyunsaturated oil such as safflower or sunflower oil, for feasibility reasons of likely participant compliance with the requirement for 50g intake daily. The PREDIMED study reported no significant difference in change in LDL–C or total cholesterol but significant lowering of the cholesterol/HDL-C ratio in the Mediterranean diet supplemented with extra virgin olive oil compared to a low fat diet[2, 13]. A recent review reported that high phenolic olive oil does not modify the lipid profile compared to its low phenolic counterpart[14] though other studies have reported that extra virgin olive oil decreases LDL-C directly measured as concentrations of apoB-100 and the total number of LDL particles as assessed by NMR spectroscopy[27, 28]. We therefore expected coconut oil would raise LDL-C compared to olive oil, but in the current study we observed no evidence of an overall average increase in LDL-C in individuals allocated either to the coconut oil or olive oil intervention.

For peer review only - http://bmjopen.bmj.com/site/about/guidelines.xhtml

For peer review only

Lack of compliance with consuming the dietary fat would lead to no differences between groups and hence explain the lack of differences in LDL-C between coconut oil and olive oil groups. However, in this group of volunteers, reported compliance was high and did not differ between groups; in addition, those in the coconut oil group had significantly greater increases in HDL-C compared to those allocated to olive oil or butter so lack of compliance is unlikely to be an explanation.

The predominant fatty acid in coconut oil, lauric acid(C12:0) as well as myristic acid(C14:0) are medium chain fatty acids that are rapidly absorbed, taken up by the liver and oxidized to increase energy expenditure which is a possible explanation for why coconut oil may have different effects compared to other saturated fats[29]. It is also possible that differences could be attributed to the use of extra virgin preparations of coconut oil rather than standard coconut oil; different methods of preparation such as the chilling method for virgin coconut oil compared to refined, bleached and deodorized coconut oil may influence phenolic compounds and antioxidant activity[30] thus, processing of oils changes their composition, biological properties and consequent potential metabolic effects. The variations in possible health effects resulting from variations in processing of different fats is well documented in the large literature on hydrogenation of polyunsaturated oils to make solid margarines which may increase harmful trans- fats[31]. In this context it is notable that the major trial (PREDIMED) reporting reduction in cardiovascular risk with a Mediterranean diet used extra virgin olive oil[2], while other studies which reported null findings with olive oil may not have always specified the product used[14].

There was no evidence of difference between groups in mean weight, BMI, percent body fat, or central adiposity at the end of this trial; however, these were secondary endpoints for which the trial was not specifically powered. Nevertheless the estimated 95% CI around mean weight differences at the end for the trial were not large. The participants were asked to consume 50g of fat or oils daily. They could do this in the context of their usual diet by substituting for their usual fats, or by consuming these as a supplement. In practice, most participants reported finding it difficult to substitute the different fats or oils for cooking in their usual diet and usually consumed these as a supplement. These fats if taken in addition

For peer review only - http://bmjopen.bmj.com/site/about/guidelines.xhtml

to their usual diet would have been approximately 450 additional calories daily, which if consistently taken four weeks might be expected to be nearly 13,000 additional calories resulting in likely weight gain of 1 to 2kg. This information was provided in the information sheet with the informed consent for participants. While it is possible that participants may have consciously changed behaviours to maintain body weight such as reducing their other dietary intake because of the additional fat or being more physically active, many participants reported that the high fat diet resulted in feeling full and eating less.

It is also possible that even though this was a randomized trial, in an unblinded study, participants may have changed behaviours differentially in the different intervention groups resulting in differences in lipids or lack of differences in weight observed rather than being attributed to the dietary fat interventions. The majority of the participants reported no change in usual physical activity though slightly more participants in the coconut oil and butter groups reported increasing usual physical activity (14% and 15% respectively) compared to 4% in the olive oil group. Nevertheless exclusion of all individuals reporting increased usual physical activity from the analyses did not change the findings. Dietary factors apart from fat most likely to influence HDL-C, total alcohol intake or change in alcohol intake, did not differ significantly between intervention groups and in fact alcohol intake decreased slightly during the trial which would not explain any increases in HDL-C observed. There is therefore no evidence to suggest that differences in lipids, or lack of differences in weight change were likely to be attributed to differential changes in behaviour.

The main strengths of this study are the randomized design with high completion rate (91/94 individuals returned to follow up) and self-reported dietary compliance (nearly 90% participants with over 75% adherence) over four weeks. This is also larger than most trials reported with the exception of the trial in India in individuals with heart disease most of whom were taking statins[26]. The current trial by contrast, was conducted in individuals in the general population.

This trial has limitations. It was a short term trial of four weeks intervention so we are unable to know what would have happened if the intervention had continued for a longer period.

For peer review only - http://bmjopen.bmj.com/site/about/guidelines.xhtml

Moreover, the current findings only apply to the intermediate metabolic (lipid) risk markers and cannot be extended to findings for clinical endpoints.

It was designed as a pragmatic trial in free living individuals rather than a controlled metabolic ward trial such that individuals were asked only to consume the 50g of allocated fat or oil daily. As this was a "real-world" study, we made no attempt to control other aspects of their usual diet in particular, total energy intake. For this reason, our results cannot be taken to reflect what would happen when the only change to a diet is the substitution of one fat with another (e.g. replacing butter with coconut oil; or replacing butter with olive oil). Individuals may have changed their behaviours in different ways to accommodate this additional fat, whether by modifying other aspects of their diet for instance, increasing foods such as bread and potatoes or salads to eat with the fats, or consciously reducing other food intake or changing physical activity patterns to control energy balance. Nevertheless, this trial is more reflective of real life situations.

While self-reported compliance was high, this was subjective and we did not measure the blood fatty acid profile in participants following the intervention for an objective biomarker of compliance. Nevertheless, we did observe differential changes in blood lipids during the intervention.

The generalisability of the findings to the wider population is also unclear. The volunteers were clearly highly selected to be willing to participate in such a study, and also likely to be healthier than the general population, as for ethical reasons we excluded those with known prevalent cardiovascular disease, cancer or diabetes and also those on any lipid lowering medication or other contraindications to a high fat diet. Nevertheless, it is unlikely that the effect of these dietary fats in this group of individuals recruited from the general population would be biologically different from the general population.

Implications

We focussed on LDL-Cholesterol for the primary endpoint as the causal relationship between LDL-C concentrations and coronary heart disease risk is well established, with about a 15% increase in coronary heart disease risk per 1 mmol/L increase in LDL-C concentrations, and

For peer review only - http://bmjopen.bmj.com/site/about/guidelines.xhtml

reduction of LDL-C cholesterol lowers coronary heart disease risk[32]. Increase in LDL-C concentrations has been the main mechanism through which dietary saturated fat is believed to increase heart disease risk, though other pathways have been postulated. However, it is notable that some Mediterranean diet interventions such as the Lyon heart stud (alpha linolenic acid)[33] or PREDIMED (extra virgin olive oil) [2] which have been reported to reduce cardiovascular risk in secondary and primary prevention may have effects through other pathways such as inflammation or endothelial function[34, 35]. Whatever the mechanisms, the evidence from prospective studies is consistent and strong that substitution of saturated fats by unsaturated fats is beneficial for cardiovascular risk[36].

For peer review only

The results of this study indicate that two different dietary fats(coconut oil and butter)which are predominantly saturated fats, appear to have different effects on blood lipids compared to olive oil, a predominantly monounsaturated fat. The effects of different dietary fats on lipid profiles, metabolic markers and health outcomes may vary not just according to the general classification of their main component fatty acids as saturated or unsaturated but possibly according to different profiles in individual fatty acids, processing methods, as well as the foods in which they are consumed or dietary patterns. There is increasing evidence that associations of saturated fatty acids with health outcomes may vary according to whether they are odd or even chain saturated fatty acids, or their chain length[37-39]. Indeed, while overall the evidence indicates the substitution of dietary saturated fats with polyunsaturated fats is beneficial for coronary heart disease risk[40] heterogeneity in findings from observational studies and trials may reflect different dietary sources of fats[4, 41] As the Joint FAO/WHO 2008 Expert Consultation on Fats and Fatty Acids in Human Nutrition comments:
"There are inherent limitations with the convention of grouping fatty acids based only on number of double bonds….major groups of fatty acids are associated with different health effects…..individual fatty acids within each broad classification may have unique biological properties or effects…. Intakes of individual fatty acids differ across world depending on predominant food sources of total fats and oils." The associations with health endpoints may well vary depending on the food sources.

For peer review only - http://bmjopen.bmj.com/site/about/guidelines.xhtml

In this trial, extra virgin coconut oil was similar to olive oil and did not raise LDL-C in comparison with butter. The current short-term trial on an intermediate cardiovascular disease risk factor, LDL-C, does not provide evidence to modify existing prudent recommendations to reduce saturated fat in the diet as emphasized in most consensus recommendations[8, 12] and dietary guidelines should be based on a range of criteria[42]. However, the findings highlight the need for further elucidation of the more nuanced relationships between different dietary fats and health. There is increasing evidence that to understand the relationship between diet and health, we need to go beyond simplistic associations between individual nutrients and health outcomes and examine foods and dietary patterns as a whole. In particular, present day diets with high intakes of processed foods now incorporate many fats and oils such as soya bean oil, palm oil and coconut oil which have not been previously widely used in Western societies and not well studied. The relationships between different dietary fats, particularly some of the now more commonly used fats, and health endpoints such as cardiovascular disease events need to be better established.

For peer review only

For peer review only - http://bmjopen.bmj.com/site/about/guidelines.xhtml

Funding statement

This work was supported by the British Broadcasting Corporation, a National Institute of Health Research Senior Investigator Award to KTK and core MRC Epidemiology support (MC UU 12015/5).

Acknowledgements

This study was conducted in collaboration with the British Broadcasting Corporation (BBC) which provided support for the recruitment of participants, running of the community assessment clinic, and biochemistry measurements for lipids. Other costs were supported by the University of Cambridge through a National Institute of Health Research Senior Investigator Award to Kay-Tee Khaw. Nita G Forouhi acknowledge core MRC Epidemiology Support (MC UU 12015/5). We thank Keith Burling and Peter Barker from the Core Biochemical Assay Laboratory, CBAL in Cambridge for the laboratory assays, Shrikant Bangdiwala, University of North Carolina for conducting the computer generated random allocation of participants to the interventions, Timothy Key and colleagues at Oxford University for the use of the DietWebQ, and Nichola Dalzell and Shabina Hayat, Department of Public Health and Primary Care, and Eirini Trichia, Richard Powell and Meriel Smith,MRC Epidemiology Unit, University of Cambridge for logistical support. We thank the Cambridge Yoga Centre which hosted the assessment sessions for participants in June and July 2017. Most of all, we thank the participants from the general community who generously volunteered to take part in this trial; this study would not have been possible without their efforts and we are most grateful to them.

The BBC and the University of Cambridge collaborated in the design and conduct of the study, data collection and management of the study. The University of Cambridge investigators were solely responsible for the analysis and interpretation of the data, and preparation of the manuscript. The BBC producer coordinating the study (LF) is a co author who has reviewed and approved the manuscript but the BBC has otherwise had no editorial role in the manuscript.

Competing interest statement

For peer review only - http://bmjopen.bmj.com/site/about/guidelines.xhtml

All authors have completed the Unified Competing Interest form and declare no support from any organisation for the submitted work except as listed in the acknowledgements;; no financial relationships with any organisations that might have an interest in the submitted work in the previous three years and, no other relationships or activities that could appear to have influenced the submitted work

Conflicts of interest

None

Copyright

The Corresponding Author has the right to grant on behalf of all authors and does grant on behalf of all authors, a worldwide licence to the Publishers and its licensees in perpetuity, in all forms, formats and media (whether known now or created in the future), to i) publish, reproduce, distribute, display and store the Contribution, ii) translate the Contribution into other languages, create adaptations, reprints, include within collections and create summaries, extracts and/or, abstracts of the Contribution, iii) create any other derivative work(s) based on the Contribution, iv) to exploit all subsidiary rights in the Contribution, v) the inclusion of electronic links from the Contribution to third party material whereever it may be located; and, vi) license any third party to do any or all of the above

Ethics and Consent

Ethics approval was given by the University of Cambridge Human Biology Research Ethics Committee Application no. HBREC.2017.05. All participants gave signed informed consent. Clinical Trials registration April 2017 NCT03105947 USNIH Clinical Trials.gov

Contributors and transparency declaration

Kay-Tee Khaw had full access to all of the data in the study and takes responsibility for the integrity of the data and the accuracy of the data analysis. The lead author and guarantor Khaw affirms that the manuscript is an honest, accurate, and transparent account of the study being reported; that no important aspects of the study have been omitted; and that any discrepancies from the study as planned have been explained.

Study concept and design: Khaw, Forouhi, Finikarides

For peer review only - http://bmjopen.bmj.com/site/about/guidelines.xhtml

Acquisition of data: Khaw, Forouhi, Finikarides, Afzal, Luben, Lentjes

Analysis and interpretation of the data: Sharp, Khaw, Forouhi

Drafting of the manuscript: Khaw

Critical revision of the manuscript for important intellectual content: Forouhi, Sharp, Afzal, Finkarides, Luben, Lentjes

Obtaining funding: Khaw, Finikarides, Forouhi

Administrative, technical or material support: Khaw, Forouhi, Finikarides, Afzal, Luben, Sharp, Lentjes

Data sharing statement

Data are available. Please contact corresponding author.

For peer review only

Reference List

1. Howard BV, Van HL, Hsia J et al. Low-fat dietary pattern and risk of cardiovascular disease: the Women's Health Initiative Randomized Controlled Dietary Modification Trial. JAMA 2006;295(6):655-666.

2. Estruch R, Ros E, Salas-Salvado J et al. Primary prevention of cardiovascular disease with a Mediterranean diet. N Engl J Med 2013;368(14):1279-1290.

3. Chowdhury R, Warnakula S, Kunutsor S et al. Association of dietary, circulating, and supplement fatty acids with coronary risk: a systematic review and meta-analysis. Ann Intern Med 2014;160(6):398-406.

4. Hooper L, Martin N, Abdelhamid A, Davey SG. Reduction in saturated fat intake for cardiovascular disease. Cochrane Database Syst Rev 2015;(6):CD011737.

5. Alexander DD, Bylsma LC, Vargas AJ et al. Dairy consumption and CVD: a systematic review and meta-analysis. Br J Nutr 2016;115(4):737-750.

6. Liang J, Zhou Q, Kwame AW, Su Y, Zhang Z. Biomarkers of dairy fat intake and risk of cardiovascular disease: a systematic review and meta analysis of prospective studies. Crit Rev Food Sci Nutr 2016;0.

7. Pimpin L, Wu JH, Haskelberg H, Del GL, Mozaffarian D. Is Butter Back? A Systematic Review and Meta-Analysis of Butter Consumption and Risk of Cardiovascular Disease, Diabetes, and Total Mortality. PLoS One 2016;11(6):e0158118.

8. Department of Health and Human Services U, Department of Agriculture (US). 2015-2020 Dietary Guidelines for Americans. 8th Edition. 2015

9. Eyres L, Eyres MF, Chisholm A, Brown RC. Coconut oil consumption and cardiovascular risk factors in humans. Nutr Rev 2016;74(4):267-280.

10. Cox C, Mann J, Sutherland W, Chisholm A, Skeaff M. Effects of coconut oil, butter, and safflower oil on lipids and lipoproteins in persons with moderately elevated cholesterol levels. J Lipid Res 1995;36(8):1787-1795.

11. Cox C, Sutherland W, Mann J, de JS, Chisholm A, Skeaff M. Effects of dietary coconut oil, butter and safflower oil on plasma lipids, lipoproteins and lathosterol levels. Eur J Clin Nutr 1998;52(9):650-654.

12. Sacks FM, Lichtenstein AH, Wu JHY et al. Dietary Fats and Cardiovascular Disease: A Presidential Advisory From the American Heart Association. Circulation 2017;136(3):e1-e23.

13. Estruch R, Martinez-Gonzalez MA, Corella D et al. Effects of a Mediterranean-style diet on cardiovascular risk factors: a randomized trial. Ann Intern Med 2006;145(1):1-11.

14. Hohmann CD, Cramer H, Michalsen A et al. Effects of high phenolic olive oil on cardiovascular risk factors: A systematic review and meta-analysis. Phytomedicine 2015;22(6):631-640.

For peer review only - http://bmjopen.bmj.com/site/about/guidelines.xhtml

15. Liu B, Young H, Crowe FL et al. Development and evaluation of the Oxford WebQ, a low-cost, web-based method for assessment of previous 24 h dietary intakes in large-scale prospective studies. Public Health Nutr 2011;14(11):1998-2005.

16. HAGEN JH, HAGEN PB. An enzymic method for the estimation of glycerol in blood and its use to determine the effect of noradrenaline on the concentration of glycerol in blood. Can J Biochem Physiol 1962;40:1129-1139.

17. Rautela GS, Liedtke RJ. Automated enzymic measurement of total cholesterol in serum. Clin Chem 1978;24(1):108-114.

18. Nauck M, Warnick GR, Rifai N. Methods for measurement of LDL-cholesterol: a critical assessment of direct measurement by homogeneous assays versus calculation. Clin Chem 2002;48(2):236-254.

19. Canoy D, Wareham N, Luben R et al. Serum lipid concentration in relation to anthropometric indices of central and peripheral fat distribution in 20,021 British men and women: results from the EPIC-Norfolk population-based cohort study. Atherosclerosis 2006;189(2):420-427.

20. Forouhi NG, Menon RK, Sharp SJ et al. Effects of vitamin D2 or D3 supplementation on glycaemic control and cardiometabolic risk among people at risk of type 2 diabetes: results of a randomized double-blind placebo-controlled trial. Diabetes Obes Metab 2016;18(4):392-400.

21. Borm GF, Fransen J, Lemmens WA. A simple sample size formula for analysis of covariance in randomized clinical trials. J Clin Epidemiol 2007;60(12):1234-1238.

22. Clarke R, Frost C, Collins R, Appleby P, Peto R. Dietary lipids and blood cholesterol: quantitative meta-analysis of metabolic ward studies. BMJ 1997;314(7074):112-117.

23. Reiser R, Probstfield JL, Silvers A et al. Plasma lipid and lipoprotein response of humans to beef fat, coconut oil and safflower oil. Am J Clin Nutr 1985;42(2):190-197.

24. Voon PT, Ng TK, Lee VK, Nesaretnam K. Diets high in palmitic acid (16:0), lauric and myristic acids (12:0 + 14:0), or oleic acid (18:1) do not alter postprandial or fasting plasma homocysteine and inflammatory markers in healthy Malaysian adults. Am J Clin Nutr 2011;94(6):1451-1457.

25. Engel S, Tholstrup T. Butter increased total and LDL cholesterol compared with olive oil but resulted in higher HDL cholesterol compared with a habitual diet. Am J Clin Nutr 2015;102(2):309-315.

26. Vijayakumar M, Vasudevan DM, Sundaram KR et al. A randomized study of coconut oil versus sunflower oil on cardiovascular risk factors in patients with stable coronary heart disease. Indian Heart J 2016;68(4):498-506.

27. Hernaez A, Fernandez-Castillejo S, Farras M et al. Olive oil polyphenols enhance high-density lipoprotein function in humans: a randomized controlled trial. Arterioscler Thromb Vasc Biol 2014;34(9):2115-2119.

28. Hernaez A, Remaley AT, Farras M et al. Olive Oil Polyphenols Decrease LDL Concentrations and LDL Atherogenicity in Men in a Randomized Controlled Trial. J Nutr 2015;145(8):1692-1697.

29. DeLany JP, Windhauser MM, Champagne CM, Bray GA. Differential oxidation of individual dietary fatty acids in humans. Am J Clin Nutr 2000;72(4):905-911.

For peer review only - http://bmjopen.bmj.com/site/about/guidelines.xhtml

For peer review only

30. Marina AM, Man YB, Nazimah SA, Amin I. Antioxidant capacity and phenolic acids of virgin coconut oil. Int J Food Sci Nutr 2009;60 Suppl 2:114-123.

31. Kummerow FA. The negative effects of hydrogenated trans fats and what to do about them. Atherosclerosis 2009;205(2):458-465.

32. Goodman DS, Hulley SB, Clark LT. Report of the national cholesterol education program expert panel on detection, evaluation, and treatment of high blood cholesterol in adults. Archives of Internal Medicine 1988;148(1):36-69.

33. de LM, Renaud S, Mamelle N et al. Mediterranean alpha-linolenic acid-rich diet in secondary prevention of coronary heart disease. Lancet 1994;343(8911):1454-1459.

34. Casas R, Sacanella E, Urpi-Sarda M et al. The effects of the mediterranean diet on biomarkers of vascular wall inflammation and plaque vulnerability in subjects with high risk for cardiovascular disease. A randomized trial. PLoS One 2014;9(6):e100084.

35. Casas R, Sacanella E, Urpi-Sarda M et al. Long-Term Immunomodulatory Effects of a Mediterranean Diet in Adults at High Risk of Cardiovascular Disease in the PREvencion con DIeta MEDiterranea (PREDIMED) Randomized Controlled Trial. J Nutr 2016;146(9):1684-1693.

36. Li Y, Hruby A, Bernstein AM et al. Saturated Fats Compared With Unsaturated Fats and Sources of Carbohydrates in Relation to Risk of Coronary Heart Disease: A Prospective Cohort Study. J Am Coll Cardiol 2015;66(14):1538-1548.

37. Khaw KT, Friesen MD, Riboli E, Luben R, Wareham N. Plasma phospholipid fatty acid concentration and incident coronary heart disease in men and women: the EPIC-Norfolk prospective study. PLoS Med 2012;9(7):e1001255.

38. Praagman J, Beulens JW, Alssema M et al. The association between dietary saturated fatty acids and ischemic heart disease depends on the type and source of fatty acid in the European Prospective Investigation into Cancer and Nutrition-Netherlands cohort. Am J Clin Nutr 2016;103(2):356-365.

39. Forouhi NG, Koulman A, Sharp SJ et al. Differences in the prospective association between individual plasma phospholipid saturated fatty acids and incident type 2 diabetes: the EPIC-InterAct case-cohort study. Lancet Diabetes Endocrinol 2014;2(10):810-818.

40. Mozaffarian D, Micha R, Wallace S. Effects on coronary heart disease of increasing polyunsaturated fat in place of saturated fat: a systematic review and meta-analysis of randomized controlled trials. PLoS Med 2010;7(3):e1000252.

41. Ramsden CE, Zamora D, Leelarthaepin B et al. Use of dietary linoleic acid for secondary prevention of coronary heart disease and death: evaluation of recovered data from the Sydney Diet Heart Study and updated meta-analysis. BMJ 2013;346:e8707.

42. Smit LA, Mozaffarian D, Willett W. Review of fat and fatty acid requirements and criteria for developing dietary guidelines. Ann Nutr Metab 2009;55(1-3):44-55.

For peer review only

For peer review only - http://bmjopen.bmj.com/site/about/guidelines.xhtml

5 January 2017 V4

Table 1
Descriptive characteristics at baseline assessment of participants in the COB trial according to allocation (intention to treat)

	Coconut oil N=29		Butter N=33		Olive Oil N=32	
	**Mean**	**(SD)**	**Mean**	**(SD)**	**Mean**	**(SD)**
Age (years)	59.1	(6.1)	61.5	(5.8)	59.1	(6.4)
LDL-Cholesterol (mmol/l)	3.5	(0.9)	3.5	(0.9)	3.7	(1.0)
Total cholesterol (mmol/l)	5.9	(1.0)	5.9	(1.0)	6.0	(0.9)
HDL-Cholesterol (mmol/l)	2.0	(0.5)	1.9	(0.5)	1.8	(0.5)
Cholesterol/HDL ratio	3.2	(0.9)	3.2	(0.8)	3.5	(1.2)
Non HDL-Cholesterol (mmol/l)	3.9	(1.0)	4.0	(0.9)	4.2	(1.1)
Glucose (mmol/l)	5.3	(0.4)	5.4	(0.5)	5.4	(0.5)
Weight (kg)	73.9	(15.1)	70.8	(11.7)	71.1	(14.5)
Waist (cm)	85.4	(11.9)	83.7	(8.1)	86.2	(11.5)
Body fat (%)	29.7	(10.2)	29.2	(9.0)	31.5	(9.6)
Body Mass Index (kg/m2)	25.5	(4.5)	24.8	(3.5)	25.0	(4.5)
Systolic blood pressure (mmHg)	131.4	(18.8)	136.5	(18.8)	133.1	(16.5)
Diastolic blood pressure (mmHg)	79.8	(9.3)	81.0	(12.0)	78.1	(6.7)
DietWebQ intake/day						
Total energy (MJ)	9.00	(3.70)	8.23	(2.17)	9.51	(3.5)
Protein % energy	14.8	(4.4)	16.0	(3.7)	15.7	(3.0)
Carbohydrate % energy	43.6	(8.9)	41.4	(8.7)	42.7	(11.7)
Total fat% energy	37.3	(7.3)	36.7	(8.7)	36.4	(10.3)
Saturated fat% energy	14.1	(3.6)	13.3	(4.4)	13.4	(4.9)
Alcohol % energy	4.2	(5.4)	5.9	(7.5)	5.1	(6.1)
Hours of walking in past week	8.9	(9.5)	10.9	(12.3)	10.1	(8.7)
Hours of cycling in past week	1.8	(2.6)	2.0	(2.5)	2.7	(5.5)
Hours of other physical exercise in past week	3.4	(3.4)	2.3	(4.0)	1.8	(2.6)

For peer review only

For peer review only - http://bmjopen.bmj.com/site/about/guidelines.xhtml

5 January 2017 V4

Table 1 continued Descriptive characteristics at baseline assessment of participants in the COB trial according to allocation (intention to treat)

	Coconut oil N=29		Butter N=33		Olive Oil N=32	
	**Median**	**(IQR)**	**Median**	**(IQR)**	**Median**	**(IQR)**
Triglycerides (mmol/l)	0.89	(0.74,1.10)	0.92	(0.70,1.20)	0.94	(0.79,1.31)
C-Reactive Protein (mg/l)	1.04	(0.47,2.15)	1.08	(0.64,2.13)	1.13	(0.58,2.67)
	**%**	**(N)**	**%**	**(N)**	**%**	**(N)**
Sex						
Men	37.9	(11)	33.3	(11)	28.1	(9)
Women	62.1	(18)	66.7	(22)	71.9	(23)
Ethnicity						
White	96.6	(28)	97.0	(32)	93.8	(30)
Non-white	3.4	(1)	3.0	(1)	3.1	(1)
Smoking status						
Never	58.6	(17)	66.7	(22)	68.8	(22)
Former	34.5	(10)	33.3	(11)	25.0	(8)
Current	6.9	(2)	0.0	(0)	6.3	(2)
Alcohol consumption in past year						
Never or once per month	20.7	(6)	30.3	(10)	28.1	(9)
1-4 times per week	72.4	(21)	48.5	(16)	59.4	(19)
Almost every day or every day	6.9	(2)	21.2	(7)	12.5	(4)
Highest level of education						
School to age 16	13.8	(4)	12.1	(4)	15.6	(5)
School to age 18	27.6	(8)	9.1	(3)	9.4	(3)
University	58.6	(17)	78.8	(26)	75.0	(24)
Currently in paid job						
No	20.7	(6)	45.5	(15)	25.0	(8)
Yes	75.9	(22)	54.5	(18)	75.0	(24)

IQR: Interquartile range

For peer review only - http://bmjopen.bmj.com/site/about/guidelines.xhtml

5 January 2017 V4

Table 2
Mean change in variables between baseline and follow up after dietary interventions and pairwise comparisons between fats in 91 participants

	Change from Baseline				Pairwise comparisons		
	Coconut oil N=28 Mean (SD)	Butter N=33 Mean (SD)	Olive Oil N=30 Mean (SD)	P value Comparison Between groups	Coconut oil vs olive oil Difference (95% CI)	Butter vs Coconut oil Difference (95% CI)	Butter vs olive oil Difference (95% CI)
LDL-Cholesterol mmol/L	-0.09 (0.49)	0.33 (0.48)	-0.06 (0.39)	<0.001	-0.04 (-0.27, 0.19)	0.42 (0.19,0.65)	0.38 (0.16,0.60)
Total cholesterol mmol/L	0.22 (0.55)	0.42 (0.59)	0.03 (0.43)	0.022	0.19 (-0.08,0.46)	0.19(-0.08,0.45)	0.38 (0.11,0.64)
HDL-Cholesterol mmol/L	0.28 (0.29)	0.09 (0.27)	0.10 (0.15)	0.009	0.16 (0.03,0.28)	-0.18 (-0.30,-0.06)	-0.02 (-0.14,0.09)
Triglycerides mmol/L	0.07 (0.58)	-0.001 (0.36)	-0.03 (0.27)	0.65	0.10 (-0.12,0.32)	-0.08 (-0.29,0.13)	0.02 (-0.19,0.23)
Cholesterol/HDL ratio	-0.26 (0.36)	0.10 (0.41)	-0.13 (0.32)	<0.001	-0.14 (-0.33,0.05)	0.36 (0.18,0.54)	0.22 (0.04,0.40)
Non HDL-Cholesterol mmol/L	-0.06 (0.44)	0.33 (0.51)	-0.07 (0.42)	0.001	0.002 (-0.23,0.24)	0.39 (0.16,0.62)	0.39 (0.16,0.62)
Glucose mmol/L	-0.05 (0.49)	0.02 (0.48)	-0.06 (0.49)	0.68	0.01 (-0.23,0.25)	0.08(-0.15,0.32)	0.09 (-0.14,0.33)
C-Reactive Protein mg/L	-0.31 (1.09)	-0.04 (0.93)	0.23 (1.40)	0.11	-0.58 (-1.12,-0.04)	0.29 (-0.24,0.82)	-0.29 (-0.80,0.23)
Weight Kg	0.27 (0.77)	0.04 (1.00)	-0.04 (0.84)	0.42	0.30 (-0.16, 0.76)	-0.22 (-0.67, 0.23)	0.08 (-0.36, 0.52)
Waist cm	1.29 (3.31)	0.26 (3.43)	0.59 (3.25)	0.52	0.71 (-1.00,2.42)	-0.95 (-2.63,0.72)	-0.24 (-1.89, 1.41)
Body fat %	0.24 (1.03)	0.34 (1.31)	0.13 (1.30)	0.82	0.09 (-0.54,0.73)	0.10 (-0.52,0.72)	0.19 (-0.42, 0.81)
Body Mass Index kg/m2	0.09 (0.27)	0.02 (0.35)	-0.01 (0.29)	0.13	0.10 (-0.06,0.26)	-0.07 (-0.22,0.09)	0.03 (-0.12, 0.18)
Systolic blood pressure mmHg	0.18 (11.46)	-3.79 (11.11)	-3.67 (8.23)	0.29	3.91 (-1.22, 9.04)	-3.22 (-8.26, 1.82)	0.69 (-4.26,5.64)
Diastolic blood pressure mmHg	-2.02 (5.71)	-1.33 (6.24)	-0.45 (8.48)	0.81	-0.73 (-3.88, 2.42)	0.99 (-2.08,4.05)	0.26 (-2.78,3.30)

For peer review only - http://bmjopen.bmj.com/site/about/guidelines.xhtml

5 January 2017 V4

Table 3
Baseline and follow up dietary intake by allocation to coconut oil, butter or olive oil* estimated using 24 hour DietWebQ

DietWebQ intake/day	Coconut oil	Butter	Olive oil
Baseline prior to start of intervention	N=27	n=33	n=32
Energy MJ/d	9.0 (3.7)	8.2 (2.2)	9.5 (3.5)
Total fat g/d	94 (47)	81 (26)	98 (50)
Protein g/d	74 (29)	75 (19)	87 (34)
Carbohydrate g/d	238 (95)	215 (75)	243(95)
Alcohol g/d	16(22)	17 (23)	18(22)
At four weeks of intervention	n=24	n=32	n=27
Energy MJ/d	9.6 (3.2)	8.6 (2.4)	9.6 (3.1_
Total fat g/d	127 (47)	94 (37)	138 (38)
Protein g/d	71 (25)	77 (29)	78 (31)
Carbohydrate g/d	215 (84)	214 (64)	197 (101)
Alcohol g/d	9 (15)	13(15)	8(18)
Change from baseline	n=24	n=32	n=27
Energy MJ/d	0.3 (2.9)	0.5 (2.0)	-0.4 (2.8)
Total fat g/d	29 (43)	14 (36)	28 (40)
Protein g/d	-7 (33)	3 (30)	-12 (26)
Carbohydrate g/d	-31 (74)	4 (69)	-55(81)
Alcohol g/d	-8 (22)	-5(23)	-11 (27)

*numbers do not total 94 as not all participants completed the baseline and follow up DietWebQ

For peer review only - http://bmjopen.bmj.com/site/about/guidelines.xhtml

5 January 2017 V4

Legends for figures

Figure 1

Recruitment and Flow diagram (CONSORT) for Coconut Oil, Olive Oil or Butter Trial

Figure 2

Difference (95% CI) in the primary outcome (LDL cholesterol) between each pair of randomised groups, reported in units of baseline SD. Mean (SD) change from baseline is also presented for each group in mmol/l. COB study, Intention to Treat population n=91

Figure 3

Difference (95% CI) in secondary outcomes comparing Butter vs Coconut Oil groups, reported in units of baseline SD. Mean (SD) change from baseline is also presented for each group in the natural units of the outcome. COB study, Intention to Treat population n=91. For HDL cholesterol, sign of difference and 95% CI is the opposite of that reported in Table 2, on the assumption that higher HDL is better, so the negative estimated difference (Butter vs Coconut) reported in Table 2 is presented on the side of the graph which favours the Coconut group.

Figure 4

Difference (95% CI) in secondary outcomes comparing Coconut Oil vs Olive Oil groups, reported in units of baseline SD. Mean (SD) change from baseline is also presented for each group in the natural units of the outcome. COB study, Intention to Treat population n=91. For HDL cholesterol, sign of difference and 95% CI is the opposite of that reported in Table 2, on the assumption that higher HDL is better, so the positive estimated difference (Coconut vs Olive) reported in Table 2 is presented on the side of the graph which favours the Coconut group.

Figure 5

Difference (95% CI) in secondary outcomes comparing Butter vs Olive Oil groups, reported in units of baseline SD. Mean (SD) change from baseline is also presented for each group in the natural units of the outcome. COB study, Intention to Treat population n=91. For HDL cholesterol, sign of difference and 95% CI is the opposite of that reported in Table 2, on the assumption that higher HDL is better, so the negative estimated difference (Butter vs Olive) reported in Table 2 is presented on the side of the graph which favours the Olive group.

For peer review only - http://bmjopen.bmj.com/site/about/guidelines.xhtml

For peer review only

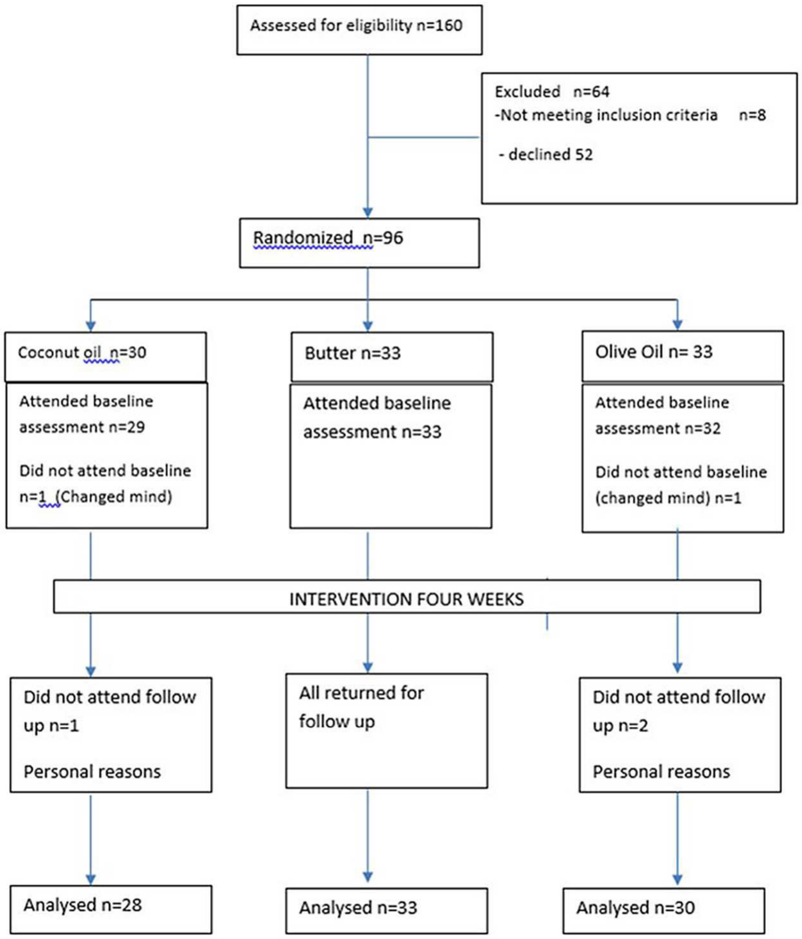

Recruitment and Flow diagram (CONSORT) for Coconut Oil, Olive Oil or Butter Trial

137x173mm (300 x 300 DPI)

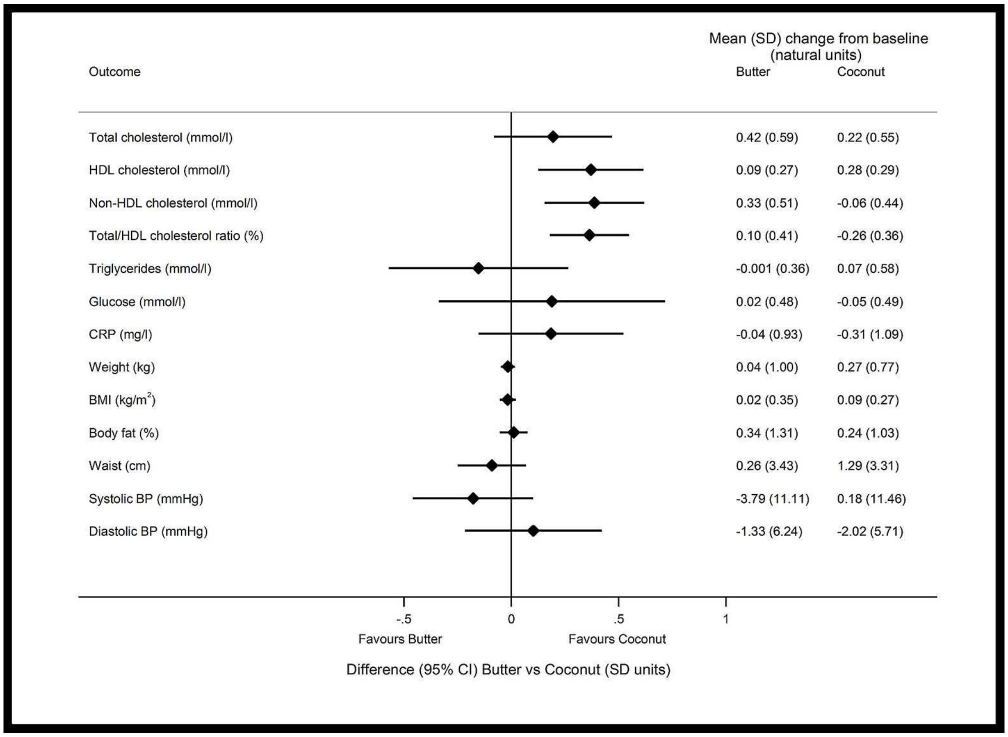

Difference (95% CI) in the primary outcome (LDL cholesterol) between each pair of randomised groups, reported in units of baseline SD. Mean (SD) change from baseline is also presented for each group in mmol/l. COB study, Intention to Treat population n=91

189x138mm (300 x 300 DPI)

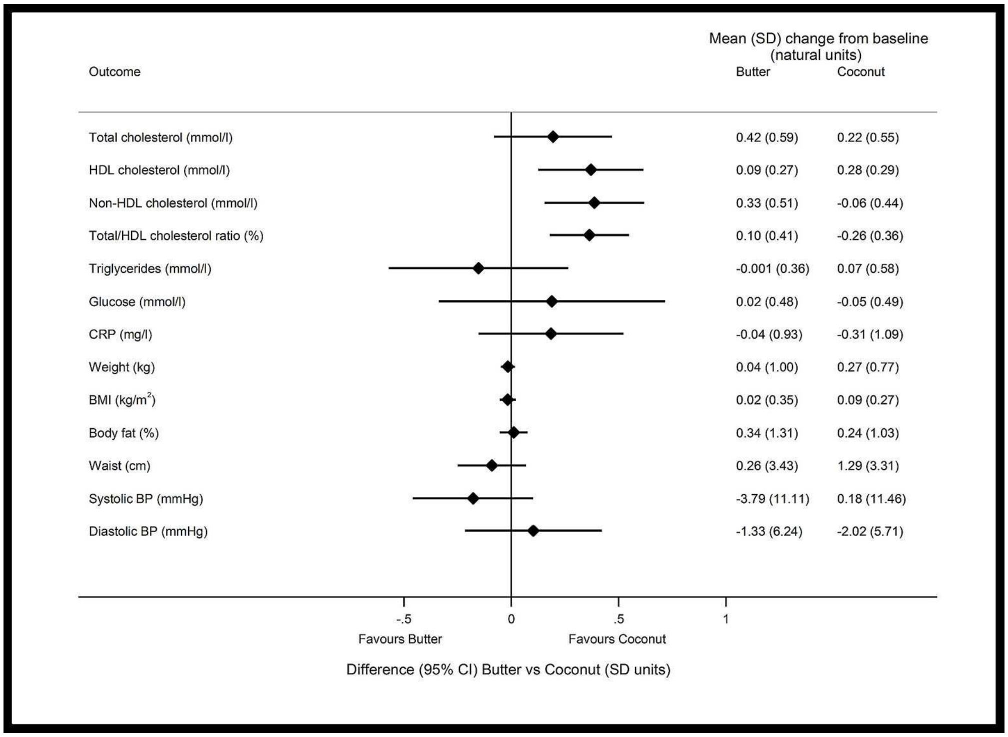

Difference (95% CI) in secondary outcomes comparing Butter vs Coconut Oil groups, reported in units of baseline SD. Mean (SD) change from baseline is also presented for each group in the natural units of the outcome. COB study, Intention to Treat population n=91. For HDL cholesterol, sign of difference and 95% CI is the opposite of that reported in Table 2, on the assumption that higher HDL is better, so the negative estimated difference (Butter vs Coconut) reported in Table 2 is presented on the side of the graph which favours the Coconut group.

189x138mm (300 x 300 DPI)

only

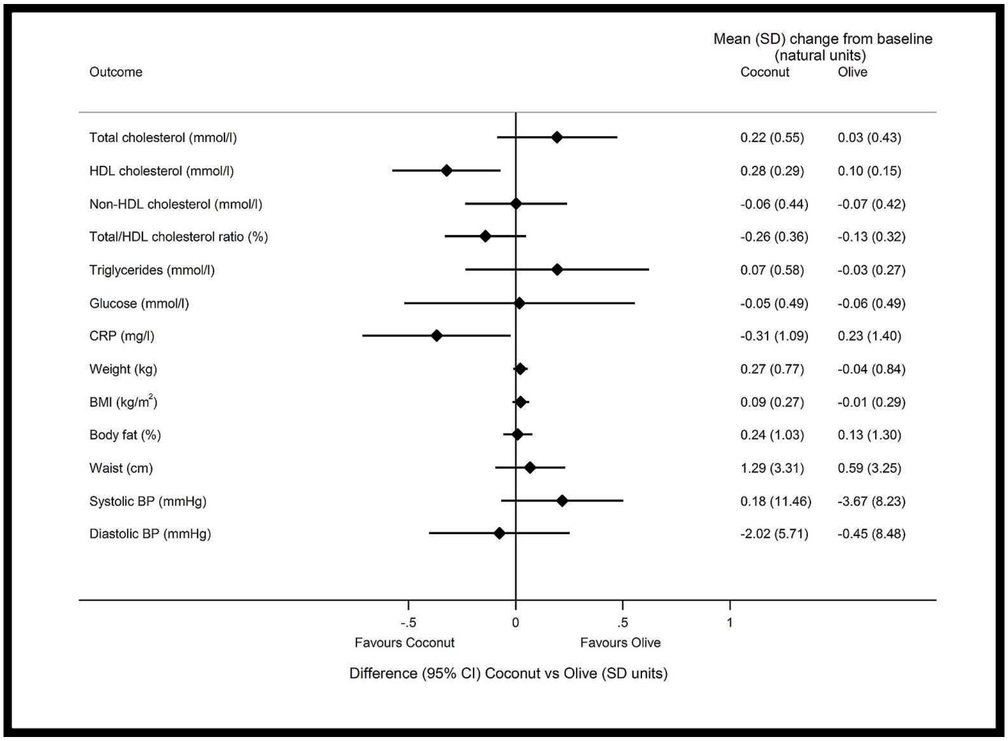

Difference (95% CI) in secondary outcomes comparing Coconut Oil vs Olive Oil groups, reported in units of baseline SD. Mean (SD) change from baseline is also presented for each group in the natural units of the outcome. COB study, Intention to Treat population n=91. For HDL cholesterol, sign of difference and 95% CI is the opposite of that reported in Table 2, on the assumption that higher HDL is better, so the positive estimated difference (Coconut vs Olive) reported in Table 2 is presented on the side of the graph which favours the Coconut group.

189x139mm (300 x 300 DPI)

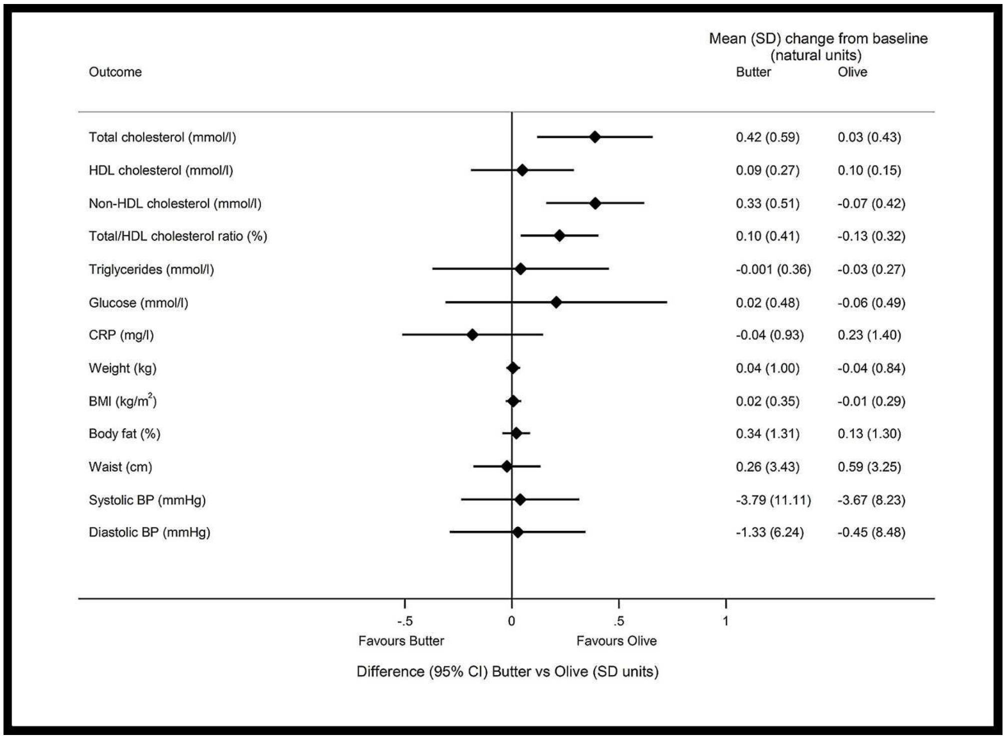

Difference (95% CI) in secondary outcomes comparing Butter vs Olive Oil groups, reported in units of baseline SD. Mean (SD) change from baseline is also presented for each group in the natural units of the outcome. COB study, Intention to Treat population n=91. For HDL cholesterol, sign of difference and 95% CI is the opposite of that reported in Table 2, on the assumption that higher HDL is better, so the negative estimated difference (Butter vs Olive) reported in Table 2 is presented on the side of the graph which favours the Olive group.

150x109mm (300 x 300 DPI)

Supplemental Table 1
Mean change in variables between baseline and follow up after dietary interventions and pairwise comparisons between fats in 91 participants (Adjusting for age, sex and body mass index at baseline)

Adjusted for age, sex and body mass index	Change from Baseline: Coconut oil N=28 Mean	Butter N=33 Mean	Olive Oil N=30 Mean	P value Comparison Between groups	Pairwise comparisons: Coconut oil vs olive oil Difference (95% CI)	Butter vs Coconut oil Difference (95% CI)	Butter vs olive oil Difference (95% CI)
LDL-Cholesterol mmol/L	-0.10	0.34	-0.06	<0.001	-0.05 (-0.28,0.18)	0.45 (0.22,0.68)	0.40 (0.17,0.62)
Total cholesterol mmol/L	0.22	0.42	0.03	0.025	0.19 (-0.09,0.46)	0.19 (-0.08,0.46)	0.38 (0.11,0.64)
HDL-Cholesterol mmol/L	0.29	0.09	0.10	0.008	0.17 (0.04,0.29)	-0.19 (-0.31,-0.06)	-0.02 (-0.14,0.10)
Triglycerides mmol/L	0.08	-0.02	-0.02	0.61	0.09 (-0.13,0.31)	-0.10 (-0.32,0.12)	-0.01 (-0.22,0.20)
Cholesterol/HDL ratio	-0.26	0.10	-0.12	0.001	-0.16 (-0.35,0.03)	0.36 (0.18,0.55)	0.20 (0.02,0.39)
Non HDL-Cholesterol mmol/L	-0.07	0.34	-0.07	<0.001	-0.01 (-0.25,0.23)	0.40 (0.17,0.64)	0.39 (0.16,0.62)
Glucose mmol/L	-0.06	0.02	-0.06	0.66	-0.01 (-0.25,0.23)	0.10 (-0.14,0.34)	0.09 (-0.14,0.33)
C-Reactive Protein mg/L	-0.29	-0.03	0.20	0.14	-0.55 (-1.08,-0.02)	0.31 (-0.22,0.84)	-0.24 (-0.75,0.27)
Weight Kg	0.27	0.05	-0.05	0.40	0.31 (-0.15,0.78)	-0.22 (-0.68,0.24)	0.10 (-0.35,0.54)
Waist cm	1.23	0.25	0.66	0.56	0.23 (-1.45,1.91)	-0.86 (-2.50,0.77)	-0.63 (-2.25,0.98)
Body fat %	0.23	0.36	0.12	0.88	-0.01 (-0.64,0.63)	0.14 (-0.48,0.76)	0.13 (-0.48,0.75)
BMI kg/m2	0.09	0.22	-0.01	0.44	0.10 (-0.06,0.26)	-0.07 (-0.23,0.09)	0.04 (-0.12,0.19)
Systolic blood pressure mm Hg	0.25	-3.68	-3.85	0.30	3.94 (-1.31,9.18)	-3.23 (-8.44,1.98)	0.70 (-4.38,5.79)
Diastolic blood pressure mm Hg	-2.08	-1.28	-0.45	0.75	-0.91 (-4.08,2.25)	1.16 (-1.96,4.29)	0.25 (-2.83,3.33)

For peer review only - http://bmjopen.bmj.com/site/about/guidelines.xhtml

For peer review only

4 January 2017 V4

Supplemental table 2

Mean change in variables between baseline and follow up after dietary interventions in 71 participants who reported no change in physical activity during the trial

	Change from Baseline			
	Coconut oil N=22 Mean (SD)	Butter N=24 Mean (SD)	Olive Oil N=25 Mean (SD)	P value Comparison Between groups
LDL-Cholesterol mmol/L	-0.10 (0.50)	0.20 (0.53)	-0.04 (0.35)	0.01
Total cholesterol mmol/L	0.19 (0.59)	0.38 (0.63)	0.07 (0.37)	0.13
HDL-Cholesterol mmol/L	0.31 (0.29)	0.10 (0.26)	0.12 (0.16)	0.001
Triglycerides mmol/L	-0.02 (0.46)	-0.01 (0.42)	-0.04 (0.23)	0.97
Cholesterol/HDL ratio	-0.30(0.35)	0.07 (0.44)	-0.13 (0.30)	0.004
Non HDL-Cholesterol mmol/L	-0.11 (0.44)	0.28 (0.56)	-0.06 (0.36)	0.008
Glucose mmol/L	-0.12 (0.49)	-0.02 (0.52)	-0.08 (0.51)	0.80
C-Reactive Protein mg/L	-0.30 (1.18)	-0.13 (0.86)	0.04 (1.00)	0.51
Weight Kg	0.13 (0.62)	0.07 (1.06)	-0.02 (0.76)	0.83
Waist cm	1.47 (3.35)	0.67 (3.48)	0.81 (3.48)	0.70
Body fat %	0.34 (1.11)	0.23 (1.37)	0.81 (1.37)	0.71
Body Mass Index kg/m2	0.04 (0.22)	0.03 (0.37)	0.00 (0.26)	0.85
Systolic blood pressure mm Hg	-3.1 (8.9)	-5.1 (11.3)	-2.4 (7.8)	0.60
Diastolic blood pressure mm Hg	-2.4 (5.6)	-2.0 (6.6)	0.8 (8.4)	0.24

For peer review only

For peer review only - http://bmjopen.bmj.com/site/about/guidelines.xhtml

4 January 2017 V4

For peer review only

For peer review only - http://bmjopen.bmj.com/site/about/guidelines.xhtml

Appendix 1: Fatty acid composition of fats

Samples of the fats/oils used in the trial were sent for fatty acid composition to West Yorkshire Analytical Services, a UKAS accredited testing service for food composition. The results are tabulated below.

Coconut oil was 94 % saturated fatty acids, of which the main components were lauric acid C12:0 (48%) and myristic acid C14:0 (19%), palmitic acid C16:0 (9%) and caprylic acid C8:0 (9%); and 5% mono unsaturated fat, mainly oleic acid C18:1n9 (5%).

Butter was 66% saturated fatty acids, of which the main components were palmitic acid C16:0 (28%), stearic acid C18:0 (12%), myristic acid C14:0 (11%); 26% monounsaturated fat, mainly oleic acid C18:1n9 (22%); and 3% polyunsaturated fat, linoleic acid C18:2n6 (2%) and alpha-linolenic acid (1%).

Olive oil was 19% saturated fatty acids, mainly palmitic acid C16:0, 15% with stearic acid C18:0 (3%); 68% monounsaturates with the main component being oleic acid C18:1n9 (64%); and 13% polyunsaturates Linoleic acid C18:2n6 (12%).

		Coconut oil	Olive Oil	Butter
		% composition	% composition	% composition
C4:0	Butyric acid	<1	<0.1	2.5
C6:0	Caproic acid	0.7	<0.1	1.9
C8:0	Caprylic acid	8.6	<0.1	1.2
C10:0	Capric acid	6.3	<0.1	2.5
C12:0	Lauric acid	47.6	<0.1	3
C14:0	Myristic acid	18.6	<0.1	10.6
C14:1		<0.1	<0.1	0.9
C15:0		<0.1	<0.1	1.1
C16:0	Palmitic acid	8.6	14.8	28.1
C16:1	Palmitoleic acid	<0.1	1.5	1.4
C17:0		<0.1	<0.1	0.6
C17:1		<0.1	<0.1	0.4
C18.0	Stearic Acid	3.4	3	12.4
C18:1t			<0.1	3.2
C18:1n9	Oleic Acid	5.2	63.5	22.2
C181n7	cis-Vaccenic Acid	<0.1	2.8	0.4

For peer review only - http://bmjopen.bmj.com/site/about/guidelines.xhtml

11 December 2017 V3

C18:2tt		<0.1	<0.1	0.5
C18:2ct		<0.1	<0.1	0.1
C18:2tc		<0.1	<0.1	0.2
C18:2n6	Linoleic Acid	0.8	11.9	1.9
C18:3n6	Gamma Linolenic Acid	<0.1	<0.1	<0.1
C18:3n3	Alpha-Linolenic Acid	<0.1	<0.1	0.9
C20:0	Arachidic acid	<0.1	<0.1	0.2
C20:2n6	Eicosadienoic acid	<0.1	<0.1	<0.1
C18:4n3	Stearidonic acid	<0.1	0.2	0.1
C20:1	Paullinic acid	<0.1	<0.1	<0.1
C22:0	Behenic Acid	<0.1	0.2	0.1
C22:1n9	Erucic Acid	<0.1	<0.1	0.1
C22:2	Docosadienoic acid	<0.1	0.6	<0.1
C24:0	Lignoceric acid	<0.1	<0.1	<0.1
	Saturates	93.9	18.6	66.2
	Monounsaturates	5.2	68	26.1
	Polyunsaturates	0.7	13.5	3.4
	Transesters	<0.1	<0.1	4.2

For peer review only

For peer review only - http://bmjopen.bmj.com/site/about/guidelines.xhtml

CONSORT 2010 checklist of information to include when reporting a randomised trial*

Section/Topic	Item No	Checklist item	Reported on page No
**Title and abstract**			
	1a	Identification as a randomised trial in the title	1
	1b	Structured summary of trial design, methods, results, and conclusions (for specific guidance see CONSORT for abstracts)	2,3
**Introduction**			
Background and objectives	2a	Scientific background and explanation of rationale	5,6
	2b	Specific objectives or hypotheses	6
**Methods**			
Trial design	3a	Description of trial design (such as parallel, factorial) including allocation ratio	7,8
	3b	Important changes to methods after trial commencement (such as eligibility criteria), with reasons	N/A
Participants	4a	Eligibility criteria for participants	7
	4b	Settings and locations where the data were collected	7,8
Interventions	5	The interventions for each group with sufficient details to allow replication, including how and when they were actually administered	7,8
Outcomes	6a	Completely defined pre-specified primary and secondary outcome measures, including how and when they were assessed	8,9
	6b	Any changes to trial outcomes after the trial commenced, with reasons	N/A
Sample size	7a	How sample size was determined	9,10
	7b	When applicable, explanation of any interim analyses and stopping guidelines	N/A
Randomisation:			
Sequence generation	8a	Method used to generate the random allocation sequence	7
	8b	Type of randomisation; details of any restriction (such as blocking and block size)	7
Allocation concealment mechanism	9	Mechanism used to implement the random allocation sequence (such as sequentially numbered containers), describing any steps taken to conceal the sequence until interventions were assigned	7
Implementation	10	Who generated the random allocation sequence, who enrolled participants, and who assigned participants to interventions	7
Blinding	11a	If done, who was blinded after assignment to interventions (for example, participants, care providers, those	8,9

For peer review only - http://bmjopen.bmj.com/site/about/guidelines.xhtml

		assessing outcomes) and how	
	11b	If relevant, description of the similarity of interventions	N/A
Statistical methods	12a	Statistical methods used to compare groups for primary and secondary outcomes	9,10
	12b	Methods for additional analyses, such as subgroup analyses and adjusted analyses	9
**Results**			
Participant flow (a diagram is strongly recommended)	13a	For each group, the numbers of participants who were randomly assigned, received intended treatment, and were analysed for the primary outcome	Figure 1
	13b	For each group, losses and exclusions after randomisation, together with reasons	Figure 1
Recruitment	14a	Dates defining the periods of recruitment and follow-up	2,7,8
	14b	Why the trial ended or was stopped	8
Baseline data	15	A table showing baseline demographic and clinical characteristics for each group	Figure 1, table 1
Numbers analysed	16	For each group, number of participants (denominator) included in each analysis and whether the analysis was by original assigned groups	Table 2, Figures 2-5
Outcomes and estimation	17a	For each primary and secondary outcome, results for each group, and the estimated effect size and its precision (such as 95% confidence interval)	Table 2, figures 2-6
	17b	For binary outcomes, presentation of both absolute and relative effect sizes is recommended	N/A
Ancillary analyses	18	Results of any other analyses performed, including subgroup analyses and adjusted analyses, distinguishing pre-specified from exploratory	12
Harms	19	All important harms or unintended effects in each group (for specific guidance see CONSORT for harms)	12
**Discussion**			
Limitations	20	Trial limitations, addressing sources of potential bias, imprecision, and, if relevant, multiplicity of analyses	17,18
Generalisability	21	Generalisability (external validity, applicability) of the trial findings	18
Interpretation	22	Interpretation consistent with results, balancing benefits and harms, and considering other relevant evidence	19
**Other information**			
Registration	23	Registration number and name of trial registry	1,3
Protocol	24	Where the full trial protocol can be accessed, if available	3
Funding	25	Sources of funding and other support (such as supply of drugs), role of funders	21

*We strongly recommend reading this statement in conjunction with the CONSORT 2010 Explanation and Elaboration for important clarifications on all the items. If relevant, we also recommend reading CONSORT extensions for cluster randomised trials, non-inferiority and equivalence trials, non-pharmacological treatments, herbal interventions, and pragmatic trials. Additional extensions are forthcoming: for those and for up to date references relevant to this checklist, see www.consort-statement.org.

For peer review only - http://bmjopen.bmj.com/site/about/guidelines.xhtml
